# Supplementary material for: Antibacterial and antioxidant activities of plants consumed by western lowland gorilla (Gorilla gorilla gorilla) in Gabon
Source: PLoS One. 2024 Sep 11;19(9):e0306957. doi: 10.1371/journal.pone.0306957 (PMC11389915; doi:10.1371/journal.pone.0306957)
Supplement: S1 File — (DOCX) [file pone.0306957.s002.docx]

Supplementary information

**
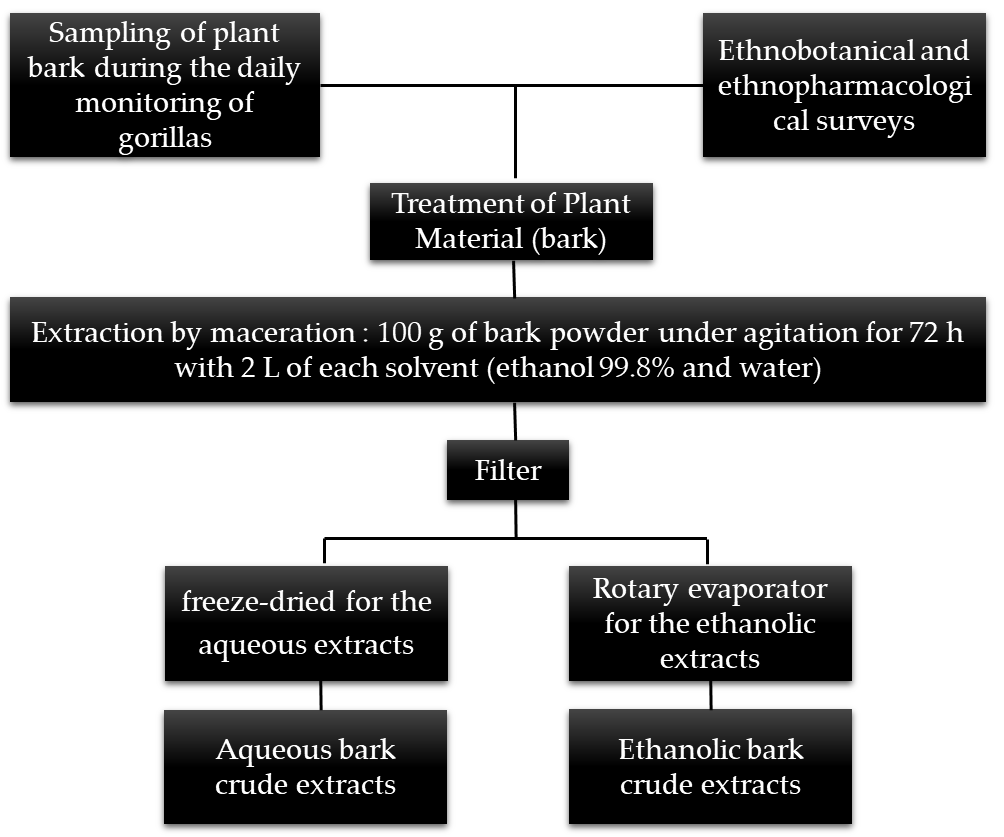
**

Figure 2: Sequential extraction procedure of the four selected bark crude extracts.

**Table 2:** Ethnobotanical and ethnopharmacological informations about selected plants, consumed by western lowland gorilla living MDNP and used as medicinal plants (MPs) in traditional medicine by healers in Gabon.

| **Local name in** **Vungu** | **Botanical name** | **Family** | **Parts used** | **Mode/form of consumption** | **Medicinal Applications** | **References** |
| --- | --- | --- | --- | --- | --- | --- |
| Iroko ou Kambala | ***Milicia excelsa*** | Moraceae | Fruits, seeds, leaves, and bark | Raw, dried, semicooked, decoction | Antioxidant, antimicrobial, anti-inflammatory, gastrointestinal, calmative, analgesic | [[1-6](#_ENREF_1)] |
| Divevengui blanc | ***Ficus* ssp** | Moraceae | Milky latex, fruits and bark | Raw, dried, decoction, eaten, fresh | Antioxidant, antimicrobial, anti-inflammatory, antiviral, antidiarrhoeal, antifungal, anticancer, antiplasmodial, antiulcer, lannoite | [[7](#_ENREF_7), [8](#_ENREF_8)] |
| Mububa | ***Myrianthus arboreus*** | Moraceae (Cecropiaceae) | Fruits, seeds, leaves, and bark | Raw, decoction, and dried | Antioxidant, antimicrobial, anti-inflammatory, antifungal, antidiabetic, antinociceptive | [[9-15](#_ENREF_9)] |
| Mufuma | ***Ceiba pentandra*** | Malvaceae (Bombacaceae) | Fruits, seeds, leaves, and bark | Raw, decoction, and dried | Antioxidant, antimicrobial, anti-inflammatory, anticancer, analgesic, anti-hyperglycemic | [[16-19](#_ENREF_16)] |

**Legend:** Vungu, also called ghivungu, is a Bantu language spoken by the Vungus in Gabon, including in MDNP[[19](#_ENREF_19)]

**Table 3.** Percentage yield of 100 g of bark in 2000 L solvent from *Ceiba pentandra*, *Myrianthus arboreus*, *Ficus* *ssp* and *Milicia excelsa* BCE.

| **Botanical name** | **Extract mass (g)** | **% Yield (g)** |
| --- | --- | --- |
| ***Ceiba Pentandra*** |  |  |
| AE | 1.93 | 1.93 ± 0.11^a^ |
| EE | 1.58 | 1.58 ± 0.12^b^ |
| ***Myrianthus arboreus*** |  |  |
| AE | 2.76 | 2.76 ± 0.23^c^ |
| EE | 1.15 | 1.15 ± 0.14^d^ |
| ***Ficus* ssp** |  |  |
| AE | 2.26 | 2.26 ± 0.27^e^ |
| EE | 1.31 | 1.31 ± 0.21^f^ |
| ***Milicia excelsa*** |  |  |
| AE | 1.52 | 1.52 ± 0.33^g^ |
| EE | 1.51 | 1.51 ± 0.22^h^ |

**Legend:** EE: Ethanol extract; AE: Aqueous extract. Values are means ± standard deviation of triplicate analysis. In all cases, the difference between extraction methods is statistically significant (P< 0.05).

**DPPH assay**

**Calibration curves**

Ascorbic acid calibration curve

|  | Ascorbic acid |  |  |  |  |  |
| --- | --- | --- | --- | --- | --- | --- |
|  | S1 | S2 | S3 | S4 | S5 |  |
|  | 0.358 | 0.566 | 0.95 | 0.788 | 1.042 |  |
|  | 0.291 | 0.68 | 0.898 | 1.06 | 1.248 |  |
|  | 0.445 | 0.876 | 0.881 | 1.038 | 1,,955 |  |
|  | 0.36466667 | 0.70733333 | 0.90966667 | 0.962 | 1.145 | M |
|  | 80.6542882 | 62.4756852 | 51.7418214 | 48.9655172 | 39.2572944 | % |
| X (μg/ml) | 6.25 | 3.125 | 1.5625 | 0.78125 | 0.35 |  |
|  |  |  |  |  |  |  |
|  |  |  |  |  |  |  |
| X (μg/ml) | 6.25 | 3.125 | 1.5625 | 0.78125 | 0.35 |  |
| Y (%) | 80.6542882 | 62.4756852 | 51.7418214 | 48.9655172 | 39.2572944 |  |
|  |  |  |  |  |  |  |
| IC50 | 1.39674247 |  |  |  |  |  |
| AAI | 17.8993341 |  |  |  |  |  |

**Legend: IC50:** Median inhibitory concentration; **AAI**: Antioxydant Activity Index.

Figure S1: Ascorbic acid calibration curve

***Ceiba pentandra*** AE calibration curve

|  |  |  |  |  |  |
| --- | --- | --- | --- | --- | --- |
|  |  |  | Mufuma Aq |  |  |
| C | 6.25 | 3.125 | 1.5625 | 0.78125 | 0.35 |
| 0D1 | 0.581 | 0.796 | 0.992 | 1.076 | 1.223 |
| 0D2 | 0.683 | 0.833 | 1.004 | 1.143 | 1.321 |
| 0D3 | 0.729 | 0.953 | 1.103 | 1.254 | 1.441 |
| Average value | 0.66433333 | 0.86066667 | 1.033 | 1.15766667 | 1.32833333 |
| Percentage of inhibition | 64.7568523 | 54.3412909 | 45.198939 | 38.5853227 | 29.5313882 |
|  |  |  |  |  |  |
|  |  |  |  |  |  |
|  |  |  |  |  |  |
| X (µg/mL ) | 6.25 | 3.125 | 1.5625 | 0.78125 | 0.35 |
| Y (%) | 64.7568523 | 54.3412909 | 45.198939 | 38.5853227 | 29.5313882 |
|  |  |  |  |  |  |
| IC50 | 3.05911276 |  |  |  |  |
| AAI | 8.19672131 |  |  |  |  |

**Legend: C:** Concentration; **OD:** Optical Density; **IC50:** Median inhibitory concentration; **AAI**: Antioxydant Activity Index.

Figure S2: ***Ceiba pentandra*** AE calibration curve

***Ceiba pentandra*** EtOH calibration curve

|  |  |  | Mufuma EtOH | |  |
| --- | --- | --- | --- | --- | --- |
| C | 6.25 | 3.125 | 1.5625 | 0.78125 | 0.35 |
| 0D1 | 0.399 | 0.731 | 0.972 | 1.523 | 1.258 |
| 0D2 | 0.459 | 0.812 | 1.232 | 0.889 | 1.596 |
| OD3 | 0.469 | 0.951 | 1.541 | 1.02 | 1.754 |
| Average value | 0.44233333 | 0.83133333 | 1.24833333 | 1.144 | 1.536 |
| Percentage of inhibition | 76.5340407 | 55.8974359 | 33.77542 | 39.3103448 | 18.5145889 |
|  |  |  |  |  |  |
| X (μg/ml) | 6.25 | 3.125 | 1.5625 | 0.78125 | 0.35 |
| Y (%) | 76.5340407 | 55.8974359 | 33.77542 | 39.3103448 | 18.5145889 |
|  |  |  |  |  |  |
| IC50 | 2.88152894 |  |  |  |  |
| AAI | 8.65051903 |  |  |  |  |

**Legend: C:** Concentration; **OD:** Optical Density; **IC50:** Median inhibitory concentration; **AAI**: Antioxydant Activity Index.

Figure S3: ***Ceiba pentandra*** EtOH calibration curve

***Myrianthus arboreus*** AE calibration curve

|  |  |  | Muboba Aq |  |  |
| --- | --- | --- | --- | --- | --- |
| C | 6.25 | 3.125 | 1.5625 | 0.78125 | 0.35 |
| 0D1 | 0.604 | 0.796 | 0.891 | 1.002 | 1.304 |
| 0D2 | 0.508 | 0.592 | 0.988 | 1.409 | 1.001 |
| OD3 | 0.405 | 0.696 | 0.999 | 1.207 | 1.508 |
| Average value | 0.50566667 | 0.69466667 | 0.95933333 | 1.206 | 1.271 |
| Percentage of inhibition | 73.1741821 | 63.1476569 | 49.106985 | 36.0212202 | 32.5729443 |
|  |  |  |  |  |  |
|  |  |  |  |  |  |
|  |  |  |  |  |  |
| X (µg/mL ) | 6.25 | 3.125 | 1.5625 | 0.78125 | 0.35 |
| Y (%) | 73.1741821 | 63.1476569 | 49.106985 | 36.0212202 | 32.5729443 |
|  |  |  |  |  |  |
| IC50 | 2.29715627 |  |  |  |  |
| AAI | 10.8832876 |  |  |  |  |

**Legend: C:** Concentration; **OD:** Optical Density; **IC50:** Median inhibitory concentration; **AAI**: Antioxydant Activity Index.

Figure S4: ***Myrianthus arboreus*** AE calibration curve

***Myrianthus arboreus*** EtOH calibration curve

|  |  |  | Muboba EtOH | |  |
| --- | --- | --- | --- | --- | --- |
| C | 6.25 | 3.125 | 1.5625 | 0.78125 | 0.35 |
| 0D1 | 0.534 | 0.793 | 1.423 | 1.427 | 1.344 |
| 0D2 | 0.796 | 0.812 | 0.918 | 1.013 | 0.912 |
| OD3 | 0.636 | 0.803 | 0.899 | 0.994 | 0.998 |
| Average value | 0.65533333 | 0.80266667 | 1.08 | 1.14466667 | 1.08466667 |
| Percentage of inhibition | 65.2343059 | 57.418214 | 42.7055703 | 39.2749779 | 42.4580018 |
|  |  |  |  |  |  |
|  |  |  |  |  |  |
|  |  |  |  |  |  |
| X (µg/mL ) | 6.25 | 3.125 | 1.5625 | 0.78125 | 0.35 |
| Y (%) | 65.2343059 | 57.418214 | 42.7055703 | 39.2749779 | 42.4580018 |
|  |  |  |  |  |  |
| IC50 | 2.54291107 |  |  |  |  |
| AAI | 9.83129498 |  |  |  |  |

**Legend: C:** Concentration; **OD:** Optical Density; **IC50:** Median inhibitory concentration; **AAI**: Antioxydant Activity Index.

Figure S5: ***Myrianthus arboreus*** EtOH calibration curve

***Milicia excelsa* AE calibration curve**

|  |  |  | Iroko Aq |  |  |
| --- | --- | --- | --- | --- | --- |
| C | 6.25 | 3.125 | 1.5625 | 0.78125 | 0.35 |
| 0D1 | 0.605 | 0.705 | 0.953 | 1.009 | 1.003 |
| 0D2 | 0.741 | 0.812 | 0.995 | 1.119 | 1.108 |
| OD3 | 0.704 | 0.903 | 1.015 | 1.206 | 1.207 |
| Average value | 0.68333333 | 0.80666667 | 0.98766667 | 1.11133333 | 1.106 |
| Percentage of inhibition | 63.7488948 | 57.2060124 | 47.6038904 | 41.0433245 | 41.3262599 |
|  |  |  |  |  |  |
|  |  |  |  |  |  |
|  |  |  |  |  |  |
| X (µg/mL ) | 6.25 | 3.125 | 1.5625 | 0.78125 | 0.35 |
| Y (%) | 63.7488948 | 57.2060124 | 47.6038904 | 41.0433245 | 41.3262599 |
|  |  |  |  |  |  |
| IC50 | 2.36797822 |  |  |  |  |
| AAI | 10.5932203 |  |  |  |  |

**Legend: C:** Concentration; **OD:** Optical Density; **IC50:** Median inhibitory concentration; **AAI**: Antioxydant Activity Index.

Figure S6: ***Milicia excelsa*** AE calibration curve

***Milicia excelsa* EtOH calibration curve**

|  |  |  | Iroko EtOH |  |  |
| --- | --- | --- | --- | --- | --- |
| C | 6.25 | 3.125 | 1.5625 | 0.78125 | 0.35 |
| 0D1 | 0.406 | 0.776 | 0.845 | 0.922 | 1.501 |
| 0D2 | 0.586 | 0.641 | 0.901 | 1.223 | 1.404 |
| OD3 | 0.393 | 0.514 | 1.143 | 1.433 | 0.774 |
| Average value | 0.46166667 | 0.64366667 | 0.963 | 1.19266667 | 1.22633333 |
| Percentage of inhibition | 75.5083996 | 65.8532272 | 48.9124668 | 36.7285588 | 34.9425287 |
|  |  |  |  |  |  |
|  |  |  |  |  |  |
|  |  |  |  |  |  |
| X (µg/mL ) | 6.25 | 3.125 | 1.5625 | 0.78125 | 0.35 |
| Y (%) | 75.5083996 | 65.8532272 | 48.9124668 | 36.7285588 | 34.9425287 |
|  |  |  |  |  |  |
| IC50 | 2.07811183 |  |  |  |  |
| AAI | 12.0302199 |  |  |  |  |

**Legend: C:** Concentration; **OD:** Optical Density; **IC50:** Median inhibitory concentration; **AAI**: Antioxydant Activity Index.

Figure S7: ***Milicia excelsa* EtOH** calibration curve

***Ficus* ssp AE calibration curve**

|  |  |  | Divevengui Aq | |  |
| --- | --- | --- | --- | --- | --- |
| C | 6.25 | 3.125 | 1.5625 | 0.78125 | 0.35 |
| 0D1 | 0.604 | 0.796 | 0.891 | 1.002 | 1.304 |
| 0D2 | 0.508 | 0.582 | 0.988 | 1.409 | 1.001 |
| OD3 | 0.405 | 0.696 | 0.999 | 1.207 | 1.508 |
| Average value | 0.50566667 | 0.69133333 | 0.95933333 | 1.206 | 1.271 |
| Percentage of inhibition | 73.1741821 | 63.3244916 | 49.106985 | 36.0212202 | 32.5729443 |
|  |  |  |  |  |  |
|  |  |  |  |  |  |
|  |  |  |  |  |  |
| X (µg/mL ) | 6.25 | 3.125 | 1.5625 | 0.78125 | 0.35 |
| Y (%) | 73.1741821 | 63.3244916 | 49.106985 | 36.0212202 | 32.5729443 |
|  |  |  |  |  |  |
| IC50 | 2.29214036 |  |  |  |  |
| AAI | 10.9070285 |  |  |  |  |

**Legend: C:** Concentration; **OD:** Optical Density; **IC50:** Median inhibitory concentration; **AAI**: Antioxydant Activity Index.

Figure S8: ***Ficus* ssp** AE calibration curve

***Ficus* ssp EtOH calibration curve**

|  |  |  | Divevengui EtOH | |  |
| --- | --- | --- | --- | --- | --- |
| C | 6.25 | 3.125 | 1.5625 | 0.78125 | 0.35 |
| 0D1 | 0.353 | 0.739 | 0.803 | 1.201 | 1.055 |
| 0D2 | 0.408 | 0.891 | 0.903 | 0.903 | 1.012 |
| OD3 | 0.605 | 0.937 | 0.905 | 0.917 | 1.005 |
| Average value | 0.45533333 | 0.85566667 | 0.87033333 | 1.007 | 1.024 |
| Percentage of inhibition | 75.8443855 | 54.6065429 | 53.8284704 | 46.5782493 | 45.6763926 |
|  |  |  |  |  |  |
|  |  |  |  |  |  |
|  |  |  |  |  |  |
| X (µg/mL ) | 6.25 | 3.125 | 1.5625 | 0.78125 | 0.35 |
| Y (%) | 75.8443855 | 54.6065429 | 53.8284704 | 46.5782493 | 45.6763926 |
|  |  |  |  |  |  |
| IC50 | 1.34607646 |  |  |  |  |
| AAI | 18.5735513 |  |  |  |  |

**Legend: C:** Concentration; **OD:** Optical Density; **IC50:** Median inhibitory concentration; **AAI**: Antioxydant Activity Index.

Figure S9: ***Ficus* ssp** EtOH calibration curve

**IC_(50)_ AAI**

|  |  |  |  |  | Mufuma EtOH | |  |  |  |
| --- | --- | --- | --- | --- | --- | --- | --- | --- | --- |
|  |  |  |  |  |  | 1.885 |  |  |  |
|  |  |  |  |  |  | IC 50 |  |  | AAI |
| C | 6.25 | 3.125 | 1.5625 | 0.78125 | 0.35 |  |  |  |  |
| 0D1 | 0.399 | 0.731 | 0.972 | 1.523 | 1.258 | 2.61581599 | y = 8.9517x + 26.584 |  | 9.57854406 |
| P1 | 78.8328912 | 61.2201592 | 48.4350133 | 19.204244 | 33.2625995 |  | R² = 0.8439 |  |  |
| 0D2 | 0.459 | 0.812 | 1.232 | 0.889 | 1.596 | 1.80529285 | y = 17.57ln(x) + 39.621 |  | 13.8888889 |
| P2 | 75.6498674 | 56.9230769 | 34.6419098 | 52.8381963 | 15.331565 |  | R² = 0.7493 |  |  |
| OD3 | 0.469 | 0.951 | 1.541 | 1.02 | 1.754 | 3.54869756 | y = 9.559x + 16.078 |  | 7.06214689 |
| P3 | 75.1193634 | 49.5490716 | 18.2493369 | 45.8885942 | 6.94960212 |  | R² = 0.7155 |  |  |
| Average value |  |  |  |  |  | 2.65660213 |  |  | 10.1765266 |
| SD |  |  |  |  |  | 0.87241769 |  |  | 3.45243232 |
|  |  |  |  |  |  |  |  |  |  |
|  |  |  |  |  |  |  |  |  |  |
|  |  |  |  |  |  |  |  |  |  |
|  |  |  | Mufuma Aq |  |  | IC 50 |  |  | AAI |
| C | 6.25 | 3.125 | 1.5625 | 0.78125 | 0.35 |  |  |  |  |
| 0D1 | 0.581 | 0.796 | 0.992 | 1.076 | 1.223 | 2.32643772 | y = 5.4044x + 37.427 |  | 10.7758621 |
| P1 | 69.1777188 | 57.7718833 | 47.3740053 | 42.9177719 | 35.1193634 |  | R² = 0.9468 |  |  |
| 0D2 | 0.683 | 0.833 | 1.004 | 1.143 | 1.321 | 2.96958821 | y = 5.1822x + 34.611 |  | 8.44594595 |
| P2 | 63.7665782 | 55.8090186 | 46.7374005 | 39.3633952 | 29.9204244 |  | R² = 0.8666 |  |  |
| OD3 | 0.729 | 0.953 | 1.103 | 1.254 | 1.441 | 2.88996284 | y = 12.785ln(x) + 36.432 |  | 8.68055556 |
| P3 | 61.3262599 | 49.4429708 | 41.4854111 | 33.4748011 | 23.5543767 |  | R² = 0.9937 |  |  |
| Average value |  |  |  |  |  | 2.72866292 |  |  | 9.30078786 |
| SD |  |  |  |  |  | 0.35060503 |  |  | 1.28282632 |
|  |  |  |  |  |  |  |  |  |  |
|  |  |  |  |  |  |  |  |  |  |
|  |  |  |  |  |  |  |  |  |  |
|  |  |  |  |  |  |  |  |  |  |
|  |  |  |  |  | Mububa EtOH | |  |  |  |
|  |  |  |  |  |  | 1.885 |  |  |  |
|  |  |  |  |  |  | IC 50 |  |  | AAI |
| C | 6.25 | 3.125 | 1.5625 | 0.78125 | 0.35 |  |  |  |  |
| 0D1 | 0.534 | 0.793 | 1.423 | 1.427 | 1.344 | 3.41279639 | y = 8.5868x + 20.695 |  | 7.3313783 |
| P1 | 71.6710875 | 57.9310345 | 24.5092838 | 24.2970822 | 28.7002653 |  | R² = 0.8744 |  |  |
| 0D2 | 0.796 | 0.812 | 0.918 | 1.013 | 0.912 | 1.95550289 | y = 1.5915x + 48.933 |  | 12.8205128 |
| P2 | 57.7718833 | 56.9230769 | 51.2997347 | 46.2599469 | 51.6180371 |  | R² = 0,6572 |  |  |
| OD3 | 0.636 | 0.803 | 0.899 | 0.994 | 0.998 | 1.19597839 | y = 3.3322x + 46.015 |  | 21.0084034 |
| P3 | 66.2599469 | 57.4005305 | 52.3076923 | 47.2679045 | 47.0557029 |  | R² = 0.9833 |  |  |
| Average value |  |  |  |  |  | 2.18809255 |  |  | 13.7200982 |
| SD |  |  |  |  |  | 1.1265629 |  |  | 6.88274611 |
|  |  |  |  |  |  |  |  |  |  |
|  |  |  |  |  |  |  |  |  |  |
|  |  |  |  |  |  |  |  |  |  |
|  |  |  | Mububa Aq |  |  | IC 50 |  |  | AAI |
| C | 6.25 | 3.125 | 1.5625 | 0.78125 | 0.35 |  |  |  |  |
| 0D1 | 0.604 | 0.796 | 0.891 | 1.002 | 1.304 | 2.17373429 | y = 5.1078x + 38.897 |  | 11.5207373 |
| P1 | 67.9575597 | 57.7718833 | 52.7320955 | 46.8435013 | 30.8222812 |  | R² = 0.7845 |  |  |
| 0D2 | 0.508 | 0.592 | 0.988 | 1.409 | 1.001 | 2.06798566 | y = 6.5808x + 36.391 |  | 12.1359223 |
| P2 | 73.0503979 | 68.5941645 | 47.5862069 | 25.2519894 | 46.8965517 |  | R² = 0.6697 |  |  |
| OD3 | 0.405 | 0.696 | 0.999 | 1.207 | 1.508 | 2.12433983 | y = 20.139ln(x) + 40.367 |  | 11.7924528 |
| P3 |  | 63.0769231 | 47.0026525 | 35.9681698 | 20 |  | R² = 0.9961 |  |  |
| Average value |  |  |  |  |  | 2.12201993 |  |  | 11.8163708 |
| SD |  |  |  |  |  | 0.05291247 |  |  | 0.30828915 |
|  |  |  |  |  |  |  |  |  |  |
|  |  |  |  |  |  |  |  |  |  |
|  |  |  |  |  |  |  |  |  |  |
|  |  |  |  |  |  |  |  |  |  |
|  |  |  |  |  | Iroko EtOH |  |  |  |  |
|  |  |  |  |  |  | 1.885 |  |  |  |
|  |  |  |  |  |  | IC 50 |  |  | AAI |
| C | 6.25 | 3.125 | 1.5625 | 0.78125 | 0.35 |  |  |  |  |
| 0D1 | 0.406 | 0.776 | 0.845 | 0.922 | 1.501 | 1.85821187 | y = 17.477ln(x) + 45.369 |  | 13.5135135 |
| P1 | 78.4615385 | 58.8328912 | 55.1724138 | 51.0875332 | 20.3713528 |  | R² = 0.8908 |  |  |
| 0D2 | 0.586 | 0.641 | 0.901 | 1.223 | 1.404 | 3.16537544 | y = 16.449ln(x) + 42.569 |  | 7.91139241 |
| P2 | 68.9124668 | 65.994695 | 52.2015915 | 35.1193634 | 25.5172414 |  | R² = 0.963 |  |  |
| OD3 | 0.393 | 0.514 | 1.143 | 1.433 | 0.774 | 1.72750635 | y = 7.0431x + 37.833 |  | 14.5348837 |
| P3 | 79.1511936 | 72.7320955 | 39.3633952 | 23.9787798 | 58.938992 |  | R² = 0.5352 |  |  |
| Average value |  |  |  |  |  | 2.25036455 |  |  | 11.9865965 |
| SD |  |  |  |  |  | 0.79511299 |  |  | 3.56598736 |
|  |  |  |  |  |  |  |  |  |  |
|  |  |  |  |  |  |  |  |  |  |
|  |  |  |  |  |  |  |  |  |  |
|  |  |  | Iroko Aq |  |  | IC 50 |  |  | AAI |
| C | 6.25 | 3.125 | 1.5625 | 0.78125 | 0.35 |  |  |  |  |
| 0D1 | 0.581 | 0.796 | 0.992 | 1.076 | 1.223 | 2.32643772 | y = 5.4044x + 37.427 |  | 10.7758621 |
| P1 | 69.1777188 | 57.7718833 | 47.3740053 | 42.9177719 | 35.1193634 |  | R² = 0.9468 |  |  |
| 0D2 | 0.683 | 0.833 | 1.004 | 1.143 | 1.321 | 13.2454187 | y = 5.1822x + 34.611 |  | 1.88821752 |
| P2 | 63.7665782 | 55.8090186 | 46.7374005 | 39.3633952 | 29.9204244 |  | R² = 0.8666 |  |  |
| OD3 | 0.729 | 0.953 | 1.103 | 1.254 | 1.441 | 2.88996284 | y = 5.7651x + 27.941 |  | 8.68055556 |
| P3 | 61.3262599 | 49.4429708 | 41.4854111 | 33.4748011 | 23.5543767 |  | R² = 0.9028 |  |  |
| Average value |  |  |  |  |  | 6.15393974 |  |  | 7.11487838 |
| SD |  |  |  |  |  | 6.14786102 |  |  | 4.64608062 |
|  |  |  |  |  |  |  |  |  |  |
|  |  |  |  |  |  |  |  |  |  |
|  |  |  |  |  |  |  |  |  |  |
|  |  |  |  |  |  |  |  |  |  |
|  |  |  |  |  | Div EtOH |  |  |  |  |
|  |  |  |  |  |  | 1.885 |  |  |  |
|  |  |  |  |  |  | IC 50 |  |  | AAI |
| C | 6.25 | 3.125 | 1.5625 | 0.78125 | 0.35 |  |  |  |  |
| 0D1 | 0.353 | 0.739 | 0.803 | 1.201 | 1.055 | 1.54064868 | y = 6.8231x + 39.488 |  | 16.2337662 |
| P1 | 81.2732095 | 60.795756 | 57.4005305 | 36.2864721 | 44.0318302 |  | R² = 0.8906 |  |  |
| 0D2 | 0.408 | 0.891 | 0.903 | 0.903 | 1.012 | 1.13642796 | y = 4.9462x + 44.379 |  | 22.1238938 |
| P2 | 78.3554377 | 52.7320955 | 52.0954907 | 52.0954907 | 46.3129973 |  | R² = 0.8822 |  |  |
| OD3 | 0.605 | 0.937 | 0.905 | 0.917 | 1.005 | 1.25350229 | y = 3.1408x + 46.063 |  | 20 |
| P3 | 67.9045093 | 50.2917772 | 51.9893899 | 51.3527851 | 46.6843501 |  | R² = 0.8325 |  |  |
| Average value |  |  |  |  |  | 1.31019298 |  |  | 19.4525533 |
| SD |  |  |  |  |  | 0.20798791 |  |  | 2.98298074 |
|  |  |  |  |  |  |  |  |  |  |
|  |  |  |  |  |  |  |  |  |  |
|  |  |  |  |  |  |  |  |  |  |
|  |  |  | Div Aq |  |  | IC 50 |  |  | AAI |
| C | 6.25 | 3.125 | 1.5625 | 0.78125 | 0.35 |  |  |  |  |
| 0D1 | 0.604 | 0.796 | 0.891 | 1.002 | 1.304 | 2.17373429 | y = 5.1078x + 38.897 |  | 11.5207373 |
| P1 | 67.9575597 | 57.7718833 | 52.7320955 | 46.8435013 | 30.8222812 |  | R² = 0.7845 |  |  |
| 0D2 | 0.508 | 0.592 | 0.988 | 1.409 | 1.001 | 2.06798566 | y = 6.5808x + 36.391 |  | 12.1359223 |
| P2 | 73.0503979 | 68.5941645 | 47.5862069 | 25.2519894 | 46.8965517 |  | R² = 0.6697 |  |  |
| OD3 | 0.405 | 0.696 | 0.999 | 1.207 | 1.508 | 2.53443587 | y = 9.0095x + 27.166 |  | 9.88142292 |
| P3 | 78.5145889 | 63.0769231 | 47.0026525 | 35.9681698 | 20 |  | R² = 0.8908 |  |  |
| Average value |  |  |  |  |  | 2.25871861 |  |  | 11.1793609 |
| SD |  |  |  |  |  | 0.24456227 |  |  | 1.16537346 |
|  |  |  |  |  |  |  |  |  |  |
|  |  |  |  |  |  |  |  |  |  |
|  |  |  |  |  |  |  |  |  |  |
|  |  |  |  |  |  |  |  |  |  |
|  |  |  | Vit C |  |  | IC 50 |  |  | AAI |
| C | 6.25 | 3.125 | 1.5625 | 0.78125 | 0.35 |  |  |  |  |
| 0D1 | 0.358 | 0.566 | 0.95 | 0.788 | 1.042 | 0.54572117 | y = 5.7282x + 46.874 |  | 46.2962963 |
| P1 | 81.0079576 | 69.9734748 | 49.602122 | 58.1962865 | 44.7214854 |  | R² = 0.8498 |  |  |
| 0D2 | 0.291 | 0.68 | 0.898 | 1.06 | 1.248 | 1.70788974 | y = 8.0497x + 36.252 |  | 14.7058824 |
| P2 | 84.5623342 | 63.9257294 | 52.3607427 | 43.7665782 | 33.7931034 |  | R² = 0.966 |  |  |
| OD3 | 0.445 | 0.876 | 0.881 | 1.038 | 1.955 | 2.94570582 | y = 9.6235x + 21.652 |  | 8.50340136 |
| P3 | 76.3925729 | 53.5278515 | 53.2625995 | 44.933687 | -3.71352785 |  | R² = 0.6053 |  |  |
| Average value |  |  |  |  |  | 1.73310558 |  |  | 23.1685267 |
| SD |  |  |  |  |  | 1.20019101 |  |  | 20.2679054 |
|  |  |  |  |  |  |  |  |  |  |
|  |  |  |  |  |  |  |  |  |  |
|  | IC 50 Vit C |  |  | AAI Vit C |  |  |  |  |  |
|  | 0.54572117 |  |  | 46.2962963 |  |  |  |  |  |
|  | 1.70788974 |  |  | 14.7058824 |  |  |  |  |  |
|  | 2.94570582 |  |  | 8.50340136 |  |  |  |  |  |

**Legend: C:** Concentration; **OD:** Optical Density; **IC50:** Median inhibitory concentration; **AAI**: Antioxydant Activity Index; **SD:** Standard deviation.

| IC 50 Mufuma EtOH | IC 50 Mububa EtOH | IC 50 Iroko EtOH | IC 50 Div EtOH |
| --- | --- | --- | --- |
| 2.61581599 | 3.412796385 | 1.858211868 | 1.540648679 |
| 1.805292851 | 1.955502887 | 3.165375437 | 1.136427965 |
| 3.548697563 | 1.195978391 | 1.727506354 | 1.253502292 |
|  |  |  |  |
|  |  |  |  |
| IC 50 Mufuma Aq | IC 50 Mububa Aq | IC 50 Iroko Aq | IC 50 Div Aq |
| 2.326437717 | 2.173734289 | 2.326437717 | 2.173734289 |
| 2.969588206 | 2.067985655 | 13.24541866 | 2.067985655 |
| 2.889962843 | 2.124339833 | 2.889962843 | 2.534435873 |

**Legend: IC50:** Median inhibitory concentration; **AAI**: Antioxydant Activity Index.

| IC 50 Mufuma EtOH | IC 50 Mufuma Aq |  | AAI Mufuma EtOH | AAI Mufuma Aq |
| --- | --- | --- | --- | --- |
| 2.61581599 | 2.326437717 |  | 9.578544061 | 10.77586207 |
| 1.805292851 | 2.969588206 |  | 13.88888889 | 8.445945946 |
| 3.548697563 | 2.889962843 |  | 7.062146893 | 8.680555556 |
| F | 6.191739082 |  | F | 7.242935602 |
| Critical value for F (one-sided) | 19 |  | Critical value for F (one-sided) | 19 |
| t statistic | -0.132746943 |  | t statistic | 0.411837968 |
| Critical value of t (two-tailed) | 2.776445105 |  | Critical value of t (two-tailed) | 2.776445105 |
|  |  |  |  |  |
|  |  |  |  |  |
| IC 50 Vit C | IC 50 Mufuma Aq |  | AAI Vit C | AAI Mufuma Aq |
| 0.545721169 | 2.326437717 |  | 46.2962963 | 10.77586207 |
| 1.707889735 | 2.969588206 |  | 14.70588235 | 8.445945946 |
| 2.945705824 | 2.889962843 |  | 8.503401361 | 8.680555556 |
| V = | NS |  | V ≠ | NS |
|  |  |  |  |  |
| IC 50 Vit C | IC 50 Mufuma EtOH | | AAI Vit C | AAI Mufuma EtOH |
| 0.545721169 | 2.61581599 |  | 46.2962963 | 9.578544061 |
| 1.707889735 | 1.805292851 |  | 14.70588235 | 13.88888889 |
| 2.945705824 | 3.548697563 |  | 8.503401361 | 7.062146893 |
| V = | NS |  | V ≠ | NS |
|  |  |  |  |  |
|  |  |  |  |  |
|  |  |  |  |  |
|  |  |  |  |  |
|  |  |  |  |  |
|  |  |  |  |  |
|  |  |  |  |  |
|  |  |  |  |  |
| IC 50 Mububa EtOH | IC 50 Mububa Aq |  | AAI Mububa EtOH | AAI Mububa Aq |
| 3.412796385 | 2.173734289 |  | 7.331378299 | 11.52073733 |
| 1.955502887 | 2.067985655 |  | 12.82051282 | 12.13592233 |
| 1.195978391 | 2.124339833 |  | 21.00840336 | 11.79245283 |
| F | 453.3094399 |  | F | 498.4332644 |
| Critical value for F (one-sided) | 19 |  | Critical value for F (one-sided) | 19 |
| t statistic | 0.101472479 |  | t statistic | 0.478595267 |
| Critical value of t (two-tailed) | 4.30265273 |  | Critical value of t (two-tailed) | 4.30265273 |
|  |  |  |  |  |
|  |  |  |  |  |
| IC 50 Vit C | IC 50Mububa Aq |  | AAI Vit C | AAI Mububa Aq |
| 0.545721169 | 2.173734289 |  | 46.2962963 | 11.52073733 |
| 1.707889735 | 2.067985655 |  | 14.70588235 | 12.13592233 |
| 2.945705824 | 2.124339833 |  | 8.503401361 | 11.79245283 |
| V ≠ | NS |  | V ≠ | NS |
|  |  |  |  |  |
| IC 50 Vit C | IC 50 Mububa EtOH |  | AAI Vit C | AAI Mububa EtOH |
| 0.545721169 | 3.412796385 |  | 46.2962963 | 7.331378299 |
| 1.707889735 | 1.955502887 |  | 14.70588235 | 12.82051282 |
| 2.945705824 | 1.195978391 |  | 8.503401361 | 21.00840336 |
| V = | NS |  | V = | NS |
|  |  |  |  |  |
|  |  |  |  |  |
|  |  |  |  |  |
|  |  |  |  |  |
|  |  |  |  |  |
|  |  |  |  |  |
|  |  |  |  |  |
|  |  |  |  |  |
| IC 50 Iroko EtOH | IC 50 Iroko Aq |  | AAI Iroko EtOH | AAI Iroko Aq |
| 1.858211868 | 2.326437717 |  | 13.51351351 | 10.77586207 |
| 3.165375437 | 13.24541866 |  | 7.911392405 | 1.888217523 |
| 1.727506354 | 2.889962843 |  | 14.53488372 | 8.680555556 |
| F | 0.016726675 |  | F | 0.589096057 |
| Critical value for F (one-sided) | 0.052631579 |  | Critical value for F (one-sided) | 0.052631579 |
| t statistic | -1.090679188 |  | t statistic | 1.440724582 |
| Critical value of t (two-tailed) | 2.776445105 |  | Critical value of t (two-tailed) | 2.776445105 |
|  |  |  |  |  |
|  |  |  |  |  |
| IC 50 Vit C | IC Iroko Aq |  | AAI Vit C | AAI Iroko Aq |
| 0.545721169 | 2.326437717 |  | 46.2962963 | 10.77586207 |
| 1.707889735 | 13.24541866 |  | 14.70588235 | 1.888217523 |
| 2.945705824 | 2.889962843 |  | 8.503401361 | 8.680555556 |
| V = | NS |  | V ≠ | NS |
|  |  |  |  |  |
| IC 50 Vit C | IC 50 Iroko EtOH |  | AAI Vit C | AAI Iroko EtOH |
| 0.545721169 | 1.858211868 |  | 46.2962963 | 13.51351351 |
| 1.707889735 | 3.165375437 |  | 14.70588235 | 7.911392405 |
| 2.945705824 | 1.727506354 |  | 8.503401361 | 14.53488372 |
| V = | NS |  | V ≠ | NS |
|  |  |  |  |  |
|  |  |  |  |  |
|  |  |  |  |  |
|  |  |  |  |  |
|  |  |  |  |  |
|  |  |  |  |  |
|  |  |  |  |  |
|  |  |  |  |  |
| IC 50 Div EtOH | IC 50 Div Aq |  | AAI Div EtOH | AAI Div Aq |
| 1.540648679 | 2.173734289 |  | 16.23376623 | 11.52073733 |
| 1.136427965 | 2.067985655 |  | 22.12389381 | 12.13592233 |
| 1.253502292 | 2.534435873 |  | 20 | 9.881422925 |
| F | 0.723264739 |  | F | 6.551951111 |
| Critical value for F (one-sided) | 0.052631579 |  | Critical value for F (one-sided) | 19 |
| t statistic | -5.117339678 |  | t statistic | 4.474443685 |
| Critical value of t (two-tailed) | 2.776445105 |  | Critical value of t (two-tailed) | 2.776445105 |
|  |  |  |  |  |
|  |  |  |  |  |
| IC 50 Vit C | IC Div Aq |  | AAI Vit C | AAI Div Aq |
| 0.545721169 | 2.173734289 |  | 46.2962963 | 11.52073733 |
| 1.707889735 | 2.067985655 |  | 14.70588235 | 12.13592233 |
| 2.945705824 | 2.534435873 |  | 8.503401361 | 9.881422925 |
| V ≠ | NS |  | V ≠ | NS |
|  |  |  |  |  |
| IC 50 Vit C | IC 50 Div EtOH |  | AAI Vit C | AAI Div EtOH |
| 0.545721169 | 1.540648679 |  | 46.2962963 | 16.23376623 |
| 1.707889735 | 1.136427965 |  | 14.70588235 | 22.12389381 |
| 2.945705824 | 1.253502292 |  | 8.503401361 | 20 |
| V ≠ | NS |  | V ≠ | NS |

**Legend: IC50:** Median inhibitory concentration; **AAI**: Antioxydant Activity Index; **V:** Variance; S: Significant; NS: Not Significant.

Phosphomolybdenum assay

| Mufuma EtOH |  |  | Mufuma Aq |  |  | Iroko EtOH |  |  | Iroko Aq |  |
| --- | --- | --- | --- | --- | --- | --- | --- | --- | --- | --- |
| C | 5 |  | C | 5 |  | C | 5 |  | C | 5 |
| 0D1 | 0.22 |  | 0D1 | 0.282 |  | 0D1 | 0.729 |  | 0D1 | 0.498 |
| 0D2 | 0.342 |  | 0D2 | 0.308 |  | 0D2 | 0.364 |  | 0D2 | 0.86 |
| 0D3 | 0.304 |  | 0D3 | 0.185 |  | 0D3 | 0.521 |  | 0D3 | 0.552 |
| Average value | 0.28866667 |  | Average value | 0.25833333 |  | Average value | 0.538 |  | Average value | 0.63666667 |
| Percentage of inhibition | 84.6861185 |  | Percentage of inhibition | 86.2953139 |  | Percentage of inhibition | 71.4588859 |  | Percentage of inhibition | 66.22458 |
|  |  |  |  |  |  |  |  |  |  |  |
|  |  |  |  |  |  |  |  |  |  |  |
| Muboba Aq |  |  | Muboba EtOH | |  | Div Aq |  |  | Div EtOH |  |
| C | 5 |  | C | 5 |  | C | 5 |  | C | 5 |
| 0D1 | 0.871 |  | 0D1 | 0.228 |  | 0D1 | 0.752 |  | 0D1 | 0.378 |
| 0D2 | 0.775 |  | 0D2 | 0.86 |  | 0D2 | 0.288 |  | 0D2 | 0.478 |
| 0D3 | 0.68 |  | 0D3 | 0.522 |  | 0D3 | 0.364 |  | 0D3 | 0.583 |
| Average value | 0.77533333 |  | Average value | 0.53666667 |  | Average value | 0.468 |  | Average value | 0.47966667 |
| Percentage of inhibition | 58.8682582 |  | Percentage of inhibition | 71.5296198 |  | Percentage of inhibition | 75.1724138 |  | Percentage of inhibition | 74.5534925 |
|  |  |  |  |  |  |  |  |  |  |  |
|  |  |  |  |  |  |  |  |  |  |  |
|  |  | Y=0,005X + 0,015 | |  |  |  |  |  |  |  |
|  |  | R^2^=0,983 |  |  |  |  |  |  |  |  |
|  |  |  |  |  |  |  |  |  |  |  |
|  |  |  |  |  |  |  |  |  |  |  |
| Mufuma EtOH |  |  | Mufuma Aq |  |  | Muboba EtOH | |  | Muboba Aq |  |
| C | 5 |  | C | 5 |  | C | 5 |  | C | 5 |
| 0D1 | 0.22 |  | 0D1 | 0.282 |  | 0D1 | 0.228 |  | 0D1 | 0.871 |
| P1 | 41 |  | P1 | 53.4 |  | P1 | 42.6 |  | P1 | 171.2 |
| 0D2 | 0.342 |  | 0D2 | 0.308 |  | 0D2 | 0.86 |  | 0D2 | 0.775 |
| P2 | 65.4 |  | P2 | 58.6 |  | P2 | 169 |  | P2 | 152 |
| 0D3 | 0.304 |  | 0D3 | 0.185 |  | 0D3 | 0.522 |  | 0D3 | 0.68 |
| P3 | 57.8 |  | P3 | 34 |  | P3 | 101.4 |  | P3 | 133 |
| Average value | 54.7333333 |  | Average value | 48.6666667 |  | Average value | 104.333333 |  | Average value | 152.066667 |
| SD | 12.4857252 |  | SD | 12.9650813 |  | SD | 63.2510342 |  | SD | 19.1000873 |
|  |  |  |  |  |  |  |  |  |  |  |
|  |  |  |  |  |  |  |  |  |  |  |
|  |  |  |  |  |  |  |  |  |  |  |
|  |  |  |  |  |  |  |  |  |  |  |
|  |  |  |  |  |  |  |  |  |  |  |
|  |  |  |  |  |  |  |  |  |  |  |
|  |  |  |  |  |  |  |  |  |  |  |
|  |  |  |  |  |  |  |  |  |  |  |
|  |  |  |  |  |  |  |  |  |  |  |
|  |  |  |  |  |  |  |  |  |  |  |
|  |  |  |  |  |  |  |  |  |  |  |
|  |  |  |  |  |  |  |  |  |  |  |
|  |  |  |  |  |  |  |  |  |  |  |
|  |  |  |  |  |  |  |  |  |  |  |
| Iroko EtOH |  |  | Iroko Aq |  |  | Div EtOH |  |  | Div Aq |  |
| C | 5 |  | C | 5 |  | C | 5 |  | C | 5 |
| 0D1 | 0.729 |  | 0D1 | 0.498 |  | 0D1 | 0.378 |  | 0D1 | 0.752 |
| P1 | 142.8 |  | P1 | 96.6 |  | P1 | 72.6 |  | P1 | 147.4 |
| 0D2 | 0.364 |  | 0D2 | 0.86 |  | 0D2 | 0.478 |  | 0D2 | 0.288 |
| P2 | 69.8 |  | P2 | 169 |  | P2 | 92.6 |  | P2 | 54.6 |
| OD3 | 0.521 |  | OD3 | 0.552 |  | OD3 | 0.583 |  | OD3 | 0.364 |
| P3 | 101.2 |  | P3 | 107.4 |  | P3 | 113.6 |  | P3 | 69.8 |
| Average value | 104.6 |  | Average value | 124.333333 |  | Average value | 92.9333333 |  | Average value | 90.6 |
| SD | 36.6185745 |  | SD | 39.0575644 |  | SD | 20.5020324 |  | SD | 49.7738887 |

**Legend: C:** Concentration; **OD:** Optical Density; **IC50:** Median inhibitory concentration; **AAI**: Antioxydant Activity Index; **SD:** Standard deviation.

| Mufuma EtOH | Mufuma Aq |  | Muboba EtOH | Muboba Aq |
| --- | --- | --- | --- | --- |
| 41 | 53.4 |  | 42.6 | 171.2 |
| 65.4 | 58.6 |  | 169 | 152 |
| 57.8 | 34 |  | 101.4 | 133 |
| V≠ | NS |  | v = | NS |

| Iroko EtOH | Iroko Aq |  | Div EtOH | Div Aq |
| --- | --- | --- | --- | --- |
| 142.8 | 96.6 |  | 72.6 | 147.4 |
| 69.8 | 169 |  | 92.6 | 54.6 |
| 101.2 | 107.4 |  | 113.6 | 69.8 |
| V≠ | NS |  | V≠ | NS |

**Legend: V:** Variance; S: Significant; NS: Not Significant.

Beta-carotene assay

|  |  |  | Iroko Aq |  |  |
| --- | --- | --- | --- | --- | --- |
| C | 1 | 0.8 | 0.6 | 0.4 | 0.2 |
| 0D1 | 3.513 | 3.331 | 3.324 | 3.299 | 3.281 |
| 0D2 | 3.42 | 3.423 | 3.335 | 3.306 | 3.287 |
| OD3 | 3.424 | 3.356 | 3.332 | 3.307 | 3.313 |
| Average value | 3.45233333 | 3.37 | 3.33033333 | 3.304 | 3.29366667 |
| Percentage of inhibition | 7.66693412 | 9.86894892 | 10.9298386 | 11.6341268 | 11.910493 |
|  |  |  |  |  |  |
|  |  |  |  |  |  |
|  |  |  |  |  |  |
| X (mg/mL ) | 1 | 0.8 | 0.6 | 0.4 | 0.2 |
| Y (%) | 7.66693412 | 9.86894892 | 10.9298386 | 11.6341268 | 11.910493 |
|  |  |  |  |  |  |
|  |  |  |  |  |  |
|  |  |  |  |  |  |
|  |  |  |  |  |  |
|  |  |  |  |  |  |
|  |  |  |  |  |  |
|  |  |  | Iroko EtOH |  |  |
| C | 1 | 0.8 | 0.6 | 0.4 | 0.2 |
| 0D1 | 3.216 | 3.162 | 3.124 | 3.107 | 3.035 |
| 0D2 | 3.269 | 3.238 | 3.16 | 3.247 | 3.234 |
| OD3 | 3.266 | 3.153 | 3.209 | 3.196 | 3.169 |
| Average value | 3.25033333 | 3.18433333 | 3.16433333 | 3.18333333 | 3.146 |
| Percentage of inhibition | 13.0694482 | 14.834626 | 15.3695284 | 14.8613711 | 15.8598556 |
|  |  |  |  |  |  |
|  |  |  |  |  |  |
|  |  |  |  |  |  |
| X (mg/mL ) | 1 | 0.8 | 0.6 | 0.4 | 0.2 |
| Y (%) | 13.0694482 | 14.834626 | 15.3695284 | 14.8613711 | 15.8598556 |
|  |  |  |  |  |  |
|  |  |  |  |  |  |
|  |  |  |  |  |  |
|  |  |  |  |  |  |
|  |  |  |  |  |  |
|  |  |  |  |  |  |
|  |  |  | Divevengui Aq | |  |
| C | 1 | 0.8 | 0.6 | 0.4 | 0.2 |
| 0D1 | 3.544 | 3.291 | 3.225 | 3.188 | 3.297 |
| 0D2 | 3.412 | 3.438 | 3.301 | 3.455 | 3.207 |
| OD3 | 3.42 | 3.394 | 3.426 | 3.398 | 3.47 |
| Average value | 3.45866667 | 3.37433333 | 3.31733333 | 3.347 | 3.32466667 |
| Percentage of inhibition | 7.49754836 | 9.7530534 | 11.2775252 | 10.4840867 | 11.0813943 |
|  |  |  |  |  |  |
|  |  |  |  |  |  |
|  |  |  |  |  |  |
| X (mg/mL ) | 1 | 0.8 | 0.6 | 0.4 | 0.2 |
| Y (%) | 7.49754836 | 9.7530534 | 11.2775252 | 10.4840867 | 11.0813943 |
|  |  |  |  |  |  |
|  |  |  |  |  |  |
|  |  |  |  |  |  |
|  |  |  |  |  |  |
|  |  |  |  |  |  |
|  |  |  |  |  |  |
|  |  |  | Divevengui EtOH | |  |
| C | 1 | 0.8 | 0.6 | 0.4 | 0.2 |
| 0D1 | 3.213 | 3.086 | 3.088 | 3.077 | 3.079 |
| 0D2 | 3.229 | 3.136 | 3.148 | 3.112 | 3.119 |
| OD3 | 3.187 | 3.155 | 3.096 | 3.121 | 3.121 |
| Average value | 3.20966667 | 3.12566667 | 3.11066667 | 3.10333333 | 3.10633333 |
| Percentage of inhibition | 14.157083 | 16.403673 | 16.8048498 | 17.0009807 | 16.9207453 |
|  |  |  |  |  |  |
|  |  |  |  |  |  |
|  |  |  |  |  |  |
| X (mg/mL ) | 1 | 0.8 | 0.6 | 0.4 | 0.2 |
| Y (%) | 14.157083 | 16.403673 | 16.8048498 | 17.0009807 | 16.9207453 |

**Legend: C:** Concentration; **OD:** Optical Density.

Correlation

|  | DPPH IC50 ( g/mL) | DPPH AAI | PM TAC (mg AAE/g) | BCB RAA (%) | Total phenolic | Total flavonoid | Total proanthocyanidin | Total tannin | |  |  |  |  |  |  |
| --- | --- | --- | --- | --- | --- | --- | --- | --- | --- | --- | --- | --- | --- | --- | --- |
| 1 | 2.73 | 9.3 | 54.73 | 37.07 | 123.333333 | 204.444444 | 117.777778 | 238.888889 |  |  |  |  |  |  |  |
| 2 | 2.12 | 11.81 | 152.06 | 36.47 | 386.388889 | 109.444444 | 119.722222 | 205 |  |  |  |  |  |  |  |
| 3 | 6.15 | 7.11 | 90.6 | 38.67 | 110.555556 | 138.888889 | 40.8333333 | 191.666667 |  |  |  |  |  |  |  |
| 4 | 6.15 | 7.11 | 124.33 | 34.87 | 219.722222 | 225.833333 | 58.0555556 | 206.944444 |  |  |  |  |  |  |  |
|  |  |  |  |  |  |  |  |  |  |  |  |  |  |  |  |
|  |  |  |  |  |  |  |  |  |  |  |  |  |  |  |  |
|  |  | *DPPH IC50 ( g/mL)* | *DPPH AAI* | *PM TAC (mg AAE/g)* | *BCB RAA (%)* | *Total phenolic* | *Total flavonoid* | *Total proanthocyanidin* | *Total tannin* |  |  |  |  |  |  |
|  | 1 |  |  |  |  |  |  |  |  |  |  |  |  |  |  |
| DPPH IC50 ( g/mL) | 0.852115966 | 1 |  |  |  |  |  |  |  |  |  |  |  |  |  |
| DPPH AAI | -0.650282076 | -0.935739502 | 1 |  |  |  |  |  |  |  |  |  |  |  |  |
| PM TAC (mg AAE/g) | 0.451615472 | -0.053095506 | 0.38246719 | 1 |  |  |  |  |  |  |  |  |  |  |  |
| BCB RAA (%) | -0.361677772 | 0.017939763 | -0.07142857 | -0.47007132 | 1 |  |  |  |  |  |  |  |  |  |  |
| Total phenolic | 0.013522919 | -0.501299658 | 0.74816124 | 0.88769296 | -0.47742236 | 1 |  |  |  |  |  |  |  |  |  |
| Total flavonoid | 0.221405279 | 0.348785546 | -0.56440174 | -0.44271475 | -0.53150894 | -0.48119066 | 1 |  |  |  |  |  |  |  |  |
| Total proanthocyanidin | -0.819868489 | -0.980433857 | 0.88432601 | 0.02006179 | -0.17395896 | 0.47780182 | -0.1660956 | 1 |  |  |  |  |  |  |  |
| Total tannin | -0.703656165 | -0.568803101 | 0.26376802 | -0.58625938 | -0.19986505 | -0.20817646 | 0.51786964 | 0.68300897 | 1 |  |  |  |  |  |  |
|  |  |  |  |  |  |  |  |  |  |  |  |  |  |  |  |
|  |  |  |  |  |  |  |  |  |  |  |  |  |  |  |  |
|  | DPPH IC50 ( g/mL) | DPPH AAI | PM TAC (mg AAE/g) | BCB RAA (%) | Total phenolic | Total flavonoid | Total proanthocyanidin | Total tannin | |  |  |  |  |  |  |
| 1 | 2.73 | 9.3 | 54.73 | 37.07 | 295.277778 | 202.222222 | 208.055556 | 210 |  |  |  |  |  |  |  |
| 2 | 2.12 | 11.81 | 152.06 | 36.47 | 231.666667 | 280 | 136.111111 | 381.666667 |  |  |  |  |  |  |  |
| 3 | 6.15 | 7.11 | 90.6 | 38.67 | 373.888889 | 122.5 | 126.944444 | 230 |  |  |  |  |  |  |  |
| 4 | 6.15 | 7.11 | 124.33 | 34.87 | 323.611111 | 368.611111 | 33.3333333 | 240.555556 |  |  |  |  |  |  |  |
|  |  |  |  |  |  |  |  |  |  |  |  |  |  |  |  |
|  |  |  |  |  |  |  |  |  |  |  |  |  |  |  |  |
|  |  | *DPPH IC50 ( g/mL)* | *DPPH AAI* | *PM TAC (mg AAE/g)* | *BCB RAA (%)* | *Total phenolic* | *Total flavonoid* | *Total proanthocyanidin* | *Total tannin* |  |  |  |  |  |  |
|  | 1 |  |  |  |  |  |  |  |  |  |  |  |  |  |  |
| DPPH IC50 ( g/mL) | 0.852115966 | 1 |  |  |  |  |  |  |  |  |  |  |  |  |  |
| DPPH AAI | -0.650282076 | -0.935739502 | 1 |  |  |  |  |  |  |  |  |  |  |  |  |
| PM TAC (mg AAE/g) | 0.451615472 | -0.053095506 | 0.38246719 | 1 |  |  |  |  |  |  |  |  |  |  |  |
| BCB RAA (%) | -0.361677772 | 0.017939763 | -0.07142857 | -0.47007132 | 1 |  |  |  |  |  |  |  |  |  |  |
| Total phenolic | 0.494442627 | 0.874722343 | -0.93820119 | -0.47975771 | 0.41000671 | 1 |  |  |  |  |  |  |  |  |  |
| Total flavonoid | 0.418476778 | -0.010468451 | 0.11632591 | 0.59720305 | -0.98855364 | -0.44145254 | 1 |  |  |  |  |  |  |  |  |
| Total proanthocyanidin | -0.960273717 | -0.68831711 | 0.4705356 | -0.60200607 | 0.59036064 | -0.25069584 | -0.64949438 | 1 |  |  |  |  |  |  |  |
| Total tannin | -0.098753138 | -0.545541857 | 0.80543458 | 0.83600307 | -0.1936164 | -0.78000391 | 0.3106784 | -0.06528712 | 1 |  |  |  |  |  |  |
|  |  |  |  |  |  |  |  |  |  |  |  |  |  |  |  |
|  |  |  |  |  |  |  |  |  |  |  |  |  |  |  |  |
|  | Total phenolic | Total flavonoid | Total proanthocyanidin | Total tannin | E32 | E40 | E41 | E8 | E4 | E7 | E37 | E50 | E49 | E10 |  |
| 1 | 123.3333333 | 204.4444444 | 117.777778 | 238.888889 | 8.66666667 | 9.33333333 | 8.33333333 | 8.66666667 | 9.33333333 | 13.66666667 | 10.33333333 | 13.33333333 | 8.333333333 | 9.666666667 |  |
| 2 | 386.3888889 | 109.4444444 | 119.722222 | 205 | 7.33333333 | 8.33333333 | 0 | 0 | 0 | 0 | 0 | 0 | 0 | 0 |  |
| 3 | 110.5555556 | 138.8888889 | 40.8333333 | 191.666667 | 0 | 0 | 8.66666667 | 8 | 8 | 8 | 9.666666667 | 8.333333333 | 10.33333333 | 0 |  |
| 4 | 219.7222222 | 225.8333333 | 58.0555556 | 206.944444 | 0 | 0 | 0 | 0 | 0 | 0 | 0 | 0 | 9.333333333 | 8.666666667 |  |
|  |  |  |  |  |  |  |  |  |  |  |  |  |  |  |  |
|  |  |  |  |  |  |  |  |  |  |  |  |  |  |  |  |
|  |  | *Total phenolic* | *Total flavonoid* | *Total proanthocyanidin* | *Total tannin* | *E32* | *E40* | *E41* | *E8* | *E4* | *E7* | *E37* | *E50* | *E49* | *E10* |
|  | 1 |  |  |  |  |  |  |  |  |  |  |  |  |  |  |
| Total phenolic | 0.013522919 | 1 |  |  |  |  |  |  |  |  |  |  |  |  |  |
| Total flavonoid | 0.221405279 | -0.481190663 | 1 |  |  |  |  |  |  |  |  |  |  |  |  |
| Total proanthocyanidin | -0.819868489 | 0.477801815 | -0.1660956 | 1 |  |  |  |  |  |  |  |  |  |  |  |
| Total tannin | -0.703656165 | -0.20817646 | 0.51786964 | 0.68300897 | 1 |  |  |  |  |  |  |  |  |  |  |
| E32 | -0.925291513 | 0.305412738 | -0.18383087 | 0.97566848 | 0.72895245 | 1 |  |  |  |  |  |  |  |  |  |
| E40 | -0.916808416 | 0.338336362 | -0.21128626 | 0.98002409 | 0.70562829 | 0.99929956 | 1 |  |  |  |  |  |  |  |  |
| E41 | -0.429510706 | -0.844960399 | 0.02899582 | -0.15753999 | 0.24145898 | 0.06164106 | 0.03575788 | 1 |  |  |  |  |  |  |  |
| E8 | -0.482219748 | -0.840486831 | 0.07022689 | -0.09228617 | 0.32217677 | 0.1255953 | 0.09839536 | 0.99645468 | 1 |  |  |  |  |  |  |
| E4 | -0.512989176 | -0.83476569 | 0.09537899 | -0.05176182 | 0.37076526 | 0.16455037 | 0.13663522 | 0.99075511 | 0.99865603 | 1 |  |  |  |  |  |
| E7 | -0.638842645 | -0.777511552 | 0.21004706 | 0.14046506 | 0.58549125 | 0.3415335 | 0.31127498 | 0.92792495 | 0.95599654 | 0.9699169 | 1 |  |  |  |  |
| E37 | -0.476498686 | -0.841282673 | 0.06564359 | -0.09961149 | 0.31326909 | 0.11849193 | 0.09142957 | 0.99720168 | 0.99995579 | 0.998124562 | 0.953195769 | 1 |  |  |  |
| E50 | -0.621366443 | -0.789778963 | 0.19262052 | 0.1103962 | 0.55363517 | 0.31470679 | 0.28469946 | 0.94168897 | 0.96665927 | 0.978631499 | 0.999247068 | 0.964208851 | 1 |  |  |
| E49 | 0.36333621 | -0.917059306 | 0.63985647 | -0.70896681 | 0.0185405 | -0.60178352 | -0.63107479 | 0.57326985 | 0.55806959 | 0.546736792 | 0.473607701 | 0.559964842 | 0.487176951 | 1 |  |
| E10 | -0.072963647 | -0.37174138 | 0.94708833 | 0.15436303 | 0.75662038 | 0.14102641 | 0.11353515 | 0.0337548 | 0.09628683 | 0.134469163 | 0.308917583 | 0.089332085 | 0.282363024 | 0.438893321 | 1 |
|  |  |  |  |  |  |  |  |  |  |  |  |  |  |  |  |
|  |  |  |  |  |  |  |  |  |  |  |  |  |  |  |  |
|  | Total phenolic | Total flavonoid | Total proanthocyanidin | Total tannin | E32 | E40 | E41 | E8 | E4 | E7 | E37 | E50 | E49 | E10 |  |
| 1 | 123.3333333 | 204.4444444 | 117.777778 | 238.888889 | 9 | 9.66666667 | 9.66666667 | 11.6666667 | 10.3333333 | 11.66666667 | 10.33333333 | 9.333333333 | 8.333333333 | 10.33333333 |  |
| 2 | 386.3888889 | 109.4444444 | 119.722222 | 205 | 9 | 9.33333333 | 17.6666667 | 8 | 14.6666667 | 8 | 8 | 9 | 9.333333333 | 8 |  |
| 3 | 110.5555556 | 138.8888889 | 40.8333333 | 191.666667 | 9 | 10.3333333 | 10.6666667 | 7.33333333 | 9.66666667 | 7.666666667 | 0 | 0 | 8.333333333 | 8 |  |
| 4 | 219.7222222 | 225.8333333 | 58.0555556 | 206.944444 | 8.66666667 | 0 | 8.33333333 | 8.66666667 | 9.33333333 | 13.66666667 | 10.33333333 | 13.33333333 | 8.666666667 | 9.666666667 |  |
|  |  |  |  |  |  |  |  |  |  |  |  |  |  |  |  |
|  |  |  |  |  |  |  |  |  |  |  |  |  |  |  |  |
|  |  | *Total phenolic* | *Total flavonoid* | *Total proanthocyanidin* | *Total tannin* | *E32* | *E40* | *E41* | *E8* | *E4* | *E7* | *E37* | *E50* | *E49* | *E10* |
|  | 1 |  |  |  |  |  |  |  |  |  |  |  |  |  |  |
| Total phenolic | 0.013522919 | 1 |  |  |  |  |  |  |  |  |  |  |  |  |  |
| Total flavonoid | 0.221405279 | -0.481190663 | 1 |  |  |  |  |  |  |  |  |  |  |  |  |
| Total proanthocyanidin | -0.819868489 | 0.477801815 | -0.1660956 | 1 |  |  |  |  |  |  |  |  |  |  |  |
| Total tannin | -0.703656165 | -0.20817646 | 0.51786964 | 0.68300897 | 1 |  |  |  |  |  |  |  |  |  |  |
| E32 | -0.774596669 | -0.050919205 | -0.6861688 | 0.42725202 | 0.12250906 | 1 |  |  |  |  |  |  |  |  |  |
| E40 | -0.736728736 | -0.116923623 | -0.67647927 | 0.35280207 | 0.08417072 | 0.99640379 | 1 |  |  |  |  |  |  |  |  |
| E41 | -0.340822534 | 0.816220309 | -0.86082378 | 0.54428489 | -0.2358646 | 0.52 | 0.4692156 | 1 |  |  |  |  |  |  |  |
| E8 | -0.652550983 | -0.335479116 | 0.59244502 | 0.57897255 | 0.9905003 | 0.08714893 | 0.05920604 | -0.35905361 | 1 |  |  |  |  |  |  |
| E4 | -0.416526081 | 0.86699758 | -0.73042556 | 0.69230565 | -0.05406898 | 0.44811071 | 0.38358342 | 0.97508892 | -0.18745138 | 1 |  |  |  |  |  |
| E7 | 0.251335232 | -0.206509568 | 0.95709058 | -0.02720439 | 0.5094368 | -0.78255106 | -0.79356809 | -0.69093532 | 0.55057806 | -0.528570145 | 1 |  |  |  |  |
| E37 | -0.21065603 | 0.329032939 | 0.57095225 | 0.60893219 | 0.70955874 | -0.43059663 | -0.49422647 | -0.07705413 | 0.65769318 | 0.143192894 | 0.746165129 | 1 |  |  |  |
| E50 | 0.068754393 | 0.422222993 | 0.57665644 | 0.41904677 | 0.48482039 | -0.64105557 | -0.69949847 | -0.09191751 | 0.43576501 | 0.108718326 | 0.783265255 | 0.956851567 | 1 |  |  |
| E49 | -9.72951E-16 | 0.997861592 | -0.53737252 | 0.46887907 | -0.24190177 | 1.2659E-15 | -0.06405126 | 0.84852814 | -0.36974161 | 0.887213951 | -0.269925443 | 0.272427502 | 0.362635792 | 1 |  |
| E10 | -0.217642875 | -0.410612665 | 0.89987723 | 0.24329617 | 0.83911921 | -0.37463432 | -0.38813371 | -0.6668491 | 0.88152252 | -0.491042141 | 0.868785711 | 0.725923247 | 0.615182714 | -0.463586325 | 1 |

| **Legend: DPPH:** 2,2-diphényl 1-picrylhydrazyle**; IC50:** Median inhibitory concentration; **AAI**: Antioxydant Activity Index; PM TAC: Phosphomolybdenum Total Antioxydant Capacity; BCB RAA : Bêta-carotene Bleaching Relative Antioxidant Activity |
| --- |

**Bibliography**

1. Oke-Altuntas, F., et al., *Bioactivity evaluation of cudraxanthone I, neocyclomorusin and (9βh)-3β-acetoxylanosta-7, 24-diene isolated from Milicia excelsa Welw. CC Berg (Moraceae).* Medicinal Chemistry Research, 2016. **25**(10): p. 2250-2257.

2. Abiola, A.L., et al., *Spectroscopic analysis and anti-inflammatory effects of Milicia excelsa (Moraceae) leaf and fractions.* GSC Biological and Pharmaceutical Sciences, 2019. **6**(3): p. -.

3. Ayepola, O., A. Samson, and O. Onile-Ere, *In vitro Antioxidant and Anti-staphylococcal Activity of Bixa orellana Linn. and Milicia excelsa Welw.* Journal of Complementary and Alternative Medical Research, 2018. **5**(4): p. 1-6.

4. Adebayo, M.A., et al., *Evaluation of anti-diarrheal activity of methanol root bark extract of Milicia excelsa (Welw) C. C Berg (Moraceae) in rats.* Drug Research, 2019. **69**(08): p. 439-444.

5. Abiola, A.L., et al., *Anti-amnesic and cognitive enhancing effects of ethanol leaf extract of Milicia excelsa (Moraceae) in mice.* International Journal of Neuroscience and Behavioral Science, 2019. **7**(1): p. 1-11.

6. Biwôlé, J.J.E., et al., *Iroko wood (Milicia excelsa CC berg), a good candidate for high-speed rotation-induced wood dowel welding: An assessment of its welding potential and the water resistance of its welded joints.* International Journal of Adhesion and Adhesives, 2023. **123**: p. 103360.

7. Simsek, E., D. Kilic, and O. Caliskan, *Phenotypic variation of fig genotypes (Ficus carica L.) in the eastern Mediterranean of Turkey.* Genetika, 2020. **52**(3): p. 957-972.

8. Murugesu, S., J. Selamat, and V. Perumal, *Phytochemistry, pharmacological properties, and recent applications of Ficus benghalensis and Ficus religiosa.* Plants, 2021. **10**(12): p. 2749.

9. Tijani, R.O., et al., *Myrianthus arboreus P. Beauv improves insulin sensitivity in high fat diet-induced obese mice by reducing inflammatory pathways activation.* Journal of Ethnopharmacology, 2022. **282**: p. 114651.

10. Kasangana, P.B., et al., *Root bark extracts of Myrianthus arboreus P. Beauv.(Cecropiaceae) exhibit anti-diabetic potential by modulating hepatocyte glucose homeostasis.* Journal of ethnopharmacology, 2018. **211**: p. 117-125.

11. Kasangana, P.B., P.S. Haddad, and T. Stevanovic, *Study of polyphenol content and antioxidant capacity of Myrianthus arboreus (Cecropiaceae) root bark extracts.* Antioxidants, 2015. **4**(2): p. 410-426.

12. Seukep, J.A., B. Ngadjui, and V. Kuete, *Antibacterial activities of Fagara macrophylla, Canarium schweinfurthii, Myrianthus arboreus, Dischistocalyx grandifolius and Tragia benthamii against multi-drug resistant Gram-negative bacteria.* Springerplus, 2015. **4**: p. 1-6.

13. Agu, A., et al., *Protective effect of ethanolic leaf extract of myrianthus arboreus on indomethacin induced gastric ulcer in adult male wistar rats.* Journal of medical pharmaceutical and allied sciences, 2023. **12**(2).

14. Bemmo, U.L.K., et al., *Contribution to the valorization of Myrianthus arboreus fruits pulp from Cameroon: Physico-chemical characterization and Nutritional value.* Measurement: Food, 2023. **10**: p. 100083.

15. García-Pérez, M.-E., P.-B. Kasangana, and T. Stevanovic, *Bioactive Molecules from Myrianthus arboreus, Acer rubrum, and Picea mariana Forest Resources.* Molecules, 2023. **28**(5): p. 2045.

16. Afzal, S., et al., *A Comparative Analysis of Antimicrobial, Antibiofilm and Antioxidant Activity of Silver Nanoparticles Synthesized from Erythrina Suberosa Roxb. and Ceiba Pentandra.* Journal of Oleo Science, 2022. **71**(4): p. 523-533.

17. Njokuocha, R.C. and A. Ewinike, *Antibacterial and phytochemical properties of crude leaf extract of Moringa oleifera Lam., Pterocarpus santalinoides L’Herit DC and Ceiba pentandra L. on some clinical bacteria isolates in Nigeria.* Journal of Complementary and Alternative Medical Research, 2020. **10**(4): p. 1-15.

18. Bhavani, R., E. Bhuvaneswari, and S. Rajeshkumar, *Antibacterial and Antioxidant activity of Ethanolic extract of Ceiba pentandra leaves and its Phytochemicals Analysis using GC-MS.* Research Journal of Pharmacy and Technology, 2016. **9**(11): p. 1922-1926.

19. Silué, G.N.A., et al., *Anti-inflammatory and antioxidant activities assessment of an aqueous extract of Ceiba pentandra (L.) Gaertn (Malvaceae).* RPS Pharmacy and Pharmacology Reports, 2023. **2**(4): p. rqad037.
