# Supplementary material for: Antibacterial and antioxidant activities of plants consumed by western lowland gorilla (Gorilla gorilla gorilla) in Gabon
Source: PLoS One. 2024 Sep 11;19(9):e0306957. doi: 10.1371/journal.pone.0306957 (PMC11389915; doi:10.1371/journal.pone.0306957)
Supplement: Attachment — Submitted filename: PONE-D-23-26722.pdf [file pone.0306957.s003.pdf]

PLOS ONE

Antibacterial Activities of Four Antioxidant Plants Consumed by Western Lowland Gorilla (Gorilla gorilla gorilla) living in Moukalaba-Doudou National Park (MDNP) infected by MDR E. coli (DECs).

--Manuscript Draft--

**Manuscript Number:**	PONE-D-23-26722
**Article Type:**	Research Article
**Full Title:**	Antibacterial Activities of Four Antioxidant Plants Consumed by Western Lowland Gorilla (Gorilla gorilla gorilla) living in Moukalaba-Doudou National Park (MDNP) infected by MDR E. coli (DECs).
**Short Title:**	Antibacterial Activities of Four Antioxidant Plants Consumed by Western Lowland Gorilla.
**Corresponding Author:**	Leresche Even Doneilly OYABA YINDA<br>Centre international de Recherches Médicales de Franceville: Centre International de Recherches Medicales de Franceville<br>Franceville, GABON
**Keywords:**	zoopharmacognosy; Ceiba pentandra; Myrianthus arboreus; Ficus ssp; Milicia excelsa; antioxidant activity; antimicrobial activity
**Abstract:**	Zoopharmacognosy is the study of wild animals self-medicating by using plants as remedies. The aim of this study was to determine the antioxidant and antibacterial activity of Ceiba pentandra, Myrianthus arboreus, Ficus ssp. and Milicia excelsa bark crude extracts (BCE) and to characterize their phytochemical compounds. DPPH (2,2-Diphenyl-1-Picrylhydrazyl), phosphomolybdenum complex and β-carotene bleaching methods were used to assess antioxidant activity. Antimicrobial susceptibility testing was performed using the diffusion method, while minimum inhibitory concentration (MIC) and minimum bactericidal concentration (MBC) were assessed using the microdilution method. The highest level of total phenolics was found in Myrianthus arboreus aqueous extract [385.83 ± 3.99 mg [gallic acid equivalent (GAE)/g]. Total flavonoid (134.46 ± 3.39) mg quercetin equivalent (QE)/100 g of extract] were highest in Milicia excelsa, tannin [(272.44 ± 3.39) mg tannic acid equivalent (TAE)/100 g of extract] in Myrianthus arboreus and proanthocyanidin [(404.33 ± 3.39) mg apple procyanidins equivalent (APE)/100 g of extract] in Ceiba pentandra. Ficus ssp. (IC50 1.34 ±3.36 μg/mL; AAI 18.57 ± 0.203) ethanolic BCE and Milicia excelsa (IC50 2.07 ± 3.37 μg/mL; AAI 12.03 ± 0.711) showed the strongest antioxidant activity. Myrianthus arboreus ethanolic BCE (73.25 ± 5.29) and Milicia excelsa aqueous BCE (38.67 ± 0.27) showed the strongest percentage of total antioxidant capacity (TAC). Ceiba pentandra ethanolic BCE (152.06 ± 19.11 mg AAE/g) and Ficus ssp aqueous BCE (124.33 ± 39.05 mg AAE/g) showed strongest relative antioxidant activity (RAA). The plant BCE showed antimicrobial activity against multidrug resistant (MDR) E. coli (DECs) isolates, with MICs varying from 1.56 to 50 mg/mL and inhibition diameters ranging from 7.34 ± 0.57 to 13.67 ± 0.57mm. Several families of compounds were found, including total phenolic compounds, flavonoids, tannins and proanthocyanidins were found in the plant BCEs. The plant BCEs showed antioxidant activities with free radical scavenging and antimicrobial activities against 10 MDR E. coli (DECs) isolates, and could be a promising novel source for new drug discovery.
**Order of Authors:**	Leresche Even Doneilly OYABA YINDA
	Leresche Even Doneilly OYABA YINDA1
	Richard ONANGA
	Cédric SIMA OBIANG2
	Herman BEGOUABE
	Etienne François AKOMO-OKOUE
	Roland MITOLA
	Joseph-Privat ONDO
	Guy-Roger NDONG ATOME2

Powered by Editorial Manager® and ProduXion Manager® from Aries Systems Corporation

	Louis-Clément OBAME ENGONGA2
	IBRAHIM5
	Joanna M. SETCHELL
	Sylvain GODREUIL
**Additional Information:**	
**Question**	**Response**
**Financial Disclosure**<br><br>Enter a financial disclosure statement that describes the sources of funding for the work included in this submission. Review the submission guidelines for detailed requirements. View published research articles from *PLOS ONE* for specific examples.<br><br>This statement is required for submission and **will appear in the published article** if the submission is accepted. Please make sure it is accurate.<br><br>**Unfunded studies**<br>Enter: *The author(s) received no specific funding for this work.*<br><br>**Funded studies**<br>Enter a statement with the following details:<br>• Initials of the authors who received each award<br>• Grant numbers awarded to each author<br>• The full name of each funder<br>• URL of each funder website<br>• Did the sponsors or funders play any role in the study design, data collection and analysis, decision to publish, or preparation of the manuscript?<br>• **NO** - Include this sentence at the end of your statement: *The funders had no role in study design, data collection and analysis, decision to publish, or preparation of the manuscript.*<br>• **YES** - Specify the role(s) played.<br><br>* typeset	Non.<br>Les bailleurs de fonds n'ont joué aucun rôle dans la conception de l'étude, la collecte et l'analyse des données, la décision de publier ou la préparation du manuscrit.
**Competing Interests**<br><br>Use the instructions below to enter a competing interest statement for this submission. On behalf of all authors, disclose any competing interests that	Les auteurs ont déclaré qu'il n'existe aucun intérêt concurrent.

could be perceived to bias this work—acknowledging all financial support and any other relevant financial or non-financial competing interests.<br><br>This statement is required for submission and **will appear in the published article** if the submission is accepted. Please make sure it is accurate and that any funding sources listed in your Funding Information later in the submission form are also declared in your Financial Disclosure statement.<br><br>View published research articles from *PLOS ONE* for specific examples.<br><br>**NO authors have competing interests**<br><br>Enter: *The authors have declared that no competing interests exist.*<br><br>**Authors with competing interests**<br><br>Enter competing interest details beginning with this statement:<br><br>*I have read the journal's policy and the authors of this manuscript have the following competing interests: [insert competing interests here]*<br><br>* typeset	
**Ethics Statement**<br><br>Enter an ethics statement for this submission. This statement is required if the study involved:<br><br>• Human participants<br>• Human specimens or tissue<br>• Vertebrate animals or cephalopods<br>• Vertebrate embryos or tissues<br>• Field research<br><br>Write "N/A" if the submission does not require an ethics statement.	N/A

Powered by Editorial Manager® and ProduXion Manager® from Aries Systems Corporation

General guidance is provided below. Consult the submission guidelines for detailed instructions. **Make sure that all information entered here is included in the Methods section of the manuscript.**<br><br>**Format for specific study types**<br><br>**Human Subject Research (involving human participants and/or tissue)**<br>• Give the name of the institutional review board or ethics committee that approved the study<br>• Include the approval number and/or a statement indicating approval of this research<br>• Indicate the form of consent obtained (written/oral) or the reason that consent was not obtained (e.g. the data were analyzed anonymously)<br><br>**Animal Research (involving vertebrate animals, embryos or tissues)**<br>• Provide the name of the Institutional Animal Care and Use Committee (IACUC) or other relevant ethics board that reviewed the study protocol, and indicate whether they approved this research or granted a formal waiver of ethical approval<br>• Include an approval number if one was obtained<br>• If the study involved *non-human primates*, add *additional details* about animal welfare and steps taken to ameliorate suffering<br>• If anesthesia, euthanasia, or any kind of animal sacrifice is part of the study, include briefly which substances and/or methods were applied<br><br>**Field Research**<br><br>Include the following details if this study involves the collection of plant, animal, or other materials from a natural setting:<br>• Field permit number<br>• Name of the institution or relevant body that granted permission	
**Data Availability**<br><br>Authors are required to make all data	Yes - all data are fully available without restriction

Powered by Editorial Manager® and ProduXion Manager® from Aries Systems Corporation

underlying the findings described fully available, without restriction, and from the time of publication. PLOS allows rare exceptions to address legal and ethical concerns. See the PLOS Data Policy and FAQ for detailed information.<br><br>A Data Availability Statement describing where the data can be found is required at submission. Your answers to this question constitute the Data Availability Statement and **will be published in the article**, if accepted.<br><br>**Important:** Stating 'data available on request from the author' is not sufficient. If your data are only available upon request, select 'No' for the first question and explain your exceptional situation in the text box.<br><br>Do the authors confirm that all data underlying the findings described in their manuscript are fully available without restriction?	
**Describe where the data may be found in full sentences. If you are copying our sample text, replace any instances of XXX with the appropriate details.**<br><br>• If the data are **held or will be held in a public repository**, include URLs, accession numbers or DOIs. If this information will only be available after acceptance, indicate this by ticking the box below. For example: *All XXX files are available from the XXX database (accession number(s) XXX, XXX.*).<br>• If the data are all contained **within the manuscript and/or Supporting Information files**, enter the following: *All relevant data are within the manuscript and its Supporting Information files.*<br>• If neither of these applies but you are able to provide **details of access elsewhere**, with or without limitations, please do so. For example:<br><br>*Data cannot be shared publicly because of [XXX]. Data are available from the*	Toutes les données pertinentes se trouvent dans le manuscrit et ses fichiers d'informations complémentaires.

XXX Institutional Data Access / Ethics Committee (contact via XXX) for researchers who meet the criteria for access to confidential data.<br><br>*The data underlying the results presented in the study are available from (include the name of the third party and contact information or URL).*<br>• This text is appropriate if the data are owned by a third party and authors do not have permission to share the data.<br><br>* typeset	
Additional data availability information:	Tick here if the URLs/accession numbers/DOIs will be available only after acceptance of the manuscript for publication so that we can ensure their inclusion before publication.

Manuscrit Click here to access/download;Manuscript;PHYTOC~1.DOC

Phytochemical Screening, Antioxidant and Antibacterial Activities of Four Plants Consumed by Western Lowland Gorilla (*Gorilla gorilla gorilla*) ~~living in Moukalaba-Doudou National Park (MDNP).~~

Leresche Even Doneilly OYABA YINDA[1], Richard ONANGA[1], Cédric SIMA OBIANG[2], Herman BEGOUABE[2], Etienne François AKOMO-OKOUE[3], Roland MITOLA[4], Joseph-Privat ONDO[2], Guy-Roger NDONG ATOME[2], Louis-Clément OBAME ENGONGA[2], IBRAHIM[5], Joanna M. SETCHELL[5] and Sylvain GODREUIL[6]

[1]Laboratioire de Bactériologie, Centre Interdisciplinaire de Recherches Médicales de Franceville (CIRMF), B.P 769, Franceville, Gabon

[2]Laboratoire de Recherches en Biochimie (LAREBIO), Université des Sciences et Techniques de Masuku (USTM), B.P 943 Franceville, Gabon

[3] Laboratoire de Microbiologie, Institut de Recherches en Ecologie Tropicale (IRET), B.P 13354, Libreville, Gabon

[4]Laboratoire de Physiologie Végétale et Toxicologie, Université des Sciences et Techniques de Masuku (USTM), B.P 943 Franceville, Gabon

[5] Department of Anthropology, Université de Durham

[6] Laboratoire de Bactériologie, CHU de Montpellier, UMR MIVEGEC (IRD, CNRS, Université de Montpellier), 34295 Montpellier, France

Corresponding author

Leresche Even Doneilly OYABA YINDA PhD Student

P.O. Box 769, Franceville, Gabon

Tel: +241 66 92 65 68/ 74 73 43 57

Email: oyabaeven@gmail.com

Main text word count: 5301 words

Abstract

Zoopharmacognosy is the study of wild animals self-medicating by using plants as remedies. The aim of this study was to determine the antioxidant and antibacterial activity of *Ceiba pentandra, Myrianthus arboreus*, *Ficus* ssp. and *Milicia excelsa* bark crude extracts (BCE) and to characterize their phytochemical compounds. DPPH (2,2-Diphenyl-1-Picrylhydrazyl), phosphomolybdenum complex and β-carotene bleaching methods were used to assess antioxidant activity. Antimicrobial susceptibility testing was performed using the diffusion method, while minimum inhibitory concentration (MIC) and minimum bactericidal concentration (MBC) were assessed using the microdilution method. The highest level of total phenolics was found in *Myrianthus arboreus* aqueous extract [385.83 ± 3.99 mg [gallic acid equivalent (GAE)/g]. Total flavonoid (134.46 ± 3.39) mg quercetin equivalent (QE)/100 g of extract] were highest in *Milicia excelsa*, tannin [(272.44 ± 3.39) mg tannic acid equivalent (TAE)/100 g of extract] in *Myrianthus arboreus* and proanthocyanidin [(404.33 ± 3.39) mg apple procyanidins equivalent (APE)/100 g of extract] in *Ceiba pentandra*. *Ficus* ssp. (IC_{50} 1.34 ±3.36 µg/mL; AAI 18.57 ± 0.203) ethanolic BCE and *Milicia excelsa* (IC_{50} 2.07 ± 3.37 µg/mL; AAI 12.03 ± 0.711) showed the strongest antioxidant activity. *Myrianthus arboreus* ethanolic BCE (73.25 ± 5.29) and *Milicia excelsa* aqueous BCE (38.67 ± 0.27) showed the strongest percentage of total antioxidant capacity (TAC). *Ceiba pentandra* ethanolic BCE (152.06 ± 19.11 mg AAE/g) and *Ficus* ssp aqueous BCE (124.33 ± 39.05 mg AAE/g) showed strongest relative antioxidant activity (RAA). The plant BCE showed antimicrobial activity against multidrug resistant (MDR) *E. coli* (DECs) isolates, with MICs varying from 1.56 to 50 mg/mL and inhibition diameters ranging from 7.34 ± 0.57 to 13.67 ± 0.57mm. Several families of compounds were found, including total phenolic compounds, flavonoids, tannins and proanthocyanidins were found in the plant BCEs. The plant BCEs showed antioxidant activities with free radical scavenging and antimicrobial activities against 10 MDR *E. coli* (DECs) isolates, and could be a promising novel source for new drug discovery.

Keywords: zoopharmacognosy, *Ceiba pentandra, Myrianthus arboreus, Ficus ssp, Milicia excelsa*, antioxidant activity, antimicrobial activity.

1. Introduction

Natural medicines from plants have been used to enhance human and veterinary health since time immemorial, as revealed in ancient tales, scriptures and other historical literature. This practice is experiencing a resurgence [1, 2]. The World Health Organization (WHO) estimates that approximately 80% of the world's population uses medicinal plants (MPs) for their health and care needs [3]. Recent studies have focused on the potential to develop antioxidant and antimicrobial drugs from plants [4-6]. These antioxidants, for example, reduce chronic illness, including neurodegenerative disorders (Alzheimer’s disease, Parkinson’s disease), cancers, cardiovascular diseases, retinopathy, atherosclerosis, aging, neurodegenerative and dermatological diseases [7, 8]. Antimicrobial agents are also used as antibiotics to control infections in the human body, but can cause many side effects, especially by increasing reactive oxygen species (ROS) [9]. ROS are very dangerous to human health and well-being and play a role in producing cancer [10]; further, they are aggravating factors for the emergence of various other metabolic diseases [11]. Finally, the use of these antimicrobial agents as antibiotic drugs can lead to resistance selection pressure in microorganisms such as bacteria [12].

Antimicrobial resistance is considered by the WHO to be one of the world’s three greatest threats to human health because of the extensive spread of antibiotic-resistant bacteria (ARB) and antibiotic resistance genes (ARGs) [13]. Infectious diseases caused by multidrug-resistant (MDR) bacteria affect millions of people worldwide [14]. Furthermore, many zoonoses caused by pathogenic microorganisms such as enterobacteria (e.g.; MDR *Escherichia coli*), have been a human public health problem for decades [15]. *E. coli* is a Gram-negative bacteria and gut commensal in animals, including non-human primates (NHPs) [16]. The close phylogenetic relationship between humans and other hominids, combined with a rapidly expanding human–animal interface, enables pathogen transmission across species, leading to morbidity and mortality in great ape populations throughout the world [17, 18]. Several studies have shown that wildlife [19, 20], including wild primates such as western lowland gorillas (*Gorilla gorilla gorilla*), could transmit this kind of pathogens to humans [21, 22], or that humans could transmit the pathogens to wildlife [23, 24]. This situation has great potential for the discovery of new antimicrobial agents [25, 26]. Many recent studies show that natural products from medicinal plants or plants consumed by animals continue to play a role in drug discovery and development [27, 28].

Zoopharmacognosy is the multidisciplinary science that studies the physical, chemical, biochemical, and biological properties of natural drugs and drug substances from plants consumed by animals, with the aim of discovering new drugs [2, 29]. Most great apes, including western lowland gorillas (*Gorilla gorilla gorilla*) have a predominantly frugivorous diet [30, 31]. However, bark is the main fallback food for gorillas [32]. These feeding practices appear to be beneficial to the well-being and health of these animals but also to those of humans [33]. The use of great ape pharmacopoeia or zoopharmacognosy is a very promising strategy for management of human diseases because of the phylogenetic proximity of humans and great apes [34]. Several studies have shown that plants from the diet of great apes, including western lowland gorilla, are also used as MPs by healers in traditional African medicine [35, 36].

Gabon, with its exceptional biodiversity, constitutes a vast reservoir of unexplored potential active biomolecules [37]. As the part of valuation of Gabonese flora and based on the "One Health" concept [38], that promotes interdisciplinary and intersectorial collaborations at human-animal-environment interface, this study aims to evaluate the chemical composition (secondary metabolites families), antioxidant and antimicrobial activities of four plant species consumed by gorillas living in Moukalaba-Doudou National Park (MDNP) to control microbial infections within their communities and, used as traditional MPs by healers in Gabon. *Ceiba pentandra* [39], *Myrianthus arboreus* [40], *Ficus* ssp [41] and *Milicia excelsa* [42] are the four plants selected for this study on the basis of ethnobotanical and ethnopharmacological surveys carried out among local populations.

2. Material and Methods

2.1. Study Area and Field Research Authorization

Sampling of the bark of four plants consumed by western lowland gorillas was carried out under field research authorization N° 003/20/DG/JBLD/N° 306, from August 1 to 11, 2022 in MDNP, during the daily monitoring of gorillas by observing plants items they consumed in their natural environment during this period.

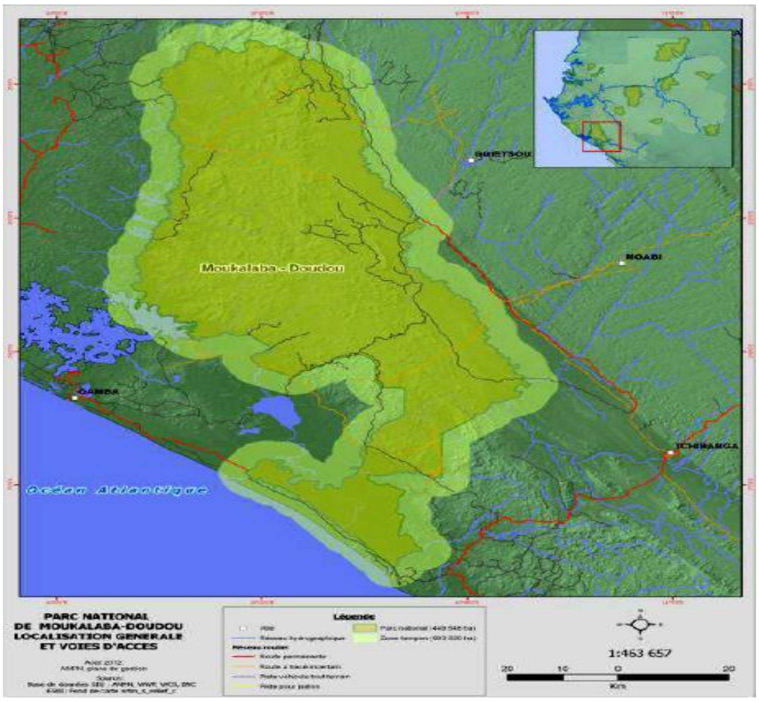

Figure 1: Location of the study area (https://association-program.com/le-parc-national-de-moukalaba-doudou-pnmd/) [31].

2.2. Data and sample collection

A survey was carried out among 27 inhabitants of the village of Doussala, including a number of traditional healers and herbalists, both men and women, known to the local peoples as previously described by Obiang et al. [43]. Fresh bark plant samples collected were identified by a team of botanists leading by Prof. Brama Ibrahim from the Department of Biology, Faculty of Sciences of the University of Science and Technology of Masuku (USTM) in Franceville (Gabon). An herbarium reference specimen of Ceiba pentandra (BRLU/4618), *Myrianthus arboreus* (BRLU-LBV/8324), *Ficus* ssp (USTM/#443) and *Milicia excelsa* (USTM/739) has been deposited at the herbarium of the same institute. The choice of the bark of the selected plants was made based on local traditional medicinal use and the fact that gorillas also consumed them, as recommended by Wubetu et al. [44].

Therapeutic indications

Descriptive statistical methods were used to analyze the ethnopharmacological survey data and various quantitative indices, including use value (UV) and relative frequency of citation (RFC). Data were reported in proportions and percentages [43].

Use Value (UV) and Relative Frequency of Citation (RFC) were calculated according to the following formula:

$UV=U/n$ and $RFC = FC / N$ ($0 < RFC < 1$).

2.3. Treatment of Plant Material

2.3.1. Extraction

Factors such as the solvent kind, time of extraction, temperature and extraction methods can affect extraction yields [45]. From 100 g of bark powder of each selected plant, an extraction by maceration under agitation was carried out for 72 h with 2 L of each solvent (ethanol 99.8% and water). The filtrates were then evaporated on a rotary evaporator ((BÜCHI, Labortechnik, Switzerland) for the ethanolic extracts and freeze-dried for the aqueous extracts were filtered using Whatman N°1 filter paper, then concentrated to obtain the crude extracts used for analysis (Figure 1).

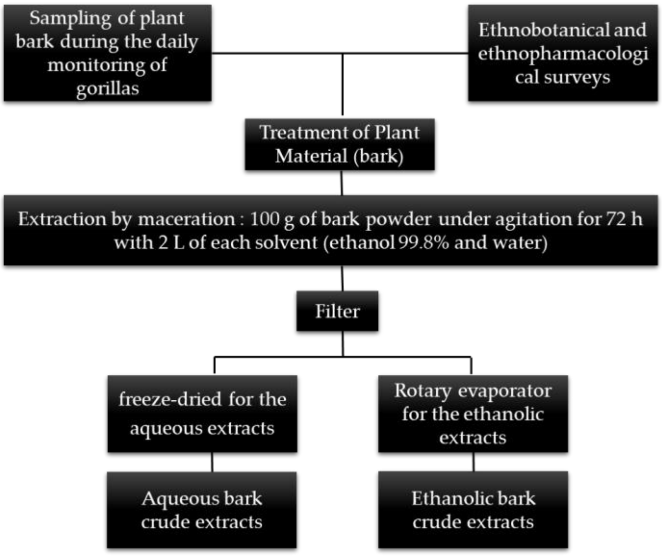

Figure 2: Sequential extraction procedure of the four plant selected bark crude extracts.

2.4. Preliminary Phytochemical Screening

Each plant BCE was tested for the presence of flavonoids, coumarins, tannins, total phenolics, catechin tannins, gallic tannins, cyanidins, alkaloids, oses or holosides, saponosides, sterols or triterpenoids, anthracenosides, cardiotonic heterosides and reducing sugar as described previously [46, 47].

2.5. Quantitative phytochemical analysis

2.5.1. Total phenol content

To determine the total phenol content, the Folin-Ciocalteu method was used [48]. Absorbance was measured at 735 nm. Phenolic compounds were expressed as mg gallic acid equivalents (GAE)/g of extract.

2.5.2. Total flavonoid content

Aluminum trichloride method was used to determine the flavonoid content and absorbance was measured at 435 nm. Flavonoid content was expressed in quercetin equivalent (QE) [49].

2.5.3. Tannin content

Tannin content was determined using the method described previously by Sima-Obiang et al. [50]. Absorbance was measured at 525 nm and tannic acid was used as a standard. Tannin contents were expressed in mg of tannic acid equivalent (TAE)/g of dry extract.

2.5.4. Proanthocyanidin content

Proanthocyanidins were determined using the HCl-Butanol method [51]. Absorbance was read at 550 nm and apple procyanidin was applied as standard. Proanthocyanidin levels were expressed in apple procyanidins equivalent (APE)/g of dry extract.

~~*2.6. Bioactive Properties of Bark Crude Extracts of Selected Species Consumed by Western Lowland Gorilla*~~

2.6.1. Antioxidant Activity of Bark Crude Extracts of Selected Species Consumed by Western Lowland Gorilla

2.6.1.2. Scavenging 2,2-Diphenyl-1-Picrylhydrazyl (DPPH) Radical Capacity

The method described by Scherer and Godoy [52], based on the DPPH (2, 2-diphenyl-1-picrylhydrazyl) radical test, was used to determine the Antioxidant Activity Index (AAI). Briefly, DPPH solution was prepared by dissolving 10 mg of DPPH powder in 200 mL methanol ([DPPH] = 0.05 mg/mL). 400 µL of each of the eight BCE (at 1mg/mL concentration) were added to 1.6 mL of methanol to obtain a stock solution. Cascade dilutions to 1/2 were made from the stock solution into test tubes containing 1 mL of methanol beforehand. Then, 1 mL of DPPH was added to each of the tubes. Absorbencies were measured at 517 nm after 30 min incubation at room temperature in the dark against a blank. Ascorbic acid (vitamin C) was

used as reference. The ability to scavenge DPPH radical (RSA) was calculated by the following equation:

% RSA (Relative Scavenging Activity) = $[(A_{control} - A_{sample})/A_{control}] \times 100$.

A = Absorbance at 517 nm. The IC_{50} (concentration providing 50% inhibition) of BCE and standards was determinate using regression curves in the linear range of concentrations. The AAI was then calculated as follows:

AAI (Antioxidant Activity Index) = [DPPH] f (μg. mL^{-1})/IC50 (μg.mL^{-1}).

[DPPH] f is the final concentration of DPPH. We considered criteria of Scherer and Godoy [52] according to which plant bark crude extracts show poor antioxidant activity when AAI < 0.5, moderate antioxidant activity when AAI between 0.5 and 1.0, strong antioxidant activity when AAI between 1.0 and 2.0, and very strong when AAI > 2.0.

2.6.1.2. Phosphomolybdenum complex method for total antioxidant capacity

Spectrophotometric evaluation of total antioxidant activity was carried out through the formation of a phosphomolybdenum complex. The assay was based on the reduction of Mo (VI) to Mo (V) and subsequent formation of a green phosphate/Mo (V) complex in acid pH [53, 54]. A total volume of 0.3 mL of each bark crude extract (at 5mg/mL concentration) dissolved in methanol was added to 3 mL of reagent solution (0.6 mol/L H_2SO_4, 28 mmol/L Na_3PO_4 and 4 mmol/L ammonium molybdate). The mixtures were incubated at 95 °C for 90 min the cooled to room temperature. The absorbance was measured at 695 nm. The total antioxidant activity of each plant BCE was expressed as the number of equivalence of ascorbic acid (mg AAE/g).

2.6.1.3. β-Carotene bleaching assay

The β-carotene–linoleic acid model system is prepared by dissolving 0.5 mg β-carotene in 1 mL chloroform followed by the addition of 40 µL linoleic acid and 500 µL Tween-20. A rotary evaporator was used to completely evaporate the chloroform added into the system. To the resulting solution, 100 mL of oxygenated distilled water is added followed by vigorous shaking. 500 µL of BCE (at 1mg/mL concentration) or antioxidant solution of the reference (Ascorbic acid; 1mg/mL concentration) was added to 2.5 mL of the previous emulsion. The absorbance was measured at 490 nm before and after heat treatment with regular time intervals for 48 h. The measurement of the absorbance continued until the color of β-carotene disappears [55].

The bleaching analysis of β-carotene was calculated as follows:

RAA (Relative Antioxidant Activity) = [Abst: $_{48h}$ (sample)/Abst: $_{48h}$ (Vit C)] × 100%.

2.6.2. Antimicrobial Activity of Bark Crude Extracts of Selected Species Consumed by Western Lowland Gorilla

2.6.2.1. Bacterial Strains Tested Growth Conditions and Inoculums Standardization

Ten MDR *E. coli* (DECs) isolates obtained in a previous study [56] were used to assess the plant studied BCE antimicrobial activity. The cultures were held at 4 °C on Muller-Hinton agar (bioMérieux, France). Colonies from 24-h cultures were used to make the inoculum suspension. The colonies were vortexed for 15 s after being suspended in sterile saline (0.9% NaCl). The turbidity of a 0.5 McFarland Norm (equivalent to 1–5 10^8 CFU/mL) was used as density setting [57].

2.6.2.2. Antimicrobial Activities

To test the antimicrobial activity of BCEs and the antimicrobial susceptibility, antibiograms were performed using the Kirby–Bauer disk diffusion method, according to the CLSI (Clinical and Laboratory Standards Institute) protocols [58]. The agar was suspended in distilled water, heated to complete dissolution autoclaved at 121 °C, and poured into Petri dishes. Whatman paper discs (6 mm) were inoculated with suspensions (10^8 CFU/mL) and dispersed on the surface of Mueller-Hinton agar plates (bioMérieux, France) [59]. Then, the discs were impregnated with 20 µL of each bark crude plant extracts. All plates were incubated for 24 h at 37 °C. The diameters of the inhibition zones were determined after incubation. Antimicrobial activity was estimated after incubation at 37 °C for 24 h by measuring the zone of inhibition against the tested microorganisms. Antimicrobials tested were amoxicillin/clavulanic acid (AMC, 30 µg) and gentamycin (GEN, 10 µg). Breakpoints provided by the CLSI were used for the designation of isolates as resistant (R), intermediately susceptible (IS) or susceptible (S). For subsequent data analysis, the isolates with an I result were grouped with the isolates that gave an R result and defined as resistant. Multidrug-resistant isolates were identified based on the definition of MDR as bacteria that are resistant to three or more classes of antimicrobial agents [60, 61].

2.6.2.3. Minimum Inhibitory Concentration (MIC) and Minimal Bactericidal Concentration (MBC) assays

In microplate wells (96 wells), 10 µL of each dilution of plant bark crude extracts varying from 30 mg/mL to 0.014 mg/mL were combined with Mueller Hinton broth (bioMérieux, France)

(170 µL) and bacterial inoculums (20 µL) and set to a final microbial concentration of 5×10^5 CFU/mL according to NCCLS standards methods, with some modifications [62]. The ethanol content in each well was less than 3.5% in an overall amount of 200 µL. For the negative control, the same percentage of ethanol was used. The MIC is the lowest concentration that does not emit a red color after 2 h of incubation. To assess MBC, a portion of each well where the concentrations are > or = (MIC) was sub-cultured on Muller-Hinton agar (MHA) (bioMérieux, France) and incubated for 24 h at 37 °C. The MBC is described as the extract concentration at which 99.9% of the inoculated bacteria were destroyed [63].

2.7. Data Analysis

All tests were performed as triplicate and the results are showed present as the mean. Excel software for Microsoft was used to analyze data. Analysis of variance (ANOVA) followed by Student's tests were used to test for significant differences between means. Differences were considered statistically significant at $p < 0.05$. Adobe Illustrator software was used to plot the histograms. The Pearson correlation was determined between the antimicrobial and antioxidant activity of plant BCE and total phenolic, flavonoid, proanthocyanidin and tannin content. To visualize the correlation data, a heatmap of three colors: red ($r = -1$), white ($r = 0$) and blue ($r = -1$) analysis was plotted with the packages Gplot using R 4.0.2.

3. Results

3.1. Ethnobotanical and ethnopharmacological survey

Western lowland gorilla living in MDNP consumed 27 plants [30]. Local people use different parts of these plants (bark, root, fruit and leaves) in medicinal preparation (maceration, decoction, lotion, pomade and infusion) (Table 1). The cross-referenced results of ethnobotanical and ethnopharmacological surveys on the traditional use of plants by traditional healers in their pharmacopoeia to treat various human illnesses enabled us to select the four plants consumed by western lowland gorillas living in MDNP for this study. This information was recovered from autochthone Vungu people, living in Doussala village in MDNP.

Table 1. Plant species consumed by gorillas and used by humans to treat various diseases including diarrhea in southeast Gabon.

Species	Family	Local name in Vungu	Part used	Preparations	Route of administration	Indication	UV	RFC	Nature
Cissus dinklagei	Verbenaceae	eéko / léko	Fruit, liana, leave	Raw, dried, cooked	Oral administration, ocular route	myopia, yellow fever, stomach pain	0,12	0,02	Tree
Klainedoxa gabonensis	Irvingiaceae	mougoma	Fruit, leave, seed, bark	Raw, dried, decoction, eaten, fresh	Oral administration, cutaneous application, lotions	analgesic, venereal disease, sterility, impotence	0,12	0,026	Tree
Cola sp.	Sterculiaceae	fudi	Seed, fruit	Mastication	Oral administration	stimulant, appetite suppressant	0,07	0,0315	Tree
Staudtia gabonensis	Myristicaceae	mobé / mibé	Fruit, bark	Decoction, macération	Oral administration	haemostatic, intestinal worms, anemia, menstruation	0,15	0,0178	Tree
Diospyros sp.	Ebenaceae	mufi nzi	Fruit, leave, seed	Decoction, macération, drink	Oral administration	albumin regulation, cysticercosis, intestinal parasites, toxoplasmosis, allergies, emphysema	0,22	0,02	Tree
Pycnanthus angolensis	Myristicaceae	mulomba	Fruit, leave, liana, seed, bark	Decoction, eaten, macération, drink	Oral administration, cutaneous application, lotions	stomach pain, enema, madness, epilepsy, gastritis	0,19	0,0315	Tree
Xylopia guintasii	Annonaceae	M'voma	Bark, root	Decoction, eaten, macération, drink, powdered	Oral administration, cutaneous application, lotions	broncho-pneumonic affections, febrile pains, knot-like swellings, treatment of pyorrhoea, ulcers	0,19	0,013	Tree
Diospyros spp.	Ebenaceae	nemba / ilalaba	Bark, root, stem, fruit	Decoction, macération	Oral administration	antibacterial, antifungal	0,07	0,02	Tree
Meiocarpidium lepidotum	Annonaceae	depeyrie	Bark, root, fruit	Decoction, macération	Oral administration	fever, purgative, abdominal pain, worm infections in babies	0,15	0,016	Tree
Duboscia macrocarpa	Tiliaceae	Moupighi	Fruit, leave, seed, bark	Decoction, macération, infusion	Oral administration	toothache, coughing, tuberculose, vermifuge, stomach pain	0,19	0,026	Tree
Ficus ssp	Moraceae	divevenguengui	Fruit, leave, seed, bark	Decoction, macération, infusion, latex	Oral administration, cutaneous application, lotions	painful periods, uterine care, local application	0,12	0,0352	Tree
Trichilia prieureana	Meliaceae		Fruit, leave, seed, bark	Decoction, macération, infusion	Oral administration, cutaneous application, lotions	venereal diseases, fever, coughs, constipation, poisoning, ascites, aphrodisiac, lumbago, rheumatism	0,34	0,013	Tree

Lannea welwitschii	Anacardiaceae	gongo	Fruit, leave, seed	Decoction, macération	Oral administration	AIDS, opportunistic diseases, respiratory tract infections, toothache, hypertension	0,19	0,02	Tree
Ricinodendron heudelotii	Euphorbiaceae	ndjoé	Fruit, leave, seed, bark	Decoction, macération	Oral administration	AIDS, opportunistic diseases, respiratory tract infections, toothache, hypertension, stomach pain	0,22	0,0178	Tree
Milicia excelsa	Moraceae	Kambal / Iroko	Leave, root, bark, fruit	Decoction, macération, infusion, latex	Oral administration, cutaneous application, lotions, ocular route	filariose, schizophrenia, diarrhea, infertility, fortifiant	0,19	0,0352	Tree
Panda oleosa	Pandaceae	ovaga	Leave, root, bark, fruit, seed	Decoction, macération, infusion	Oral administration	burns, diarrhea, dysmenorrhea, milk purification for nursing mothers, analgésique	0,19	0,016	Tree
Caloncoba welwitschii	Flacourtiaceae	myanmongom	Leave, root, bark, fruit, seed	Decoction, macération, infusion	Oral administration	asthma, heavy, painful periods, harmless madness, biliary disorders	0,19	0,02	Tree
Irvingia gabonensis	Irvingiaceae	andok / Mwiba	Fruit, leave, seed, bark	Raw, dried, decoction, eaten, fresh, macération	Oral administration, cutaneous application, lotions	deconstipant, diarrhea or dysentery, toothache, astringent	0,15	0,013	Tree
Myrianthus arboreus	Moraceae	dibimbi / mububa	Fruit, leave, seed, bark	Raw, dried, decoction, macération	Oral administration	infertility, stomach ulcer, sore, gonorrhea, hypertension, cough, anemia	0,26	0,0352	Tree
Hexalobus crispifl orus	Annonaceae	tsago	Leave, root, bark, fruit	Decoction, macération	Oral administration	venereal diseases, paludisme, wounds, boils, fever, muscle pain, arthritis, convulsion	0,3	0,0315	Tree
Sacoglottis gabonensis	Humiriaceae	Ozouga	Fruit, bark, fruit	Decoction, macération	Oral administration	AIDS, opportunistic diseases, venereal diseases	0,12	0,0123	Tree
Uapaca guineensis	Euphorbiaceae	Assam	Fruit, bark, fruit, root	Decoction, macération	Oral administration	intestinal parasitism, anti-abortive, aphrodisiac, restorative for young mothers, leprosy, gastrointestinal disorders	0,22	0,02	Tree
Ceiba pentandra.	Malvaceae	Mufuma	Fruit, bark, fruit, root, leave	Decoction, macération, infusion	Oral administration, baths	colic, inflammation, poisoning, fatigue, diurétique, hydropisia	0,22	0,0352	Tree
Diospyros mannii	Ebenaceae	Emba	Fruit, leave, seed, bark	bark shavings,	Cutaneous application, lotions	chest pain	0,04	0,026	Tree
Dichostema glaucescens	Euphorbiaceae	Dibule / Mabule	Bark	maceration, powdered	Oral administration	bad luck, purification bath, nurturing galactogen, emetic	0,15	0,016	Tree

Eriocoelum macrocarpum	Sapindaceae	Dibotsa	Bark, root	Decoction	Oral administration	female infertility, food cooking liquid	0,08	0,0178	Tree
Synsepalum dulcifi cum	Sapotaceae		Fruit	Raw, dried, cooked	Oral administration	suppress sensations of acidity and bitterness	0,04	0,026	Tree

Legend: UV: Use Value indicates the relative importance of uses of plant species. RFC: Relative Frequency of Citation indicates the local importance of each species.

~~**Table 2:** Ethnobotanical and ethnopharmacological informations about selected plants, consumed by western lowland gorilla living MDNP and used as medicinal plants (MPs) in traditional medicine by healers in Gabon.~~

Local name in Vungu	Botanical name	Family	Parts used	Mode/form of consumption	Medicinal Applications	References
Kambala	***Milicia excelsa***	Moraceae	Fruits, seeds, leaves, and bark	Raw, dried, semicooked, decoction	Antioxidant, antimicrobial, anti-inflammatory, gastrointestinal, calmative, analgesic	[42, 64]
Divevengui blanc	***Ficus* ssp**	Moraceae	Milky latex, fruits and bark	Raw, dried, decoction, eaten, fresh	Antioxidant, antimicrobial, anti-inflammatory, antiviral, antidiarrhoeal, antifungal, anticancer, antiplasmodial, antiulcer, lannoite	[41, 65]
Mububa	***Myrianthus arboreus***	Moraceae (Cecropiaceae)	Fruits, seeds, leaves, and bark	Raw, decoction, and dried	Antioxidant, antimicrobial, anti-inflammatory, antifungal, antidiabetic, antinociceptive	[40, 66]
Mufuma	***Ceiba pentandra.***	Malvaceae (Bombacaceae)	Fruits, seeds, leaves, and bark	Raw, decoction, and dried	Antioxidant, antimicrobial, anti-inflammatory, anticancer, analgesic, anti-hyperglycemic	[39, 67]

Legend: Vungu, also called ghivungu, is a Bantu language spoken by the Vungus in Gabon, including in MDNP

~~*3.2. Extraction Yields*~~

Extraction yields varied according to the solvents used (Table 3). Water proved more efficient with higher yields than ethanol.

Table 3. Percentage yield of 100 g of bark in 2000 L solvent from *Ceiba pentandra*, *Myrianthus arboreus*, *Ficus ssp* and *Milicia excelsa* BCE.

Botanical name	Extract mass (g)	% Yield (g)
Ceiba Pentandra		
AE	1.93	1.93 ± 0.11^{a}
EE	1.58	1.58 ± 0.12^{b}
Myrianthus arboreus		
AE	2.76	2.76 ± 0.23^{c}
EE	1.15	1.15 ± 0.14^{d}
***Ficus* ssp**		
AE	2.26	2.26 ± 0.27^{e}
EE	1.31	1.31 ± 0.21^{f}
Milicia excelsa		
AE	1.52	1.52 ± 0.33^{g}
EE	1.51	1.51 ± 0.22^{h}

Legend: EE: Ethanol extract; AE: Aqueous extract. Values are means ± standard deviation of triplicate analysis. In all cases, the difference between extraction methods is statistically significant ($P < 0.05$).

3.3. Preliminary Phytochemical Screening

Phenolic compounds, alkaloids, flavonoids, tannin gallic, anthracenosides, reducing sugar, coumarins, sterols and triterpenes, oses and holosides, cyanidines, cardiac glycosides and saponins were found in almost all plant BCEs tested (Table 4).

Table 4. Phytochemical screening of *Ceiba pentandra*, *Myrianthus arboreus*, *Ficus* ssp and *Milicia excelsa*.

**Secondary metabolites**		**Biological activity**	***Ceiba Pentandra***		***Myrianthus arboreus***		***Ficus* ssp**		***Milicia excelsa***	
			AE	EE	AE	EE	AE	EE	AE	EE
Saponins		Antidiarrhoeal	++	+	++	+	++	+	-	-
Tannin gallic		Antioxidant	+++	++	+++	++	++	++	++	++
Tannin catechin		Antidiarrhoeal	++	++	++	++	++	++	++	++
		Antimicrobial								
Total phenolics		Antioxidant	+++	++	+++	++	+++	++	+++	++
		Antimicrobial								
Total flavonoids		Antimicrobial	++	++	++	+	-	-	++	++
		Anti-inflammatory								
		Antioxidant								
Reducing sugars		Provide and store energy	+++	++	+++	++	+	++	++	++
Alkaloids		Antidiarrhoeal	+++	++	+++	++	+++	++	+++	++
		Antihelmintic								
		Antimicrobial								
Anthracenosides		Antimicrobial	+++	++	++	++	++	++	-	-
		Anti-inflammatory								
Coumarins		Antimicrobial	+++	++	+++	++	+++	++	+++	++
		Antioxidant								
Sterols and Triterpenoids		Cholesterol reduction	+	+	+	-	+	-	+	-
Oses and holosides		Anti-inflammatory	+++	++	-	+	+	++	++	++
		Antitumor/Anticancer								
Cardiac glycosides		Cardiotonic/ diuretic	+++	++	+++	++	++	++	+++	++
	Digitoxins	Cardiotonic	+++	++	+++	+	+++	++	+++	++
	Gitoxins	Cardenolide steroid	-	-	-	-	++	++	++	++
	Gitoxins genins	Cardenolide steroid	-	-	+++	++				
Cyanidins		Anti-inflammatory	+++	++	+++	++	-	-	+++	++

Flavonols	Antioxidant	-	-	-	-	-	-	-	-
Flavones	Antioxidant	+++	+	++	+	-	-	-	-
Flavonones	Antioxidant	-	-	-	-	-	-	++	++

Legend: +++ = very abundant; ++= abundant; + = not abundant, - = not detected. EE: Ethanol extract; AE: Aqueous extract.

3.4. Antioxidant Potential of Bark Crude Extracts of Selected Species Consumed by Western Lowland Gorilla

The total phenolic contents (standard curve equation: Y = 0.001 2X + 0.000 4, R^2 = 0.998 2) from the ethanolic and aqueous BCE of the four plant species varied from 110.55 ± 26.05 to 386.38 ± 4.11 mg GAE/100 g of extract. *Myrianthus arboreus* ethanolic BCE had higher phenolic content than other extracts. *Ficus ssp* aqueous BCE had the lowest phenolic content (Figure 2a).

Total flavonoid contents (standard curve equation: Y = 0.003 2X + 0.007 7, R^2 =1) were greater in *Milicia excelsa* ethanolic BCE [(368.11 ± 2.54) mg QE/100 g of extract] than in other plant extracts (Figure 2a).

Proanthocyanidins (calibration curve Y = 0.000 6X + 0.002 4 with R^2= 0.986) were higher in *Ceiba pentandra* ethanolic BCE [(208.05 ± 2.09) mg APE/100 g of extract] than in other plant extracts (Figure 2b).

Tannins contents (standard curve equation: Y = 0.000 9X + 0.208 8, R^2 = 1) were highest in *Myrianthus* ethanolic extract, (381.66 ± 14.45 mg TAE/100 g of extract] (Figure 2b).

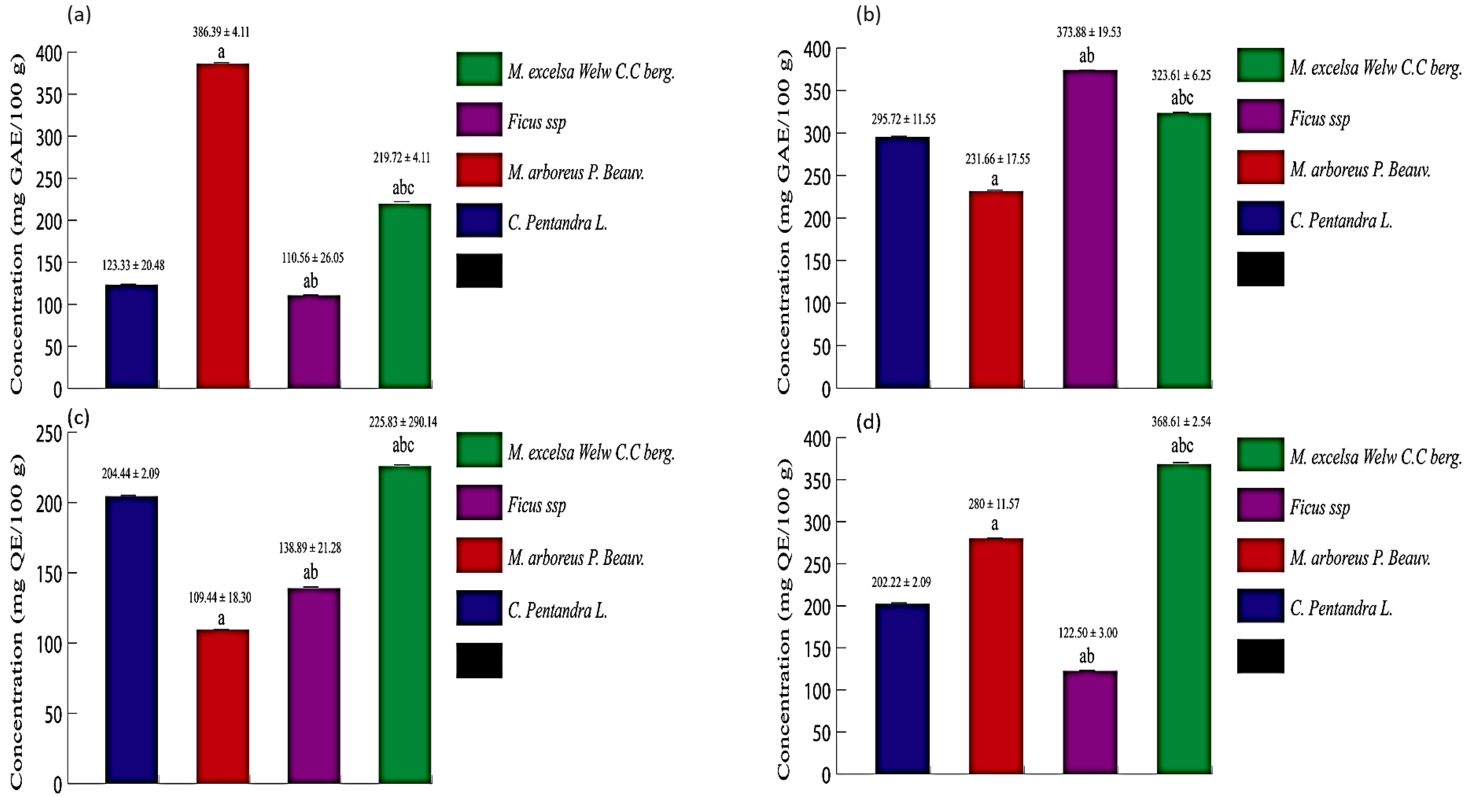

Figure 3a: Total phenolic and flavonoid content of *Ceiba pentandra*, *Myrianthus arboreus*, *Ficus* ssp and *Milicia excelsa* BCEs. (a): Total phenolic content of plant bark crude aqueous extracts. (b): Total phenolic content of plant bark crude ethanolic extracts. (c): Total flavonoid content of plant bark crude aqueous extracts. (d): Total flavonoid content of plant bark crude ethanolic extracts. [a] $p < 0.05$ compared to *Ceiba pentandra* BCE in the same column; [b] $p < 0.05$ compared to *Myrianthus arboreus* BCE in the same column; [c] $p < 0.05$ compared to *Ficus* ssp BCE in the same column; GAE=gallic acid equivalent; QE= quercetin equivalent; TAE= tannic acid equivalent; APE =apple procyanidins equivalent.

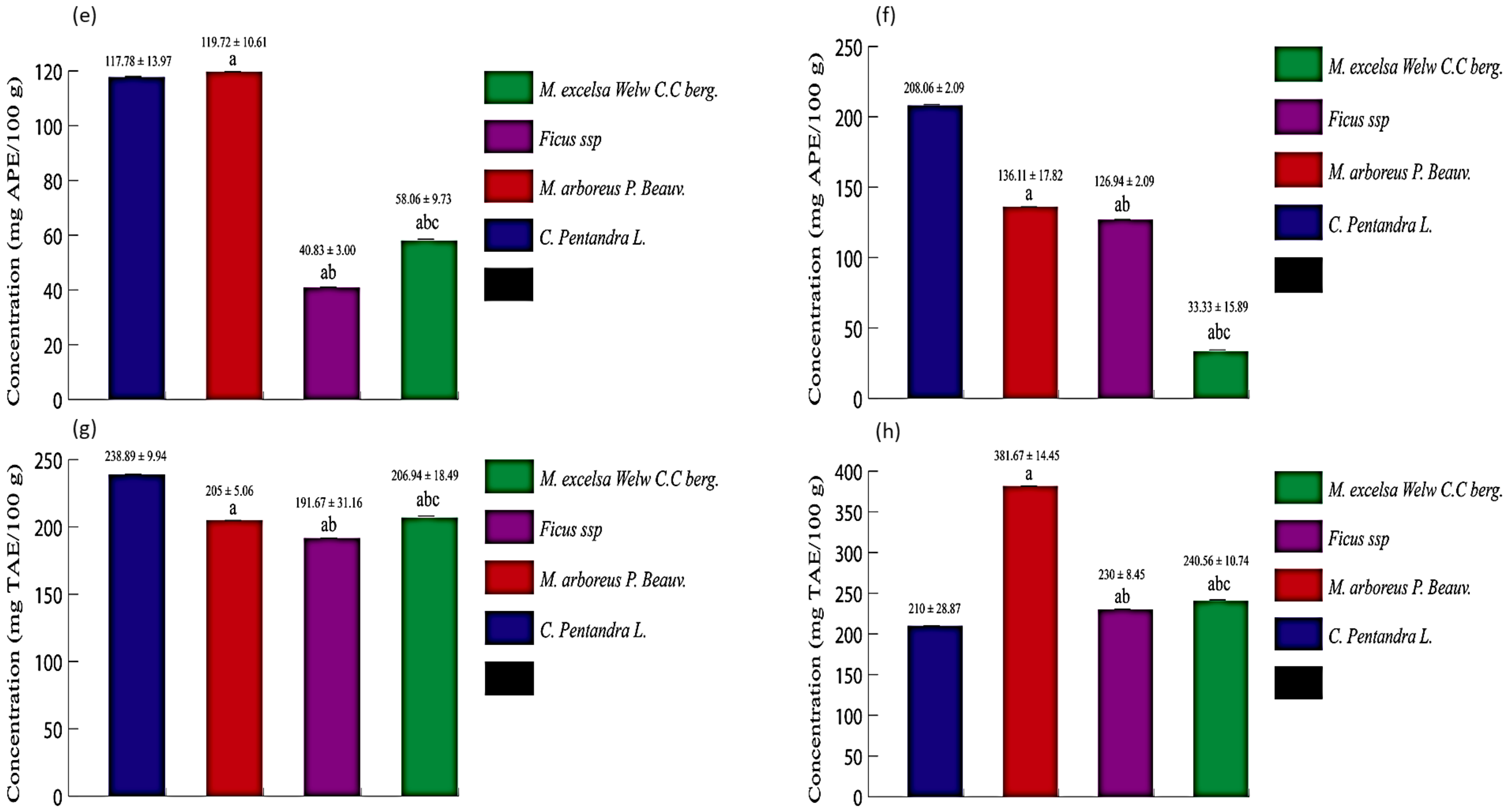

Figure 3b: Total proanthocyanidin and tannin content of *Ceiba pentandra*, *Myrianthus arboreus*, *Ficus* ssp and *Milicia excelsa* BCE. (e): Total proanthocyanidin content of plant bark crude aqueous extracts. (f): Total proanthocyanidin content of plant bark crude ethanolic extracts. (g): Total tannin content of plant bark crude aqueous extracts. (h): Total tannin content of plant bark crude ethanolic extracts. . [a] $p < 0.05$ compared to *Ceiba pentandra* BCE in the same column; [b] $p < 0.05$ compared to *Myrianthus arboreus* BCE in the same column; [c] $p < 0.05$ compared to *Ficus* ssp BCE in the same column; GAE=gallic acid equivalent; QE= quercetin equivalent; TAE= tannic acid equivalent; APE =apple procyanidins equivalent.

3.5. Antioxidant Activity of Bark Crude Extracts of Selected Species Consumed by Western Lowland Gorilla

3.5.1. DPPH radical scavenging activity

The AAI of the bark crude extracts from *Ceiba pentandra* ranged from 9.3 ± 1.28 to 10.17 ± 3.45. For *Myrianthus arboreus* it ranged 11.81 ± 0.30 to 13.72 ± 6.88. From *Ficus* ssp it ranged 7.11 ± 1.16 to 19.45 ± 2.98, and from *Milicia excelsa* it ranged 7.11 ± 4.64 to 11.98 ± 3.56 (Table 5). These values are lower than the AAI of Ascorbic acid (23.16 ± 20.26).

3.5.2. Phosphomolybdenum complex assay

Myrianthus arboreus aqueous extract showed the highest total antioxidant activity with 152.06 ± 19.11 mg AAE/g and *Ceiba pentandra* aqueous extract showed the lowest value with 54.73 ± 12.48 mg AAE/g (Table 5).

3.5.3. β-Carotene bleaching assay

The β-carotene bleaching assay results (Table 5) and β-carotene bleaching kinetics (Figure 3) showed that the highest relative antioxidant activity (RAA) was shown by *Ceiba pentandra* ethanolic extract (RAA = 73.25 ± 5.29) and *Ficus* ssp aqueous extract (RAA = 38.67 ± 0.27). *Ficus* ssp and *Myrianthus arboreus* ethanolic extract showed lower activities (RAA = 31.79 ± 0.57).

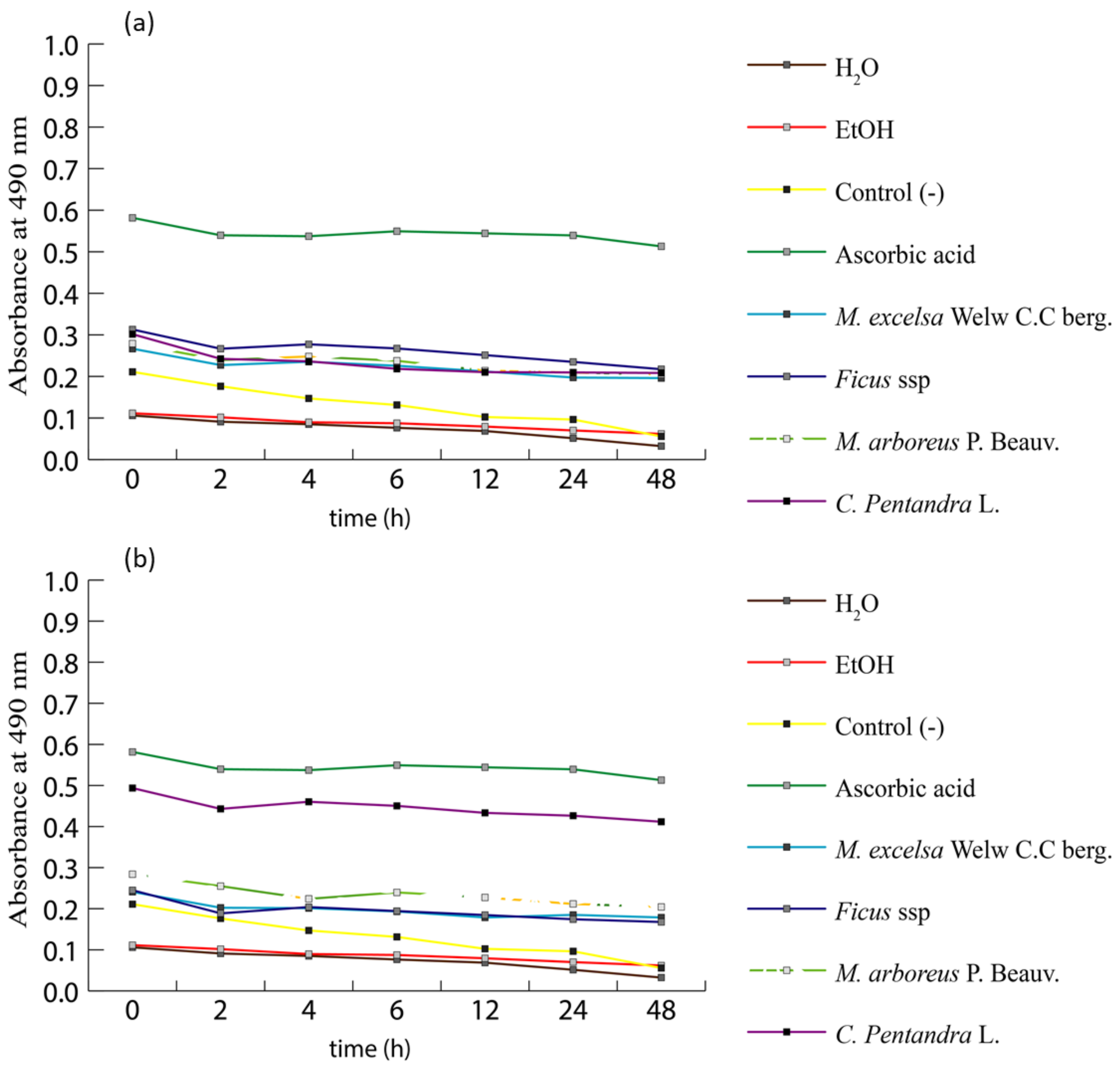

Figure 4: β-carotene bleaching kinetics of (0.5 mg) at 490 nm from *Ceiba pentandra*, *Myrianthus arboreus*, *Ficus* ssp and *Milicia excelsa* BCE (500 µL) and Ascorbic acid. (a): Plant aqueous BCE. (b): Plant ethanolic BCE. Each value is the mean of three analyses.

Table 5. Antioxidant activities of *Ceiba pentandra*, *Myrianthus arboreus*, *Ficus* ssp and *Milicia excelsa* BCE determined by DPPH, Phosphomolybdenum complex and β-Carotene bleaching assays using L-ascorbic acid as a positive control.

Sample	DPPH IC50 (mg/mL)	DPPH AAI	PM TAC (mg AAE/g)	BCB RAA (%)
Ceiba Pentandra				
AE	2.73 ± 0.35	9.3 ± 1.28	54.73 ± 12.48	37.07 ± 0.44
EE	2.65 ± 0.87	10.17 ± 3.45	48.66 ± 12.96	73.25 ± 5.29
Myrianthus arboreus				
AE	2.12 ± 0.05^{a}	11.81 ± 0.30^{a}	152.06 ± 19.11^{a}	36.47 ± 0.64^{a}
EE	2.18 ± 1.12^{a}	13.72 ± 6.88^{a}	104.33 ± 63.25^{a}	36.35 ± 0.44^{a}
***Ficus* ssp**				
AE	$6.15 \pm 0.24^{a,b}$	$7.11 \pm 1.16^{a,b}$	$90.6 \pm 49.77^{a,b}$	$38.67 \pm 0.27^{a,b}$
EE	$1.31 \pm 0.20^{a,b}$	$19.45 \pm 2.98^{a,b}$	$92.93 \pm 20.50^{a,b}$	$29.83 \pm 0.20^{a,b}$
Milicia excelsa				
AE	$6.15 \pm 6.14^{a,b,c}$	$7.11 \pm 4.64^{a,b,c}$	$124.33 \pm 39.05^{a,b,c}$	$34.87 \pm 0.35^{a,b,c}$
EE	$2.25 \pm 0.79^{a,b,c}$	$11.98 \pm 3.56^{a,b,c}$	$104.6 \pm 36.61^{a,b,c}$	$31.79 \pm 0.57^{a,b,c}$
Ascorbic acid	$1.73 \pm 1.20^{a,b,c,d}$	$23.16 \pm 20.26^{a,b,c,d}$		$100.00 \pm 1.78^{a,b,c,d}$

Legend: DPPH: (2,2-Diphenyl-1-Picrylhydrazyl) ; IC_{50}: the concentration of extracts reducing 50% of DPPH; AAI : Antioxidant Activity Index ; PM TAC : Phosphomolybdenum Total Antioxidant Capacity ; BCB RAA : β-Carotene Bleaching Relative Antioxidant Activity. Values are expressed as means ± SD. ^{a} $p < 0.05$ compared to aqueous and ethanolic extracts of *Ceiba Pentandra*. in the same column; ^{b} $p < 0.05$ compared to aqueous and ethanolic extracts of *Myrianthus arboreus* in the same column; ^{c} $p < 0.05$ compared *Ficus* ssp in the same column; ^{d} $p < 0.05$ compared *Milicia excelsa* in the same column.

3.6. Antimicrobial Activity of Bark Crude Extracts of Selected Species Consumed by Western Lowland Gorilla

The ethanolic BCE tested showed more significant activity against MDR *E. coli* (DECs) than the aqueous BCE. Of the four plants tested, all extracts were active against at least one MDR *E. coli* (DECs) isolate (Table 6, Figure 5). *Ceiba pentandra* aqueous BCE showed remarkable activity against all the tested MDR *E. coli* (DECs) isolates with inhibition zones ranging from 8.34 ± 0.57 mm to 13.67 ± 0.57 mm (Table 6). *Ceiba pentandra* ethanolic BCE also showed reasonable activity against all the tested MDR *E. coli* (DECs) isolates with inhibition zones ranging from 8.34 ± 0.57 mm to 11.67 ± 0.57 mm. *Ficus* ssp aqueous and ethanolic BCE showed activity against MDR *E. coli* (DECs) isolates with inhibition zones ranging from 8.34 ± 0.57 mm to 10.4 ± 0.57 mm and 7.67 ± 1.54 mm to 10.67 ± 0.57 mm respectively. *Myrianthus arboreus* aqueous BCE showed activity only against two MDR *E. coli* (DECs) isolates with

inhibition zones ranging from 7.34 ± 0.57 mm to 8.34 ± 0.57 mm. *Myrianthus arboreus* ethanolic BCE showed activity against six tested MDR *E. coli* (DECs) isolates with inhibition zones ranging from 8.67 ± 0.57 mm to 10.67 ± 0.57 mm. *Milicia excelsa* Welw C.C berg. aqueous BCE were the least active. Ethanol (98%) and water were used as control and did not have any effect on all studied MDR *E. coli* (DECs) isolates.

MIC values were between 1.56 mg/mL and 50 mg/mL, and MBC values were between 3.12 mg/mL and >50 mg/mL (Table 7).

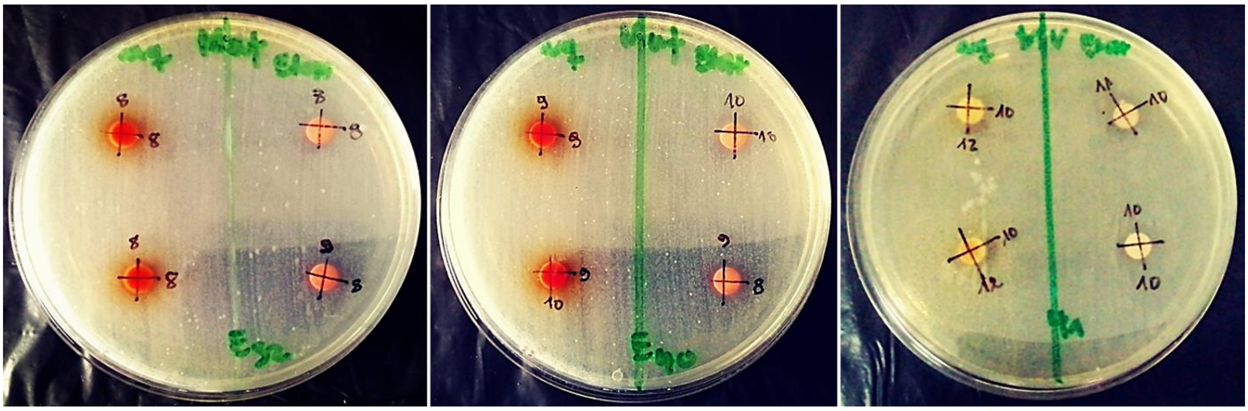

Figure 5. Diameters of the inhibition zones (mm) produced by *Ceiba pentandra*, *Myrianthus arboreus*, *Ficus* ssp and *Milicia excelsa* BCE test against MDR *E. coli* (DECs) isolates.

Table 6. Inhibition zones (mm) diameters induced by *Ceiba pentandra*, *Myrianthus arboreus*, *Ficus* ssp and *Milicia excelsa* BCE against MDR *E. coli* (DECs).

	**MDR *E. coli* (DECs)**									
**Species**	**E32**	**E40**	**E41**	**E8**	**E4**	**E7**	**E37**	**E50**	**E49**	**E10**
Ceiba Pentandra										
AE	8.67 ± 0.57	9.34 ± 0.57	8.34 ± 0.57	8.67 ± 0.57	9.34 ± 0.57	13.67 ± 0.57	10.34 ± 0.57	13.34 ± 0.57	8.34 ± 0.57	9.67 ± 0.57
EE	9 ± 0.57	9.67 ± 0.57	9.67 ± 0.57	11.67 ± 0.57	10.34 ± 0.57	11.67 ± 0.57	10.34 ± 0.57	9.34 ± 0.57	8.34 ± 0.57	10.34 ± 0.57
Myrianthus arboreus										
AE	7.34 ± 0.57	8.34 ± 0.57	ND	ND	ND	ND	ND	ND	ND	ND
EE	8.67 ± 0.57	9 ± 0.57	ND	ND	ND	10.67 ± 0.57	10.34 ± 0.57	10.34 ± 0.57	10 ± 0.57	ND
***Ficus* ssp**										
AE	ND	ND	8.67 ± 0.57	8 ± 0.57	8 ± 0.57	8 ± 0.57	9.67 ± 0.57	8.34 ± 0.57	10.4 ± 0.57	ND
EE	9 ± 0.57	10.34 ± 0.57	10.67 ± 0.57	7.34 ± 0.57	9.67 ± 0.57	7.67 ± 1.54	ND	ND	8.34 ± 0.57	9 ± 0.57
Milicia excelsa										
AE	ND	ND	ND	ND	ND	ND	ND	ND	9.34 ± 0.57	8.67 ± 1.54
EE	8.67 ± 0.57	ND	8.34 ± 0.57	8.67 ± 0.57	9.34 ± 0.57	13.67 ± 0.57	10.34 ± 0.57	13.34 ± 0.57	8.67 ± 0.57	9.67 ± 0.57
**Standards**										
AMC	9 ± 1	9.34 ± 1.15	17.67 ± 0.57	8 ± 0	14.67 ± 0.57	8 ± 0	8 ± 0	9 ± 1	9.34 ± 1.15	8 ± 0
GEN	8 ± 0	9.67 ± 1.57	18 ± 2	8 ± 0	18 ± 3.60	10 ± 1	10.34 ± 0.57	9.34 ± 1.52	9 ± 1	8 ± 0
**Control**										
Ethanol (98%)	ND	ND	ND	ND	ND	ND	ND	ND	ND	ND
Water	ND	ND	ND	ND	ND	ND	ND	ND	ND	ND

Legend: Values are expressed as means ± SD; ND: not determined, EE: Ethanol extract; AE: Aqueous extract.

Table 7. Minimal inhibitory (MIC) and minimal bactericidal (MBC) concentration (mg/mL) of *Ceiba pentandra*, *Myrianthus arboreus*, *Ficus* ssp and *Milicia excelsa* BCE.

	**E32**		**E40**		**E41**		**E8**		**E4**		**E7**		**E37**		**E50**		**E49**		**E10**	
**Sample**	**MIC**	**MBC**	**MIC**	**MBC**	**MIC**	**MBC**	**MIC**	**MBC**	**MIC**	**MBC**	**MIC**	**MBC**	**MIC**	**MBC**	**MIC**	**MBC**	**MIC**	**MBC**	**MIC**	**MBC**
Ceiba Pentandra																				
AE	12.5	25	6.25	12.5	12.5	25	12.5	25	6.25	12.5	1.56	3.12	3.12	6.25	1.56	3.12	12.5	25	6.25	12.5
EE	6.25	12.5	6.25	12.5	6.25	12.5	1.56	3.12	3.12	6.25	1.56	3.12	3.12	6.25	6.25	12.5	12.5	25	3.12	6.25
Myrianthus arboreus																				
AE	25	50	12.5	25	–	–	–	–	–	–	–	–	–	–	–	–	–	–	–	–
EE	12.5	25	6.25	12.5	–	–	–	–	–	–	3.12	6.25	3.12	6.25	3.12	6.25	3.12	6.25	–	–
***Ficus* ssp**																				
AE	–	–	–	–	12.5	25	12.5	25	12.5	25	12.5	25	6.25	12.5	12.5	25	3.12	6.25	–	–
EE	6.25	12.5	3.12	6.25	3.12	6.25	25	50	6.25	12.5	25	50	–	–	–	–	12.5	25	6.25	12.5
Milicia excelsa																				
AE	–	–	–	–	–	–	–	–	–	–	–	–	–	–	–	–	6.25	12.5	12.5	25
EE	12.5	25	–	–	12.5	25	12.5	25	6.25	12.5	1.56	3.12	3.12	6.25	1.56	3.12	12.5	25	6.25	12.5
Ethanol (98%)	–	–	–	–	–	–	–	–	–	–	–	–	–	–	–	–	–	–	–	–
Water	–	–	–	–	–	–	–	–	–	–	–	–	–	–	–	–	–	–	–	–

Legend: MIC: minimum inhibitory concentration; MBC: minimum bactericidal concentration, EE: Ethanol extract; AE: Aqueous extract; –: Not effective.

3.7. Correlations Analysis

There was a positive correlation between the overall total phenolic, flavonoid, proanthocyanidin and tannin content and antimicrobial activity and a negative correlation with antioxidant activity assessed by DPPH IC_{50}, PM TAC and BCB RAA (Figure 6).

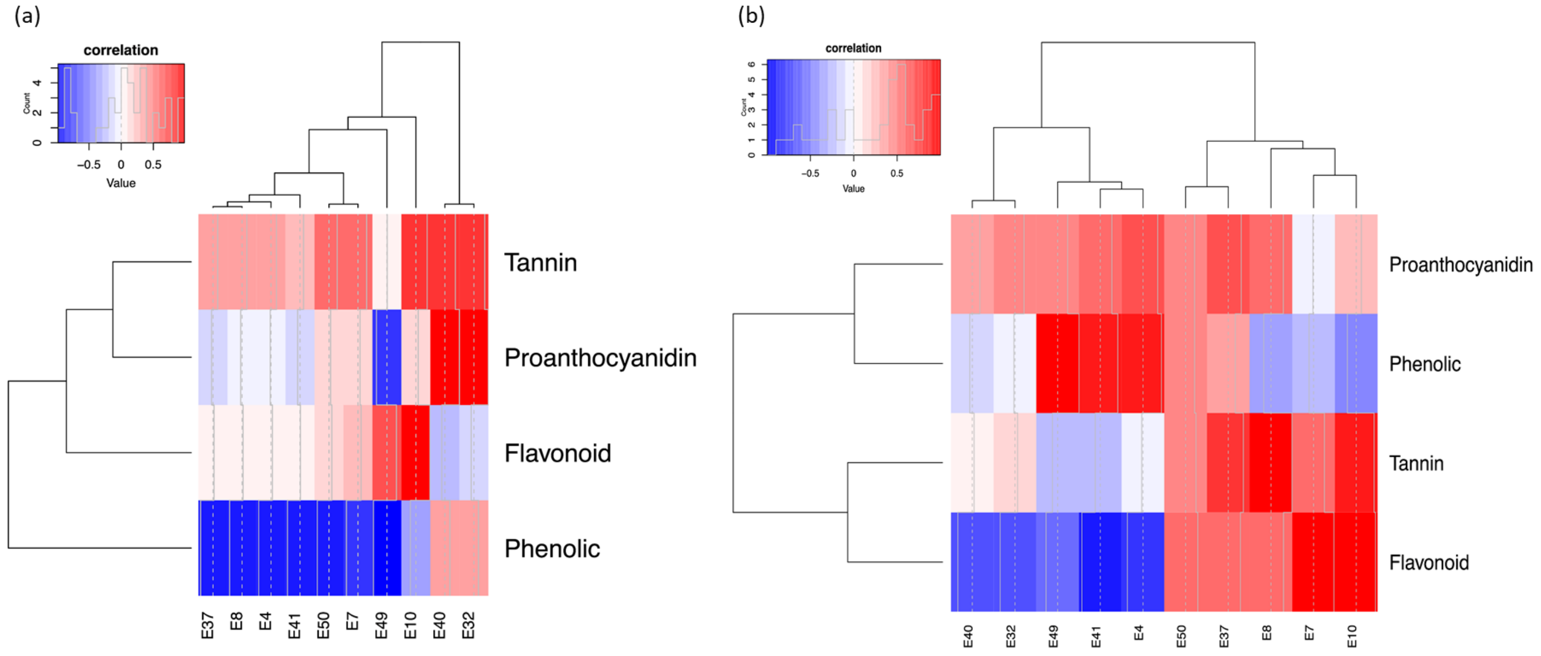

Figure 6.a. Heat map illustrating the correlation between total phenolic, flavonoid, proanthocyanidin and tannin content with the assessed antimicrobial activities. (a): Plant aqueous BCE. (b): Plant ethanolic BCE.

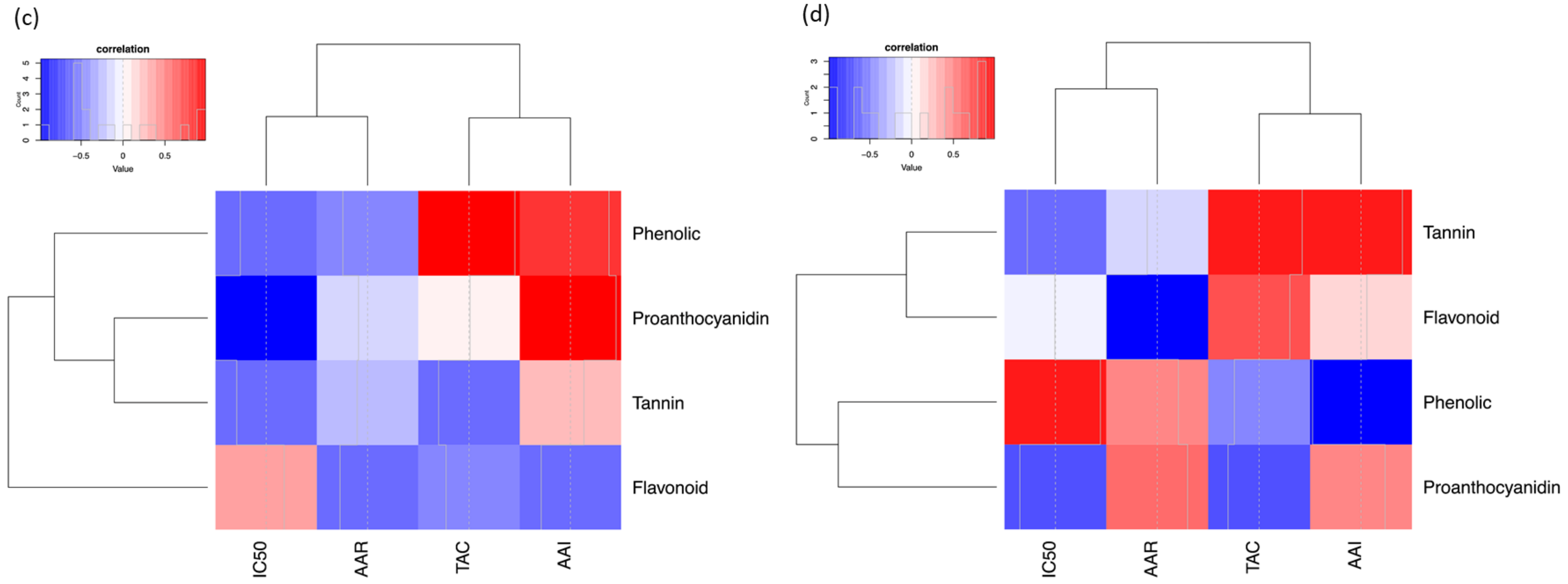

Figure 6.b. Heat map illustrating the correlation between total phenolic, flavonoid, proanthocyanidin and tannin content with the assessed antioxidant activities. (c): Plant aqueous BCE. (d): Plant ethanolic BCE.

4. Discussion

The use of MPs for their pharmacological properties and biological activities is a practice that is increasingly reported across the world [68]. The WHO estimates that more than 25% of prescription drugs derive from MPs [69]. Research on and development of evidence-based phytomedicines is a priority for Africa, including Gabon [70]. Zoopharmacognosy is an original approach that could achieve these objectives [71]. Since the introduction of zoopharmacognosy as a scientific discipline, many drugs that are now in use have been found by studying animal self-medication behaviors [2]. Great apes use plants to heal themselves and thus control their parasitemia, viremia and bacteremia [72]. Moreover, traditional healers use plants items usually consumed by great apes for their pharmacological properties and biological activities [73]. However, these traditional uses of plants are not based on Western science and analysis [74].

The results in this study showed a negative correlation between extraction yield and solvent. The percentage yield of the different solvents obtained for the four plant bark extracts studied can be compared to those reported in the literature. For *Ceiba pentandra*, studies report a yield of 0.34% of the ethanolic extract [75], and 9% for the aqueous bark extract [76]. For *Myrianthus arboreus*, the yield of the ethanolic extract was 9.87% [77], and 0.05% for leaf aqueous extract [78]. For *Milicia excelsa* the yield .65% for stem bark ethanolic extract [79]. There are no reports on the yield of *Ficus* ssp. The differences observed between the results of this study and those in the literature could be explained by the many factors taken into account during the extraction process [45].

Preliminary phytochemical screening revealed the presence of several classes of secondary metabolites in the bark extracts of the four plants consumed by gorillas living in MDNP. Similar results have been obtained in other studies of the phytochemical content and antimicrobial activities of *C. pentandra* extracts [67, 80]. For *Myrianthus arboreus*, studies in Cameroon [81] and Ghana [82] showed comparable results to those obtained here. One study showed only the presence of phenolic compounds and reducing sugars [83]. Another detected phenolic compounds and flavonoids [84]. *Ficus* spp are used as medicine to reduce the risk of cancer and heart disease [85]. Comparable results to those obtained here have been reported for *Ficus* species in Tunisia [86], Turkey [87] and Italy [88]. For *Milicia excelsa* BCE, a study in Nigeria, showed the presence of tannins, alkaloids, flavonoids and saponins in Nigeria [89]. Flavonoids and phenolic compounds were also found in Nigeria [90]. The secondary metabolites found in the BCE of the four plants studied possess pharmacological properties and are endowed with

biological activities, suggesting that they can be used in traditional medicine as MP by healers and in pharmaceutical products [91, 92].

Traditional African healers make use of MPs to treat microbial diseases without western scientific basis [93, 94]. The results for total phenolic, flavonoid and tannin content obtained here in *Ceiba pentandra* are consistent with those reported in recent studies [39, 76]. For *Myrianthus arboreus*, total phenolic and flavonoid content [95], proanthocyanidins content [96] and tannins content [97] have been found as in this study. Recent work on the genus Ficus has also revealed the presence of total phenolic compounds and total flavonoids [98], and total tannins [99]. Similar results to those obtained here have been reported for *Milicia excelsa* extracts [100]. Therefore, the use of these four plant species in traditional medicine could be attributed to the high content of phenolic [101], flavonoids (flavones and flavonones) [102], tannins [103] and proanthocyanidins [104] compounds which are known for their antimicrobial, antioxidant, anticancer, antidiarrheal, anti-inflammatory, antimalarial, anti-cytotoxic and antispasmodic activities. The phenolic, flavonoid tannin and proanthocyanidin compounds found in this study may justify their bioactive effects for diarrheal diseases, those related to oxidative stress caused by ROS and diseases related to MDR *E. coli* (DECs).

In this study, DPPH assay showed that the radical scavenging activity of the BCEs is dose-dependent. All plant BCE studied here have DPPH radical scavenging activities. *Ficus ssp* ethanolic BCE showed a DPPH scavenging activity (IC_{50}) (1.31 ± 0.20 mg/mL) higher than *Ficus brevibracteata* leaves with a IC_{50} value of 1.30 µg/ml (85.18 ± 0.22 % of DPPH scavenging) [105], and lower than *Ficus variegata* stem bark ethanolic extract with 91% of DPPH scavenging [106]. Studies show a high relative scavenging activity (RSA) (about 50 % of DPPH scavenging and 73.7 ± 8.4% of DPPH scavenging) [81, 107, 108] and *Myrianthus arboreus* aqueous extract has antioxidant activities [78]. In addition, antioxidant activities results obtained in the present study for *Myrianthus arboreus* are close to those observed in a previous study, which obtained a IC_{50} value of 13.3 µg/mL for the ethanolic extract [96]. For *Ceiba pentandra* ethanolic BCE, we observed similar antioxidant activities to those in a previous study, where the DPPH radical scavenging activity of ethanolic *Ceiba pentandra* leaves extract increased in dose dependent manner ranging from 10-50 µg/ml [109]. For *Ceiba pentandra* aqueous BCE, studies have obtained comparable results from aqueous extracts of stem bark [110] which induced also a concentration-dependent radical scavenging activity on DPPH, trunk [111] and leaf bark extracts [112]. *Milicia excelsa* showed radical scavenging activity on DPPH [113, 114]. Previous studies highlighted total antioxidant activities using

phosphomolybdenum total antioxidant activity assay of *Ficus nota* [115], *Ficus carica* which possessed lower reducing ability with EC_{50} value of 39 µg/ml [116], *Ficus sycomorus* with EC_{50} value of 25 µg/ml [116] and *Ficus Benghalensis* methanolic extract with a IC_{50} value for phosphor-molybdenum of 31.84 ± 0.12 µg/ml [117]. A recent study showed *Myrianthus arboreus* aqueous root bark extract (40.3 ± 3.9 mg TE/g) and ethanolic extract (161.1 ± 11.9 mg TE/g) total antioxidant activities using phosphomolybdenum total antioxidant activity assay [96]. Sinha et al. reported *Bombax ceiba* (*Ceiba pentandra*) flowers phenolic extract total antioxidant activities using phosphomolybdenum complex assay [118]. There have been no reports on the effects of *Milicia excelsa* BCE using this assay. The β-carotene bleaching activities of all plant BCE depended on concentration. The antioxidant activity was observed in order of *Ceiba pentandra.*> *Myrianthus arboreus*> *Ficus ssp*> *Milicia excelsa*. There are no reports in the literature on the antioxidant activities of the BCEs of the four plants we studied using β-carotene bleaching assay. Plant antioxidants are a natural reservoir of bioactive compounds and play important roles in plant acclimation and adaptation to environmental challenges, and are also beneficial to human health [8]. Recent work on pollen extracts of three plants from the Palestinian pharmacopoeia reported antioxidant activities of flavonoids compounds such as flavones and flavonols in these extracts [47]. The antioxidant capacities of these all secondary compounds support the human body's battle against diseases by absorbing free radicals and chelating metal ions that could catalyze the production of ROS, which facilitates lipid peroxidation [119]. Antioxidants break radical chain reactions, preventing oxidative stress-related damage [120]. In brief, antioxidants can act according to two major mechanisms, either by transfer of hydrogen atom or by electron transfer [121].

The inhibitory activity of MPs extracts against Gram-positive and Gram-negative bacteria has been widely highlighted in the literature [122], and MPs extracts with MIC values less than 100 mg/mL can be considered to have very good antimicrobial activity [123]. Results of the antimicrobial activities from the four plant BCEs investigated against MDR *E coli* (DECs), found in this study are comparable to those reported in the literature. *Ceiba pentandra* ethanolic extract showed antimicrobial activities against *E. coli* (an organism frequently implicated in gastroenteritis and pelvic inflammation) with a zone of inhibition value of 10.55 ± 1.45 mm and showed a MIC value of 12.5 mg/ml against *E. coli* [75]. A previous study also showed that *Ceiba pentandra* ethanolic extract was highly active against *E. coli* and that the antimicrobial activity of leaf extract increased as the concentration of ethanolic extract of *Ceiba pentandra* leaves increased [109]. Parulekar et al. [80] reported moderate antimicrobial activities from

aqueous extract and strong antimicrobial activities ethanolic extract against *E. coli*, which increased as the concentration of the extracts increased; and Njokuocha et al. [67] also reported antimicrobial activities of *Ceiba pentandra* extracts. *Ceiba pentandra* aqueous extract has shown antimicrobial activities against Gram-negative bacteria, including *E. coli* [67, 80]. A recent study showed that the mean zone of inhibition of *Myrianthus arboreus* was zero for *E. coli* ATCC 25922 with aqueous and ethanolic extracts. However, for clinical MDR isolates, the *Myrianthus arboreus* inhibition zone diameter value was 0.4-2.2 mm for ethanolic extracts, showing low antimicrobial activities [83]. Ethanolic extracts of taxa belonging to the genus Ficus have been studied for their antimicrobial potential [124]. Biologically, *Milicia excelsa* Welw C.C berg. extract antimicrobial activities against enterobacteria have been demonstrated [125]. The flavonoid neocyclomorusin isolated from *Milicia excelsa* extract exhibited antimicrobial activity against *K. pneumoniae* ATCC11296 and *E. cloacae* BM47, with MIC values of 4 µg/mL each [42]. Padayachee et al. reported zero antimicrobial activity of *Milicia excelsa* extract against *E. coli* [126]. The roles of secondary metabolites are relatively straightforward; for instance, they participate in general protective roles (antioxidant, free radical scavenging, UV light absorbing, and antiproliferative agents) and protect the plant from herbivorous animals (grazing) including different pathogenic microorganisms such as bacteria, fungi, and viruses. They also manage interplant relationships, acting as allelopathic defenders of the plant's growing space against competitor plants [127].

Several phytochemicals have already been identified using GC-MS or HPLC-MS for *Ceiba pentandra* [109, 111], for *Myrianthus arboreus* [96, 128] and for *Milicia excelsa* [129]. These bioactive components are known to exhibit medicinal property [130]. In addition, in the current study, a positive correlation was observed between total phenolic, flavonoid, proanthocyanidin and tannin content with antimicrobial and antioxidant activity. The antioxidant activity of an extract or compound is often associated with their redox proprieties, which allow them to act as reducing agents [131]. Several other studies have also reported positive correlations between plant extracts secondary metabolites such as phenolic [132], flavonoid [133], proanthocyanidin [134], tannin [135] and antimicrobial activity.

Biodiversity contributes significantly towards human livelihood and development and thus plays a predominant role in the well-being of the global population [136]. Valorization of plants with medicinal value is a world challenge that meets the objectives of biodiversity conservation [8, 137]. The latter could involve the study of phytochemistry, pharmacological properties, antioxidant and antimicrobial activities of plants consumed by non-human animals, including

great apes such as western lowland gorilla [2, 138]. Additionally, new drug discovery from natural sources involve a multifaceted approach combining botanical, phytochemical, biological, and molecular techniques [139, 140]. These bioprospecting practices have implications for medicine, environment, economy, public health, and culture [141]. Unfortunately, the potential benefits of plant-based medicines have led to unscientific exploitation of natural resources, a phenomenon that is being observed globally. The decline in biodiversity and loss due to species extinction is largely the result of the rise in the global population, rapid and sometimes unplanned industrialization, indiscriminate deforestation, overexploitation of natural resources, illegal trade, pollution, and finally global climate change [141, 142]. Therefore, it is of utmost importance that biodiversity is preserved, to provide future structural diversity and compounds for the sustainable development of human civilization [139, 143]. This becomes even more important for low and middle-income nations, where well-planned bioprospecting coupled with non-destructive commercialization could help in the conservation of biodiversity, ultimately benefiting humankind in the long run [139, 143].

Conclusion

This study investigated the antioxidant and antimicrobial activity of *Ceiba pentandra*, *Myrianthus arboreus*, *Ficus* ssp and *Milicia excelsa* BCE. These plants are consumed by western lowland gorilla living in MDNP and used in traditional medicine by Gabonese healers. All plant BCEs studied showed antioxidant and antimicrobial properties. The results of our study suggest the importance of these four for use in pharmacy and phytotherapy. These results show that the BCE of these plants may be used as an effective treatment for diseases caused by free radicals and diseases caused by antimicrobial-resistant bacterial strains. Then, all this founding comfort the self-medication hypothesis of animals, including great apes such as western lowland gorilla living in MDNP, who use plants items to heal themselves and thus control pathogenic microorganisms such as MDR *E. coli* (DECs) that cause persistent diarrheal diseases in humans and NHPs. The regular consumption of these medicinal plants in gorilla may play a preventative role in health maintenance. In addition, our results provide scientific and analytical evidence to justify the use of these plants in traditional African pharmacopoeia by healers against various diseases, which could be interesting as a promising new source for new drug discovery in the pharmaceutical, cosmetic and food industries. Thus, based on our results and recent literature, the application of new therapeutic protocols against infectious diseases of bacterial origin, based on the use of natural bark crude extracts such as *Ceiba pentandra*, *Myrianthus arboreus*, *Ficus* ssp and *Milicia excelsa* would be a real possibility and

should be studied further. Moreover, these bark crude extracts should be studied *in vivo* conditions for better understanding of their safety, efficacy, properties, and activities. Isolation and identification of bioactive compounds present in the BCEs of the plants, so that they can be exploited in the pharmaceutical, cosmetic, and food industries, as substitute for the commercially available synthetic drugs that may have a large number of side effects, is necessary.

Author Contributions: Formal analysis, L.E.D.O.Y. and C.S.O.; investigation, L.E.D.O.Y., R.O. and E.F.A.-O.; methodology, L.E.D.O.Y., R.O., C.S.O. and H.B.; project administration, L.E.D.O.Y., R.O., and C.S.O.; supervision, L.E.D.O.Y., R.O., C.S.O.; E.F.A.-O., I. and S.G.; Resource, L.C.O.E., J.P.O.; G.-R.N.A. and J.M.S.; writing—original draft, L.E.D.O.Y.; writing—review and editing, L.E.D.O.Y., R.O.; C.S.O and J.M.S. All authors have read and agreed to the published version of the manuscript.

Funding: This research received no external funding.

Institutional Review Board Statement: Not applicable.

Informed Consent Statement: Not applicable.

Data Availability Statement: Not applicable.

Acknowledgments:

The authors would like to acknowledge Doctoral School of Tropical Infectious Diseases of Franceville (EDR), Interdisciplinary Center for Medical Research of Franceville (CIRMF) and University of Sciences and Technology of Masuku (USTM) of Franceville. We also acknowledge Jean Bernard LEKANA-DOUKI and Jacques LEBIBI, Managing, Etienne François AKOMO-OKOUE, for providing his logistically and technically support for sampling in Doussala Station, Christophe NGOKOMAKA, mission vehicle driver and Peter MOMBO, Doussala station trackers team leader and all his collaborators.

Conflicts of Interest: Authors declare no conflict of interest.

References

1. Giannenas, I., et al., *The history of herbs, medicinal and aromatic plants, and their extracts: Past, current situation and future perspectives*, in *Feed additives*. 2020, Elsevier. p. 1-18.
2. Domínguez-Martín, E.M., et al., *Zoopharmacology: a way to discover new cancer treatments.* Biomolecules, 2020. **10**(6): p. 817.
3. Ahoua, A.R.C., et al., *Antimicrobial potential of 27 plants consumed by chimpanzees (Pan troglodytes verus Blumenbach) in Ivory Coast.* BMC Complementary and Alternative Medicine, 2015. **15**(1): p. 1-12.

4. Sivakanthan, S., et al., *Antioxidant and antimicrobial applications of biopolymers: A review.* Food Research International, 2020. **136**: p. 109327.
5. Bordean, M.-E., et al., *Antibacterial and Phytochemical Screening of Artemisia Species.* Antioxidants, 2023. **12**(3): p. 596.
6. Kandyliari, A., et al., *Development of Dairy Products Fortified with Plant Extracts: Antioxidant and Phenolic Content Characterization.* Antioxidants, 2023. **12**(2): p. 500.
7. Sharifi-Rad, M., et al., *Lifestyle, oxidative stress, and antioxidants: Back and forth in the pathophysiology of chronic diseases.* Frontiers in physiology, 2020. **11**: p. 694.
8. Llauradó Maury, G., et al., *Antioxidants in plants: A valorization potential emphasizing the need for the conservation of plant biodiversity in Cuba.* Antioxidants, 2020. **9**(11): p. 1048.
9. Shaikh, S., et al., *Mechanistic insights into the antimicrobial actions of metallic nanoparticles and their implications for multidrug resistance.* International journal of molecular sciences, 2019. **20**(10): p. 2468.
10. Raut, P.K., et al., *Growth of breast cancer cells by leptin is mediated via activation of the inflammasome: Critical roles of estrogen receptor signaling and reactive oxygen species production.* Biochemical pharmacology, 2019. **161**: p. 73-88.
11. Panieri, E. and M. Santoro, *ROS homeostasis and metabolism: a dangerous liason in cancer cells.* Cell death & disease, 2016. **7**(6): p. e2253-e2253.
12. Ramalingam, A.J., *History of antibiotics and evolution of resistance.* Research J. Pharm. and Tech, 2015. **8**(12): p. 1719-1724.
13. Talebi Bezmin Abadi, A., et al., *World Health Organization report: current crisis of antibiotic resistance.* BioNanoScience, 2019. **9**: p. 778-788.
14. Behzadi, P., et al., *The role of gram-negative bacteria in urinary tract infections: current concepts and therapeutic options.* Advances in Microbiology, Infectious Diseases and Public Health, 2020: p. 35-69.
15. Kahn, R., D. Clouser, and J. Richt, *Emerging infections: a tribute to the one medicine, one health concept.* Zoonoses and Public Health, 2009. **56**(6-7): p. 407-428.
16. Bailey, C. and K. Mansfield, *Emerging and reemerging infectious diseases of nonhuman primates in the laboratory setting.* Veterinary pathology, 2010. **47**(3): p. 462-481.
17. Estrada, A., et al., *Impending extinction crisis of the world's primates: Why primates matter.* Science advances, 2017. **3**(1): p. e1600946.
18. Dunay, E., et al., *Pathogen transmission from humans to great apes is a growing threat to primate conservation.* EcoHealth, 2018. **15**: p. 148-162.
19. Rabinowitz, P., M. Scotch, and L. Conti, *Human and animal sentinels for shared health risks.* Veterinaria Italiana, 2009. **45**(1): p. 23.
20. Quammen, D., *Ebola: the natural and human history of a deadly virus*. 2014: WW Norton & Company.
21. Köndgen, S., et al., *Pandemic human viruses cause decline of endangered great apes.* Current Biology, 2008. **18**(4): p. 260-264.
22. Calvignac-Spencer, S., et al., *Wild great apes as sentinels and sources of infectious disease.* Clinical Microbiology and Infection, 2012. **18**(6): p. 521-527.
23. Carne, C., et al., *The risk of disease to great apes: simulating disease spread in orang-utan (Pongo pygmaeus wurmbii) and chimpanzee (Pan troglodytes schweinfurthii) association networks.* PloS one, 2014. **9**(4): p. e95039.
24. Zimmerman, D.M., et al., *Great ape health watch: Enhancing surveillance for emerging infectious diseases in great apes.* American journal of primatology, 2022. **84**(4-5): p. e23379.
25. Vila, J., J. Moreno-Morales, and C. Ballesté-Delpierre, *Current landscape in the discovery of novel antibacterial agents.* Clinical Microbiology and Infection, 2020. **26**(5): p. 596-603.
26. Iskandar, K., et al., *Antibiotic Discovery and Resistance: The Chase and the Race.* Antibiotics, 2022. **11**(2): p. 182.
27. Rasool, A., et al., *Medicinal plants: Role, distribution and future.* J Pharmacogn Phytochem, 2020. **9**(2): p. 2111-2114.

28. Christaki, S., et al., *Recent advances in plant essential oils and extracts: Delivery systems and potential uses as preservatives and antioxidants in cheese.* Trends in Food Science & Technology, 2021. **116**: p. 264-278.
29. Álvaro, M.M., et al., *The origins of zoopharmacognosy: How humans learned about self-medication from animals.* Int J Appl Res, 2019. **5**: p. 73-79.
30. Takenoshita, Y., C. Ando, and J. Yamagiwa, *Fruit phenology of the great ape habitat in the Moukalaba-Doudou National Park, Gabon.* African Study Monographs. Supplementary Issue., 2008. **39**: p. 23-39.
31. Nguelet, F.L.M., et al., *Etude de la relation entre l'abondance des grands mammifères frugivores et celle des fruits dans le Parc National de Moukalaba-Doudou, Gabon.* International Journal of Biological and Chemical Sciences, 2016. **10**(5): p. 1969-1982.
32. Yamagiwa, J. and A.K. Basabose, *Diet and seasonal changes in sympatric gorillas and chimpanzees at Kahuzi–Biega National Park.* Primates, 2006. **47**(1): p. 74-90.
33. Bye, Z.L., et al., *What Role Do Plant-Based Diets Play in Supporting the Optimal Health and Well-being of Canadians? A Scoping Review.* Advances in Nutrition, 2021. **12**(6): p. 2132-2146.
34. Huffman, M.A., *Self-Medicative Behavior in the African Great Apes: An Evolutionary Perspective into the Origins of Human Traditional Medicine: In addition to giving us a deeper understanding of our closest living relatives, the study of great ape self-medication provides a window into the origins of herbal medicine use by humans and promises to provide new insights into ways of treating parasite infections and other serious diseases.* BioScience, 2001. **51**(8): p. 651-661.
35. Cousins, D. and M.A. Huffman, *Medicinal properties in the diet of gorillas: an ethno-pharmacological evaluation.* African study monographs, 2002. **23**(2): p. 65-89.
36. Maloueki, U., et al., *Propriétés ethnomédicinales et pharmacologiques des plantes consommées par les bonobos (Pan paniscus) à Bolobo, République Démocratique du Congo: végétation herbacée terrestre*. 2015, Ethnopharmacologia.
37. Obiang, C.S., et al., *Antimicrobial, antioxidant, anti-inflammatory and cytotoxic study of extracts of Guibourtia tessmanii (harms) J. Léonard from Gabon.* Clinical Phytoscience, 2021. **7**(1): p. 1-8.
38. Winding, W., *One health: Pulling animal health and public health together.* Federafion of Veterinarians of Europe, 2007.
39. Afzal, S., et al., *A Comparative Analysis of Antimicrobial, Antibiofilm and Antioxidant Activity of Silver Nanoparticles Synthesized from Erythrina Suberosa Roxb. and Ceiba Pentandra.* Journal of Oleo Science, 2022. **71**(4): p. 523-533.
40. Kasangana, P.B., et al., *Root bark extracts of Myrianthus arboreus P. Beauv.(Cecropiaceae) exhibit anti-diabetic potential by modulating hepatocyte glucose homeostasis.* Journal of ethnopharmacology, 2018. **211**: p. 117-125.
41. Simsek, E., D. Kilic, and O. Caliskan, *Phenotypic variation of fig genotypes (Ficus carica L.) in the eastern Mediterranean of Turkey.* Genetika, 2020. **52**(3): p. 957-972.
42. Oke-Altuntas, F., et al., *Bioactivity evaluation of cudraxanthone I, neocyclomorusin and (9βh)-3β-acetoxylanosta-7, 24-diene isolated from Milicia excelsa Welw. CC Berg (Moraceae).* Medicinal Chemistry Research, 2016. **25**(10): p. 2250-2257.
43. Obiang, C.S., et al., *Ethnopharmacological Study and Antibacterial Activities of Some Plants Used in Traditional Medicine for the Treatment of Diarrheal Diseases in Gabon.* Traditional and Integrative Medicine, 2022: p. 386-401.
44. Wubetu, M., T. Abula, and G. Dejenu, *Ethnopharmacologic survey of medicinal plants used to treat human diseases by traditional medical practitioners in Dega Damot district, Amhara, Northwestern Ethiopia.* BMC research notes, 2017. **10**(1): p. 1-13.
45. Mohammedi, H., et al., *The effect of solvents and extraction procedure on the recovery of phenolic compounds and the antioxidant capacity of Algerian Bassia muricata L. extracts.* Chemistry Journal of Moldova, 2019. **14**(2): p. 79-89.

46. Aiyegoro, O.A. and A.I. Okoh, *Preliminary phytochemical screening and in vitro antioxidant activities of the aqueous extract of Helichrysum longifolium DC.* BMC complementary and alternative medicine, 2010. **10**(1): p. 1-8.
47. Sadeq, O., et al., *Phytochemical screening, antioxidant and antibacterial activities of pollen extracts from micromeria fruticosa, achillea fragrantissima, and phoenix dactylifera.* Plants, 2021. **10**(4): p. 676.
48. Hossain, M.A., et al., *Study of total phenol, flavonoids contents and phytochemical screening of various leaves crude extracts of locally grown Thymus vulgaris.* Asian Pacific journal of tropical biomedicine, 2013. **3**(9): p. 705-710.
49. Do, Q.D., et al., *Effect of extraction solvent on total phenol content, total flavonoid content, and antioxidant activity of Limnophila aromatica.* Journal of food and drug analysis, 2014. **22**(3): p. 296-302.
50. Sima-Obiang, C., et al., *Phytochemical screening, phenolic compounds content, antioxidant, anti-inflammatory, and antimicrobial properties of Pachylobus balsamifera Guillaum from Gabon.* Phytothérapie, 2018. **16**(S1): p. S65-S73.
51. Ngoua-Meye-Misso, R.-L., et al., *Phytochemical screening, antioxidant, anti-inflammatory and antiangiogenic activities of Lophira procera a. Chev.(Ochnaceae) medicinal plant from Gabon.* Egyptian Journal of Basic and Applied Sciences, 2018. **5**(1): p. 80-86.
52. Scherer, R. and H.T. Godoy, *Antioxidant activity index (AAI) by the 2, 2-diphenyl-1-picrylhydrazyl method.* Food chemistry, 2009. **112**(3): p. 654-658.
53. Prieto, P., M. Pineda, and M. Aguilar, *Spectrophotometric quantitation of antioxidant capacity through the formation of a phosphomolybdenum complex: specific application to the determination of vitamin E.* Analytical biochemistry, 1999. **269**(2): p. 337-341.
54. Kubola, J. and S. Siriamornpun, *Phenolic contents and antioxidant activities of bitter gourd (Momordica charantia L.) leaf, stem and fruit fraction extracts in vitro.* Food chemistry, 2008. **110**(4): p. 881-890.
55. Prieto, M., et al., *β-Carotene assay revisited. Application to characterize and quantify antioxidant and prooxidant activities in a microplate.* Journal of Agricultural and Food Chemistry, 2012. **60**(36): p. 8983-8993.
56. Oyaba Yinda, L.E.D., et al., *Phylogenetic Groups, Pathotypes and Antimicrobial Resistance of Escherichia coli Isolated from Western Lowland Gorilla Faeces (Gorilla gorilla gorilla) of Moukalaba-Doudou National Park (MDNP).* Pathogens, 2022. **11**(10): p. 1082.
57. Falciglia, M.D., et al., *In Vitro Antimicrobial Activity Evaluation of a Novel Fitostimoline® Plus Spray Formulation.* International Journal of Microbiology, 2021. **2021**.
58. Wayne, P., *Clinical and laboratory standards institute. Performance standards for antimicrobial susceptibility testing.* 2011.
59. Imtara, H., Y. Elamine, and B. Lyoussi, *Honey antibacterial effect boosting using Origanum vulgare L. essential oil.* Evidence-Based Complementary and Alternative Medicine, 2018. **2018**.
60. Bamunusinghage, N.P., et al., *Antimicrobial Resistance Patterns of Fecal Escherichia coli in Wildlife, Urban Wildlife, and Livestock in the Eastern Region of Sri Lanka, and Differences between Carnivores, Omnivores, and Herbivores.* The Journal of Wildlife Diseases, 2022. **58**(2): p. 380-383.
61. Ibrahim, M., N. Bilal, and M. Hamid, *Increased multi-drug resistant Escherichia coli from hospitals in Khartoum state, Sudan.* African health sciences, 2012. **12**(3): p. 368-375.
62. Peterson, L.R. and S.M. Collins, *Bactericidal testing for infectious disease therapy: the why and how to measure if we kill the bugs.* Clinical Microbiology Newsletter, 2000. **22**(20): p. 153-157.
63. El-Ashmawy, I.M., N.A. Al-Wabel, and A.E. Bayad, *Achillea fragrantissima, rich in flavonoids and tannins, potentiates the activity of diminazine aceturate against Trypanosoma evansi in rats.* Asian Pacific Journal of Tropical Medicine, 2016. **9**(3): p. 228-234.
64. Abiola, A.L., et al., *Spectroscopic analysis and anti-inflammatory effects of Milicia excelsa (Moraceae) leaf and fractions.* GSC Biological and Pharmaceutical Sciences, 2019. **6**(3): p. -.

65. Murugesu, S., J. Selamat, and V. Perumal, *Phytochemistry, pharmacological properties, and recent applications of Ficus benghalensis and Ficus religiosa.* Plants, 2021. **10**(12): p. 2749.
66. Tijani, R.O., et al., *Myrianthus arboreus P. Beauv improves insulin sensitivity in high fat diet-induced obese mice by reducing inflammatory pathways activation.* Journal of Ethnopharmacology, 2022. **282**: p. 114651.
67. Njokuocha, R.C. and A. Ewinike, *Antibacterial and phytochemical properties of crude leaf extract of Moringa oleifera Lam., Pterocarpus santalinoides L'Herit DC and Ceiba pentandra L. on some clinical bacteria isolates in Nigeria.* Journal of Complementary and Alternative Medical Research, 2020. **10**(4): p. 1-15.
68. Szopa, A., R. Ekiert, and H. Ekiert, *Current knowledge of Schisandra chinensis (Turcz.) Baill.(Chinese magnolia vine) as a medicinal plant species: a review on the bioactive components, pharmacological properties, analytical and biotechnological studies.* Phytochemistry Reviews, 2017. **16**(2): p. 195-218.
69. Fowler, M.W., *Plants, medicines and man.* Journal of the Science of Food and Agriculture, 2006. **86**(12): p. 1797-1804.
70. Ngwa, W., et al., *Potential of flavonoid-inspired phytomedicines against COVID-19.* Molecules, 2020. **25**(11): p. 2707.
71. Greene, A.M., et al., *Asian elephant self-medication as a source of ethnoveterinary knowledge among Karen mahouts in northern Thailand.* Journal of ethnopharmacology, 2020. **259**: p. 112823.
72. Krief, S., et al., *Bioactive properties of plant species ingested by chimpanzees (Pan troglodytes schweinfurthii) in the Kibale National Park, Uganda.* American Journal of Primatology: Official Journal of the American Society of Primatologists, 2006. **68**(1): p. 51-71.
73. Masi, S., et al., *Unusual feeding behavior in wild great apes, a window to understand origins of self-medication in humans: role of sociality and physiology on learning process.* Physiology & behavior, 2012. **105**(2): p. 337-349.
74. Patwardhan, B., et al., *Ayurveda and traditional Chinese medicine: a comparative overview.* Evidence-based complementary and alternative medicine, 2005. **2**(4): p. 465-473.
75. Anosike, C., et al., *Phytochemical screening and antimicrobial activity of the petroleum ether, methanol and ethanol extracts of Ceiba pentandra stem bark.* Journal of Medicinal Plants Research, 2012. **6**(46): p. 5743-5747.
76. Osuntokun, O., et al., *Assessment of antimicrobial and phytochemical properties of crude leaf and bark extracts of Ceiba pentandra on selected clinical isolates found in Nigerian teaching hospital.* Journal of Bacteriology and Mycology Open Access, 2017. **4**(1): p. 17-23.
77. Olonode, E.T., A.O. Aderibigbe, and A.G. Bakre, *Anti-nociceptive activity of the crude extract of Myrianthus arboreus P. Beauv (Cecropiaceae) in mice.* Journal of ethnopharmacology, 2015. **171**: p. 94-98.
78. Mvondo, M.A., et al., *The leaf aqueous extract of Myrianthus arboreus P. Beauv.(Cecropiaceae) improved letrozole-induced polycystic ovarian syndrome associated conditions and infertility in female Wistar rats.* BMC Complementary Medicine and Therapies, 2020. **20**: p. 1-13.
79. Abiola, A.L., et al., *Acute Toxicity and Sedative-hypnotic Effects of Ethanol Stem Bark Extract and Fractions of Milicia excelsa (Moraceae) in Mice.* Adv. Pharmacol. Pharm., 2020. **8**: p. 11-18.
80. Parulekar, G., *Antibacterial and phytochemical analysis of Ceiba pentandra (L.) seed extracts.* Journal of Pharmacognosy and Phytochemistry, 2017. **6**(3): p. 586-589.
81. Biapa, P., et al., *Phytochemical studies and antioxidant properties of four medicinal plants used in Cameroon.* African Journal of Traditional, Complementary and Alternative Medicines, 2007. **4**(4): p. 495-500.
82. Agyare, C., et al., *Wound healing and anti-infective properties of Myrianthus arboreus and Alchornea cordifolia.* Med. Chem, 2014. **4**(7): p. 533-539.

83. Owusu, E., et al., *Antimicrobial activity of selected medicinal plants from a sub-Saharan African country against bacterial pathogens from post-operative wound infections.* Medical Sciences, 2021. **9**(2): p. 23.
84. Fankam, A.G., et al., *Antioxidant and antifungal activities of Myrianthus arboreus P. Beauv.(Moraceae), Allanblackia gabonensis Pellegr.(Clusiaceae) and three other Cameroonian medicinal plants.*
85. Eissa, S., et al., *Molecular Genetic Studies on (Ficus Palmata & Ficus Carica Subsp Rupestris.) Under the Effect of Gamma Radiation by Tissue Culture Techniques.* Al-Azhar Journal of Agricultural Research, 2021. **46**(2): p. 128-138.
86. Lazreg-Aref, H., et al., *Chemical composition and antibacterial activity of a hexane extract of Tunisian caprifig latex from the unripe fruit of Ficus carica.* Pharmaceutical Biology, 2012. **50**(4): p. 407-412.
87. Paşayeva, L., B. Özalp, and H. Fatullayev, *Potential enzyme inhibitory properties of extracts and fractions from fruit latex of Ficus carica-based on inhibition of α-amylase and α-glucosidase.* Journal of Food Measurement and Characterization, 2020. **14**(5): p. 2819-2827.
88. Toma, C.C., et al., *Ficus carica SSP Dottato Buds by Intercropping Different Species: Metabolites, Antioxidant Activity and Endogenous Plant Hormones (IAA, ABA).* Rev. Chim, 2017. **68**: p. 1628-1631.
89. Udegbunam, S., et al., *Evaluation of herbal ointment formulation of Milicia excelsa (Welw) CC berg for wound healing.* African journal of biotechnology, 2013. **12**(21).
90. Ayepola, O.O., A.F. Samson, and O. Onile-Ere, *In vitro Antioxidant and Anti-staphylococcal Activity of Bixa orellana Linn. and Milicia excelsa Welw.*
91. Huang, Y.-H., et al., *The traditional uses, secondary metabolites, and pharmacology of Eleutherococcus species.* Phytochemistry Reviews, 2022. **21**(4): p. 1081-1184.
92. ElNaggar, M.H., et al., *The old world salsola as a source of valuable secondary metabolites endowed with diverse pharmacological activities: a review.* Journal of Enzyme Inhibition and Medicinal Chemistry, 2022. **37**(1): p. 2036-2062.
93. Rigat, M., et al., *Traditional and alternative natural therapeutic products used in the treatment of respiratory tract infectious diseases in the eastern Catalan Pyrenees (Iberian Peninsula).* Journal of Ethnopharmacology, 2013. **148**(2): p. 411-422.
94. Hajdu, Z. and J. Hohmann, *An ethnopharmacological survey of the traditional medicine utilized in the community of Porvenir, Bajo Paraguá Indian Reservation, Bolivia.* Journal of ethnopharmacology, 2012. **139**(3): p. 838-857.
95. Harley, B.K., et al., *Flavanols and triterpenoids from Myrianthus arboreus ameliorate hyperglycaemia in streptozotocin-induced diabetic rats possibly via glucose uptake enhancement and α-amylase inhibition.* Biomedicine & Pharmacotherapy, 2020. **132**: p. 110847.
96. Kasangana, P.B., P.S. Haddad, and T. Stevanovic, *Study of polyphenol content and antioxidant capacity of Myrianthus arboreus (Cecropiaceae) root bark extracts.* Antioxidants, 2015. **4**(2): p. 410-426.
97. Agyare, C., et al., *Wound healing and anti-infective properties of Myrianthus arboreus and Alchornea cordifolia.* 2014.
98. Alzahrani, F.O., et al., *Total phenol, flavonoid, and individual phenolic acid contents and antioxidant and cytotoxic activities of methanol extracts from Ficus cordata ssp. salicifolia.* Canadian Journal of Plant Science, 2021. **101**(4): p. 517-524.
99. Sumi, S.A., et al., *Investigation of the key pharmacological activities of Ficus racemosa and analysis of its major bioactive polyphenols by HPLC-DAD.* Evidence-Based Complementary and Alternative Medicine, 2016. **2016**.
100. Adebayo, M.A., et al., *Evaluation of anti-diarrheal activity of methanol root bark extract of Milicia excelsa (Welw) C. C Berg (Moraceae) in rats.* Drug Research, 2019. **69**(08): p. 439-444.
101. Tungmunnithum, D., et al., *Flavonoids and other phenolic compounds from medicinal plants for pharmaceutical and medical aspects: An overview.* Medicines, 2018. **5**(3): p. 93.

102. Dirar, A., et al., *Effects of extraction solvents on total phenolic and flavonoid contents and biological activities of extracts from Sudanese medicinal plants.* South African Journal of Botany, 2019. **120**: p. 261-267.

103. Mahomoodally, M.F., *Traditional medicines in Africa: an appraisal of ten potent African medicinal plants.* Evidence-based complementary and alternative medicine, 2013. **2013**.

104. Navarro, M., et al., *Proanthocyanidin Characterization, Antioxidant and Cytotoxic Activities of Three Plants Commonly Used in Traditional Medicine in Costa Rica: Petiveria alliaceae L., Phyllanthus niruri L. and Senna reticulata Willd.* Plants, 2017. **6**(4): p. 50.

105. Moncayo, S., et al., *Preliminary phytochemical screening for antioxidant activity and content of phenols and flavonoids of 18 species of plants native to western Ecuador.* Trends in Phytochemical Research, 2021. **5**(2): p. 93-104.

106. Kusuma, I.W., E.T. Arung, and Y.-u. Kim, *Antimicrobial and antioxidant properties of medicinal plants used by the Bentian tribe from Indonesia.* Food Science and Human Wellness, 2014. **3**(3-4): p. 191-196.

107. Ilecholubo, A.P., A.C. Okpachi, and E.D. Ocholi, *Phytochemical constituents and Antioxidant properties of Aqueous leaf extract of Myrianthus arboreus.* Int. J. Biomed. Sci. Eng., 2017. **5**(1): p. 9.

108. Konan, Y., et al., *Antioxidant activity and total phenolic content of nine plants from Côte d'Ivoire (West Africa).* Journal of Applied Pharmaceutical Science, 2014. **4**(8): p. 036-041.

109. Bhavani, R., E. Bhuvaneswari, and S. Rajeshkumar, *Antibacterial and Antioxidant activity of Ethanolic extract of Ceiba pentandra leaves and its Phytochemicals Analysis using GC-MS.* Research Journal of Pharmacy and Technology, 2016. **9**(11): p. 1922-1926.

110. Fofié, C.K., et al., *Hypoglycemic properties of the aqueous extract from the stem bark of Ceiba pentandra in dexamethasone-induced insulin resistant rats.* Evidence-Based Complementary and Alternative Medicine, 2018. **2018**.

111. Nguelefack, T.B., et al., *Multimodal α-glucosidase and α-amylase inhibition and antioxidant effect of the aqueous and methanol extracts from the trunk bark of Ceiba pentandra.* BioMed research international, 2020. **2020**.

112. Aji, A., et al., *Biosynthesis of gold nanoparticles using Kapok (Ceiba pentandra) leaf aqueous extract and investigating their antioxidant activity.* Journal of Molecular Structure, 2022. **1270**: p. 133906.

113. Ekere, N., T. Okparanozie, and M. Agbo, *Effects of solvents on the in-vitro antioxidant activity of Dennettia tripetala G. Baker and Milicia excelsa (Welw.) C. Berg root extracts.* Iranian Journal of Health Sciences, 2018.

114. Akinpelu, L.A., et al., *Phytochemical estimations and antihypoxic effect of ethanol leaf extract of Milicia excelsa (Moraceae) in mice.* GSC Biological and Pharmaceutical Sciences, 2020. **10**(2): p. 024-029.

115. Latayada, F.S. and M.M. Uy, *Screening of the antioxidant properties of the leaf extracts of Philippine medicinal plants Ficus nota (Blanco) Merr., Metroxylon sagu Rottb., Mussaenda philippica A. Rich., Inocarpus fagifer, and Cinnamomum mercadoi Vidal.* Bull Environ Pharmacol Life Sci, 2016. **5**(3): p. 18-24.

116. Abdel-Aty, A.M., et al., *Ficus carica, Ficus sycomorus and Euphorbia tirucalli latex extracts: Phytochemical screening, antioxidant and cytotoxic properties.* Biocatalysis and Agricultural Biotechnology, 2019. **20**: p. 101199.

117. Yadav, Y.C., et al., *In-vitro antioxidant activity of methanolic extraction of Ficus Benghalensis L. latex.* Pharmacologyonline, 2011. **1**: p. 140-148.

118. Sinha, S., et al., *Antioxidant and choline esterase inhibitory activity of phenolic rich extracts from Bombax ceiba l. flowers.* Free Radicals and Antioxidants, 2018. **8**(2): p. 135-140.

119. Niki, E., *Assessment of antioxidant capacity in vitro and in vivo.* Free Radical Biology and Medicine, 2010. **49**(4): p. 503-515.

120. Da Pozzo, E., et al., *Antioxidant and antisenescence effects of bergamot juice.* Oxidative medicine and cellular longevity, 2018. **2018**.

121. Santos-Sánchez, N.F., et al., *Antioxidant compounds and their antioxidant mechanism.* Antioxidants, 2019. **10**: p. 1-29.
122. Yuan, G., et al., *Antibacterial activity and mechanism of plant flavonoids to gram-positive bacteria predicted from their lipophilicities.* Scientific reports, 2021. **11**(1): p. 10471.
123. Kuete, V., et al., *Antibacterial activities of the extracts, fractions and compounds from Dioscorea bulbifera.* BMC Complementary and Alternative Medicine, 2012. **12**(1): p. 1-8.
124. Tkachenko, H., et al., *Preliminary in vitro screening of the antibacterial activity of leaf extracts from various Ficus species (Moraceae) against Yersinia ruckeri.* Fisheries & Aquatic Life, 2019. **27**(1).
125. Biwôlé, J.J.E., et al., *Iroko wood (Milicia excelsa CC berg), a good candidate for high-speed rotation-induced wood dowel welding: An assessment of its welding potential and the water resistance of its welded joints.* International Journal of Adhesion and Adhesives, 2023: p. 103360.
126. Padayachee, T. and B. Odhav, *Antimicrobial activity of plant phenols from Chlorophora excelsa and Virgilia oroboides.* African Journal of Biotechnology, 2013. **12**(17).
127. Tahara, S., *A journey of twenty-five years through the ecological biochemistry of flavonoids.* Bioscience, biotechnology, and biochemistry, 2007. **71**(6): p. 1387-1404.
128. García-Pérez, M.-E., P.-B. Kasangana, and T. Stevanovic, *Bioactive Molecules from Myrianthus arboreus, Acer rubrum, and Picea mariana Forest Resources.* Molecules, 2023. **28**(5): p. 2045.
129. Oke-Altuntas, F., et al., *Bioactivity evaluation of cudraxanthone I, neocyclomorusin and (9βh)-3β-acetoxylanosta-7, 24-diene isolated from Milicia excelsa Welw. CC Berg (Moraceae).* Medicinal Chemistry Research, 2016. **25**: p. 2250-2257.
130. Olukemi, O.A., et al., *The use of selected Nigerian natural products in management of environmentally induced free radical skin damage.* Pakistan Journal of Biological Sciences, 2005. **8**(8): p. 1074-1077.
131. Diouf, P.N., T. Stevanovic, and A. Cloutier, *Antioxidant properties and polyphenol contents of trembling aspen bark extracts.* Wood Science and Technology, 2009. **43**: p. 457-470.
132. Weerakkody, N.S., et al., *In vitro antimicrobial activity of less-utilized spice and herb extracts against selected food-borne bacteria.* Food Control, 2010. **21**(10): p. 1408-1414.
133. Tian, Y., et al., *Antioxidative and antibacterial activities of aqueous ethanol extracts of berries, leaves, and branches of berry plants.* Food research international, 2018. **106**: p. 291-303.
134. Navarro-Hoyos, M., et al., *Proanthocyanidin characterization and bioactivity of extracts from different parts of Uncaria tomentosa L.(cat's claw).* Antioxidants, 2017. **6**(1): p. 12.
135. Yahia, Y., et al., *Bioactive compounds, antioxidant and antimicrobial activities of extracts from different plant parts of two Ziziphus Mill. species.* PloS one, 2020. **15**(5): p. e0232599.
136. Sandifer, P.A., A.E. Sutton-Grier, and B.P. Ward, *Exploring connections among nature, biodiversity, ecosystem services, and human health and well-being: Opportunities to enhance health and biodiversity conservation.* Ecosystem services, 2015. **12**: p. 1-15.
137. Niesenbaum, R.A., *The integration of conservation, biodiversity, and sustainability*. 2019, MDPI. p. 4676.
138. Krishnaswamy, S., *Bioprospecting, botany, biodiversity, and their impact on botanical drug development.* Botanical Drug Products: Recent Developments and Market Trends, 2018. **39**.
139. Sen, T. and S.K. Samanta, *Medicinal plants, human health and biodiversity: a broad review.* Biotechnological applications of biodiversity, 2014: p. 59-110.
140. Ozturk, M. and K.R. Hakeem, *Plant and Human Health, Volume 2: Phytochemistry and Molecular Aspects*. 2019: Springer.
141. Alves, R. and H.N. Alves, *The faunal drugstore: Animal-based remedies used in traditional medicines in Latin America.* Journal of ethnobiology and ethnomedicine, 2011. **7**(1): p. 1-43.
142. Mukherjee, J., *Biotechnological Applications of Biodiversity*. 2015: Springer.
143. Shukla, V. and N. Kumar, *Environmental Concerns and Sustainable Development*. 2020: Springer.
