# Supplementary material for: Antibacterial and antioxidant activities of plants consumed by western lowland gorilla (Gorilla gorilla gorilla) in Gabon
Source: PLoS One. 2024 Sep 11;19(9):e0306957. doi: 10.1371/journal.pone.0306957 (PMC11389915; doi:10.1371/journal.pone.0306957)
Supplement: Attachment — Submitted filename: One Plos.docx [file pone.0306957.s004.docx]

The authors have done great research. However, more information and or explanation is required

1. Please include the references in line 62-63
2. Are the items listed in 67-70 going to be part of the study. If not, please revise.
3. The introduction lacks the problem statement and motivation to link the chosen species and the natural medicine. Provide more information and compelling reasons for the audience to clearly understand.
4. Please also include the hypothesis
5. In the methodology section
   a. Please state if your collection methodology has similarities with the manner in which gorillas consume. It would be interesting to understanding the rationale behind the sampling methodology or approach
   b. Fig 3 a, b; would be better visible with whole numbers
   c. Fig 4; why a different font?
6. Line 265; there is mention of other plant organs of interest which have not been stated in the introduction or methodology. Any reasons to mention these at this stage?
7. The discussion does not explain results except to relate it to other studies. Explain why certain observations as well as the reasons behind the observsations
