# Supplementary material for: Antibacterial and antioxidant activities of plants consumed by western lowland gorilla (Gorilla gorilla gorilla) in Gabon
Source: PLoS One. 2024 Sep 11;19(9):e0306957. doi: 10.1371/journal.pone.0306957 (PMC11389915; doi:10.1371/journal.pone.0306957)
Supplement: Attachment — Submitted filename: Response to reviewers.docx [file pone.0306957.s005.docx]

Journal Requirements:

1. Please ensure that your manuscript meets PLOS ONE's style requirements, including those for file naming. The PLOS ONE style templates can be found at

https://journals.plos.org/plosone/s/file?id=wjVg/PLOSOne_formatting_sample_main_body.pdf and

<https://journals.plos.org/plosone/s/file?id=ba62/PLOSOne_formatting_sample_title_authors_affiliations.pdf>

**The manuscript meets PLOS ONE style requirements, including file names. PLOS ONE style templates.**

2. In your Methods section, please provide additional information regarding the permits you obtained for the work. Please ensure you have included the full name of the authority that approved the field site access and, if no permits were required, a brief statement explaining why :

**Done**

3. Please include a complete copy of PLOS’ questionnaire on inclusivity in global research in your revised manuscript. Our policy for research in this area aims to improve transparency in the reporting of research performed outside of researchers’ own country or community. The policy applies to researchers who have travelled to a different country to conduct research, research with Indigenous populations or their lands, and research on cultural artefacts. The questionnaire can also be requested at the journal’s discretion for any other submissions, even if these conditions are not met. Please find more information on the policy and a link to download a blank copy of the questionnaire here: https://journals.plos.org/plosone/s/best-practices-in-research-reporting. Please upload a completed version of your questionnaire as Supporting Information when you resubmit your manuscript.

**Not applicable. None of the research and analysis carried out in the course of this study was carried out outside our home country.**

4. Thank you for stating the following financial disclosure:

"Non.

Les bailleurs de fonds n'ont joué aucun rôle dans la conception de l'étude, la collecte et l'analyse des données, la décision de publier ou la préparation du manuscrit."

At this time, please address the following queries:

a) Please clarify the sources of funding (financial or material support) for your study. List the grants or organizations that supported your study, including funding received from your institution.

b) State what role the funders took in the study. If the funders had no role in your study, please state: “The funders had no role in study design, data collection and analysis, decision to publish, or preparation of the manuscript.”

c) If any authors received a salary from any of your funders, please state which authors and which funders.

d) If you did not receive any funding for this study, please state: “The authors received no specific funding for this work.”

Please include your amended statements within your cover letter; we will change the online submission form on your behalf.

a) **We received no external funding for this study. The entire study was financed by the endowment granted to our research unit (field mission expenses, purchase of reagents to order, etc.), with the collaboration of partner institutions for field studies such as the Institut de Recherche en Ecologie Tropical (IRET), the Gabonese government and Total via the PID (diversified investment product), which finance and support research at CIRMF.**
b) **No, they didn't play any role in the realization of our study, except for the mission expenses and the purchase of reagents.**
c) **No author received a salary from any funder during the course of this study.**
d) **The authors received no specific funding for this work.**

5. We note that your Data Availability Statement is currently as follows: [Toutes les données pertinentes se trouvent dans le manuscrit et ses fichiers d'informations complémentaires.]

Please confirm at this time whether or not your submission contains all raw data required to replicate the results of your study. Authors must share the “minimal data set” for their submission. PLOS defines the minimal data set to consist of the data required to replicate all study findings reported in the article, as well as related metadata and methods (https://journals.plos.org/plosone/s/data-availability#loc-minimal-data-set-definition).

For example, authors should submit the following data:

- The values behind the means, standard deviations and other measures reported;

- The values used to build graphs;

- The points extracted from images for analysis.

Authors do not need to submit their entire data set if only a portion of the data was used in the reported study.

If your submission does not contain these data, please either upload them as Supporting Information files or deposit them to a stable, public repository and provide us with the relevant URLs, DOIs, or accession numbers. For a list of recommended repositories, please see https://journals.plos.org/plosone/s/recommended-repositories.

If there are ethical or legal restrictions on sharing a de-identified data set, please explain them in detail (e.g., data contain potentially sensitive information, data are owned by a third-party organization, etc.) and who has imposed them (e.g., an ethics committee). Please also provide contact information for a data access committee, ethics committee, or other institutional body to which data requests may be sent. If data are owned by a third party, please indicate how others may request data access.

**We confirm that the manuscript contains all the data required for reproducibility of the results of our study. In addition, we have provided a supplementary file as recommended by the reviewers.**

6. In the online submission form, you indicated that your data will be submitted to a repository upon acceptance. We strongly recommend all authors deposit their data before acceptance, as the process can be lengthy and hold up publication timelines. Please note that, though access restrictions are acceptable now, your entire minimal dataset will need to be made freely accessible if your manuscript is accepted for publication. This policy applies to all data except where public deposition would breach compliance with the protocol approved by your research ethics board. If you are unable to adhere to our open data policy, please kindly revise your statement to explain your reasoning and we will seek the editor's input on an exemption.

**Data will be submitted before acceptance.**

7. Please note that in order to use the direct billing option the corresponding author must be affiliated with the chosen institute. Please either amend your manuscript to change the affiliation or corresponding author, or email us at plosone@plos.org with a request to remove this option.

**Not applicable**

8. We note that Figure 1 in your submission contain map/satellite image which may be copyrighted. All PLOS content is published under the Creative Commons Attribution License (CC BY 4.0), which means that the manuscript, images, and Supporting Information files will be freely available online, and any third party is permitted to access, download, copy, distribute, and use these materials in any way, even commercially, with proper attribution. For these reasons, we cannot publish previously copyrighted maps or satellite images created using proprietary data, such as Google software (Google Maps, Street View, and Earth). For more information, see our copyright guidelines: http://journals.plos.org/plosone/s/licenses-and-copyright.

We require you to either (1) present written permission from the copyright holder to publish these figures specifically under the CC BY 4.0 license, or (2) remove the figures from your submission:

a. You may seek permission from the original copyright holder of Figure 1 to publish the content specifically under the CC BY 4.0 license.

We recommend that you contact the original copyright holder with the Content Permission Form (http://journals.plos.org/plosone/s/file?id=7c09/content-permission-form.pdf) and the following text:

“I request permission for the open-access journal PLOS ONE to publish XXX under the Creative Commons Attribution License (CCAL) CC BY 4.0 (http://creativecommons.org/licenses/by/4.0/). Please be aware that this license allows unrestricted use and distribution, even commercially, by third parties. Please reply and provide explicit written permission to publish XXX under a CC BY license and complete the attached form.”

Please upload the completed Content Permission Form or other proof of granted permissions as an ""Other"" file with your submission.

In the figure caption of the copyrighted figure, please include the following text: “Reprinted from [ref] under a CC BY license, with permission from [name of publisher], original copyright [original copyright year].”

b. If you are unable to obtain permission from the original copyright holder to publish these figures under the CC BY 4.0 license or if the copyright holder’s requirements are incompatible with the CC BY 4.0 license, please either i) remove the figure or ii) supply a replacement figure that complies with the CC BY 4.0 license. Please check copyright information on all replacement figures and update the figure caption with source information. If applicable, please specify in the figure caption text when a figure is similar but not identical to the original image and is therefore for illustrative purposes only.

The following resources for replacing copyrighted map figures may be helpful:

USGS National Map Viewer (public domain): http://viewer.nationalmap.gov/viewer/

The Gateway to Astronaut Photography of Earth (public domain): http://eol.jsc.nasa.gov/sseop/clickmap/

Maps at the CIA (public domain): https://www.cia.gov/library/publications/the-world-factbook/index.html and https://www.cia.gov/library/publications/cia-maps-publications/index.html

NASA Earth Observatory (public domain): http://earthobservatory.nasa.gov/

Landsat: http://landsat.visibleearth.nasa.gov/

USGS EROS (Earth Resources Observatory and Science (EROS) Center) (public domain): http://eros.usgs.gov/#

Natural Earth (public domain): http://www.naturalearthdata.com/

**This figure has been simplly removed from the manuscript.**

**"Response to Reviewers".**

5. Review Comments to the Author

Please use the space provided to explain your answers to the questions above. You may also include additional comments for the author, including concerns about dual publication, research ethics, or publication ethics. (Please upload your review as an attachment if it exceeds 20,000 characters)

Reviewer #1: There are some Comments for the Conclusion:

The conclusion provides valuable information about the antioxidant and antimicrobial properties of the studied plant extracts and their potential applications. However, the text could benefit from improved organization and clarity. Consider breaking down the conclusion into subsections to address different aspects more coherently, such as the results, applications, future research, and potential challenges.

**Done**

The conclusion mentions the need for in vivo studies to understand the safety and efficacy of the plant extracts. It would be valuable to specify what types of safety and efficacy assessments are required and to discuss any potential challenges or ethical considerations in conducting such studies.

**This part of the conclusion has been reworded. Indeed, when we spoke of in vivo testing, we were referring to the study of the cytotoxicity of extracts, by assessing their effects on the overall cell proliferation of cell lines.**

The conclusion suggests the need for the isolation and identification of bioactive compounds from the plant extracts. Expanding on this point by discussing potential methods for isolation and identification, as well as the significance of these compounds, would provide a more comprehensive outlook.

**This part of the conclusion has been reworded as recommended by the reviewer.**

Molecular networking (MN) is a valuable tool for revealing the metabolomes of plants, humans, microorganisms and animals [1]. It can not only annotate compounds in complex matrices based on their tandem mass spectrometry (MS/MS) characteristics but also group them into molecular families based on their spectral similarities, thus facilitating visualization of structurally identical molecules within the same chemical family and structurally different molecules distributed in different chemical families [2, 3]. This approach allows researchers to identify known compounds in order to focus on unknown compounds that may potentially be of biological interest [4].

The text mentions using these plant extracts as substitutes for commercially available synthetic drugs. It would be beneficial to elaborate on which specific diseases or conditions these extracts might address and the potential advantages over synthetic drugs, such as reduced side effects.

**Done**

While the conclusion discusses the pharmaceutical, cosmetic, and food industries as potential applications, it could delve into the specific products or treatments that might benefit from these plant extracts and how they could be incorporated.

**We plan to develop this aspect of our research, focusing on the potential represented by improved traditional medicines (ITMs).**

Reviewer #2: The manuscript “Phytochemical Screening, Antioxidant and Antibacterial 1 Activities of Four Plants Consumed by Western Lowland Gorilla (Gorilla gorilla gorilla) living in Moukalaba-Doudou National Park (MDNP).” written by YINDA et al is well prepared and could be a significance contribution in the plant taxonomy. The manuscript can be considered for publication after addressing these minor corrections.

The authors have done great research. However, more information and or explanation is required

1.	Please include the references in line 62-63

**Done**

2.	Are the items listed in 67-70 going to be part of the study. If not, please revise.

**Done**

3.	The introduction lacks the problem statement and motivation to link the chosen species and the natural medicine. Provide more information and compelling reasons for the audience to clearly understand.

**Done**

4.	Please also include the hypothesis

**Done**

5.	In the methodology section

a.	Please state if your collection methodology has similarities with the manner in which gorillas consume. It would be interesting to understanding the rationale behind the sampling methodology or approach

**Done**

**Sampling of plant barks was guided by the fact that these were not only consumed by gorillas but also used in traditional medicine by traditional practitioners healers, based on a non-invasive observation and collection methodology.**

b.	Fig 3 a, b; would be better visible with whole numbers

**Done**

c.	Fig 4; why a different font?

**Changes have been made to correct the fonts in this figure.**

6.	Line 265; there is mention of other plant organs of interest which have not been stated in the introduction or methodology. Any reasons to mention these at this stage?

**There was no particular reason to mention other interesting plant organs not mentioned in the introduction or methodology. This was done for information purposes, in order to highlight the various other organs and preparation processes (maceration, decoction, infusion, etc.) used by local populations in their traditional medicine, which gorillas cannot do.**

7.	The discussion does not explain results except to relate it to other studies. Explain why certain observations as well as the reasons behind the observsations

**Done**

Reviewer #3: -The paper is too long. It has to be shortened and stick only on essential points.

-2.3.1 extraction lines 148-149

This is not a new extraction procedure so figure 2 is without any added value. In addition Figure 2 is cited within the text as figure 1, see line 147.

**This figure has been removed from the document.**

-Line 271 Table 1. Caption: plant species……including diarrhoea…. What is the need to highlight diarrhoea here? And also diarrhoea is not listed as an indication for none of the plant species in the table.

**Diarrhoea was highlighted here as it could potentially define the pathological state of the gorillas in this study, given the results obtained in a previous study [****5****], revealing that these animals were harbouring MDR *E. coli* (DECs), potentially pathogenic to humans. Moreover, bacterial diarrhoea is still a major public health threat, for which the search for new antimicrobial drugs remains necessary [****6****].**

-Order the plants in the table in alphabetical order either using the family name or the species name for the table to be more searchable. It could also be interesting to give more details about the mode of preparation.

**Done**

-Line 275-276. Remove Table 2. Most of the data in table 2 are already listed in Table 1. Apart from the medicinal applications which actually refer to previous biological activities of plants extracts demonstrated in the literature.

**Table 2 has been removed from the document, but like other elements of the manuscript will be inserted in an additional file.**

-Remove Table 3. I believe the extraction yield id not a significant result needing a whole table of its own. This can be move to the supplementary information. A short sentence can be used to describe this result within the document.

**Done**

-Table 4 biological activity coulumn: What does it stand for?

**This expression has been reworded. Our intention was simply to highlight the fact that the various classes of secondary metabolites found in the plant extracts studied had pharmacological activities, by way of illustration, the list of which for each of the families was non-exhaustive.**

-Lines 298-310 are repeating the data described in the figure 3a and 3b. I suggest to delete this part. And add calibration curves to the supplementary information.

**Done**

-What does the black rectangle in the legend of figures 3a and 3b stand for?

**As this rectangle had no particular significance, it has been removed from these figures.**

-Line 336 figure 4 instead of figure 3

**Done**

Reviewer #4: Title: The title must be reformulate ".....activities of Aqueous and Ethanolic Extracts of Four plants......."

**Done**

Abstract:

The First sentence of the abstract, is the only definition of Zoopharmacology and not resume the background and Justification of the Study

**This part of the abstract has been revised.**

Introduction line 72:This sentense must be reformulate, ROS not produced Cancer, they can be responsible or contribute to Cancer development

**This sentence has been reworded.**

Concerning the review, the authors are interesting to study the antibacterial and antioxidant properties of extracts from 4 plants consumed by gorilla for their health being. With the aim of discovery new drugs. But the authors should revised the justification of the choice of these four plants. As well as, there is a lot of scientific work that have been already done on these plants, the review should be update.

**The choice of these plants for the study was guided by a study carried out on this site concerning a certain number of plants consumed by the gorillas of the PNMD, but above all by the results of ethnobotanical and ethnopharmacological surveys carried out among the local population, on the uses of these plants in traditional medicine, for the treatment of human illnesses.**

**A review of recent studies carried out on these plants is presented in Table 2, which will now be added to the supplementary file on the recommendation of a reviewer.**

Line 144: Extraction

The nature of treatment of plant material is

The sentence should be reformulate, because after filtration of the two (ethanolic and aqueous) maceration, filtration is performed on the two and aqueous extract is direct freeze-dried, it is only ethanolic extract which is concentrate and should be dry in an oven

**This sentence has been reworded.**

Moreover, why the choice of Water and ethanol only as solvent?

**Of course, there are many other solvents that could have been used for extractions, but unfortunately we only had these two reagents in our laboratory.**

Line 179: DPPH radical scavenging activity

Why each of the eight BCE (particularly aqueous) were add to methanol to obtains stock solution? Why not in its solvent extraction solvent? What were containing in Blank Tube? Give details

**Answer: The addition of methanol to obtain a stock solution was carried out in order to optimize miscibility with DPPH, bearing in mind that 400 μl of aqueous solvent had previously been used to prepare the aqueous extract solutions for each plant.**

**For the aqueous extracts, the blank tube contained 400 μl of water and 1.6 ml of methanol.**

Line 174: Table 1

The biological activities of the four plants always demonstrated should be resume in the review, also their utilization to justify their choice.

**The biological activities of the four plants always demonstrated, as well as their use, have been listed in a table added to the supplementary file on the recommendation of one of the reviewers.**

Figure 3a, 3b: What represent the black Rectangle, It should be remove on the figure

**As this rectangle had no particular significance, it has been removed from these figures.**

Conclusion:

According all the experimentation performed during the study. It not possible to choice the plant which presents the best activities to pursue the study (in vivo experimentation, isolation, identification and Characterization of bioactive compounds presents in plants)?

**If we rely solely on the overall results obtained during our study, we cannot clearly recommend any of these four plants for the above-mentioned studies, until we have carried out more advanced studies such as identifying the bioactive compounds in the crude bark extracts of the four plants tested using the HPLC-MS method or using an LC-MS/MS and molecular network approach, in order to determine their real potential.**

**However, Ficus ssp would be of particular interest, given that little or no study of its biological and pharmacological activities has been carried out to date.**

Reviewer #5: The authors should include a problem statement, motivation as well as hypothesis in the introduction. It will be difficult to discussion without a clear problem being addressed. The conclusion will be easier to reach if there is appropriate alignment with the problem statement

**A problem statement, motivation and hypothesis have been included in the introduction.**

**Bibligraphy**

1.	Watanabe, C., et al., *New acylated flavonoid isolated from Thai bee pollen using molecular networking analysis and determination of its catechol-O-methyltransferase inhibitory activity.* Phytochemistry Letters, 2023. **53**: p. 239-244.

2.	Allard, P.-M., et al., *Integration of molecular networking and in-silico MS/MS fragmentation for natural products dereplication.* Analytical chemistry, 2016. **88**(6): p. 3317-3323.

3.	Mintsa, M.E., et al., *Combined LC-MS/MS and molecular networking approach reveals antioxidant and antimicrobial compounds from Erismadelphus exsul bark.* Plants, 2022. **11**(11).

4.	Lang, G., et al., *Evolving trends in the dereplication of natural product extracts: new methodology for rapid, small-scale investigation of natural product extracts.* Journal of Natural Products, 2008. **71**(9): p. 1595-1599.

5.	Oyaba Yinda, L.E.D., et al., *Phylogenetic groups, Pathotypes and antimicrobial resistance of Escherichia coli isolated from Western lowland Gorilla Faeces (Gorilla gorilla gorilla) of Moukalaba-Doudou National Park (MDNP).* Pathogens, 2022. **11**(10): p. 1082.

6.	Mokomane, M., et al., *The global problem of childhood diarrhoeal diseases: emerging strategies in prevention and management.* Therapeutic advances in infectious disease, 2018. **5**(1): p. 29-43.
