# Supplementary material for: Antibacterial and antioxidant activities of plants consumed by western lowland gorilla (Gorilla gorilla gorilla) in Gabon
Source: PLoS One. 2024 Sep 11;19(9):e0306957. doi: 10.1371/journal.pone.0306957 (PMC11389915; doi:10.1371/journal.pone.0306957)
Supplement: Attachment — Submitted filename: PLOS.docx [file pone.0306957.s006.docx]

Dear Author

Title: Antibacterial Activities of Aqueous and Ethanolic Extracts of Four Antioxidant Plants 2 Consumed by Western Lowland Gorilla (Gorilla gorilla gorilla).

Aim: The aim of this study was to determine the antioxidant and antibacterial activity of Ceiba pentandra, Myrianthus arboreus, Ficus ssp. and Milicia excelsa bark crude extracts (BCE), plants consumed by western lowland gorillas (Gorilla gorilla gorilla) and used in traditional medicine, and then to characterize their phytochemical compounds.

I have gone through the document.

These are my suggestions

**In the introduction;**

Please focus on the species of concern in relation to the study.

Its usage, values, usage and link everything to Gorillas

The desired medicinal properties should be aligned to the species of concern

I would like to believe that your study focuses on specific species. These should be addressed in the introduction.

Are the aspects mentioned from line 77 to 82 part of the study?

Once again try and bring focus to your study in relation to the aspects of interest as stated in the objectives

Characterization as well as taxonomy, GPS coordinates of the species of interest is key

There is Table 1 which has the biggest list. There after we have table 2 with fewer species. There should a clear flow of information on the reasons. Otherwise you can just stick to the species of interest only and justify rather than having species that are not part of the study

**Discussion**

State general observations

What caused those observations (positive and negative factors)

Compare with others, preferably recent
