# Supplementary material for: Antibacterial and antioxidant activities of plants consumed by western lowland gorilla (Gorilla gorilla gorilla) in Gabon
Source: PLoS One. 2024 Sep 11;19(9):e0306957. doi: 10.1371/journal.pone.0306957 (PMC11389915; doi:10.1371/journal.pone.0306957)
Supplement: Attachment — Submitted filename: Response to reviewers.docx [file pone.0306957.s008.docx]

ACADEMIC EDITOR:

Please revise your article title both in the submission system and in the article to be in English/the same.

**Done**

The defintion of Zoopharmacognosy in the main text may not be right. To my knowledge, this has to do with non-human animals using natural products for self medicating. Please recheck.

**Done**

MDNP

**This was difinided**

The animals using these species are critically endangered according to the latest assessment (<https://doi.org/10.2305%2FIUCN.UK.2016-2.RLTS.T9406A136251508.en>).

**We didn't need a special permit for this sampling because of the elements presented in the document, but also because we used the non-invasive method as previously discribed [****1****] to take these plant bark samples.**

ssp

**In taxonomy, in the Latin naming of living beings, the name of subspecies may be accompanied by the abbreviation ssp., which is that of the Latin term subspecies [****2****].**

This may not be useful. Simply use a standard antioxidant like ascorbic acid and compare the IC50 values

**This part have been removed from the document**

If you wish to include this, then describe the procedures for the ethnobotanical survey. Also, your title may need to reflect it

The ethnobotanical survey procedures are described in the following sections: ***2.2. Data and sample collection*** and ***Therapeutic indications***

In Table 2, what basis is used to indicate the abundance of the phytochemicals? Is it intensity of colors/foam oberved?. This should be mentioned explicitly in the legend.

**Color intensity/foam observed was used to indicate the phytochemicals abundance.**

There are so many unnecessary in-text citations. I have offered some suggestions in the attached draft file to allow for deletion of some that may not be useful.

**Done**

Please summarize your CONCLUSIONS.

**Done**

Journal Requirements:

When submitting your revision, we need you to address these additional requirements.

1. Please complete all items on the Human Participants Research Checklist that are relevant for your submission, by following this link: http://journals.plos.org/plosone/s/file?id=dc11/PLOSOne_Human_Subjects_Research_Checklist.docx (Contact us at plosone@plos.org if you cannot access the document.) There may be overlap between the checklist items and other queries listed below; please address any duplicated queries both in your response email and on the checklist itself. Upload the completed Human Participants Research Checklist as file type “Other” when you re-submit your manuscript. This document is for internal journal use only and will not be published if your article is accepted. The requested information will help us to assess whether your submission complies with PLOS ONE’s policies and adheres to applicable reporting standards. Note that your manuscript may be rejected if you provide incomplete or inadequate responses to the checklist questions and that changing the ‘Section/Category’ of your article does not affect this requirement.

2. Please provide additional details regarding participant consent. In the ethics statement in the Methods and online submission information, please ensure that you have specified (1) whether consent was informed and (2) what type you obtained (for instance, written or verbal, and if verbal, how it was documented and witnessed). If your study included minors, state whether you obtained consent from parents or guardians. If the need for consent was waived by the ethics committee, please include this information.

Reviewers' comments:

Reviewer's Responses to Questions

Comments to the Author

1. If the authors have adequately addressed your comments raised in a previous round of review and you feel that this manuscript is now acceptable for publication, you may indicate that here to bypass the “Comments to the Author” section, enter your conflict of interest statement in the “Confidential to Editor” section, and submit your "Accept" recommendation.

Reviewer #1: All comments have been addressed

Reviewer #5: All comments have been addressed

2. Is the manuscript technically sound, and do the data support the conclusions?

The manuscript must describe a technically sound piece of scientific research with data that supports the conclusions. Experiments must have been conducted rigorously, with appropriate controls, replication, and sample sizes. The conclusions must be drawn appropriately based on the data presented.

Reviewer #1: Yes

Reviewer #5: Partly

3. Has the statistical analysis been performed appropriately and rigorously?

Reviewer #1: Yes

Reviewer #5: Yes

4. Have the authors made all data underlying the findings in their manuscript fully available?

The PLOS Data policy requires authors to make all data underlying the findings described in their manuscript fully available without restriction, with rare exception (please refer to the Data Availability Statement in the manuscript PDF file). The data should be provided as part of the manuscript or its supporting information, or deposited to a public repository. For example, in addition to summary statistics, the data points behind means, medians and variance measures should be available. If there are restrictions on publicly sharing data—e.g. participant privacy or use of data from a third party—those must be specified.

Reviewer #1: (No Response)

Reviewer #5: No

5. Is the manuscript presented in an intelligible fashion and written in standard English?

PLOS ONE does not copyedit accepted manuscripts, so the language in submitted articles must be clear, correct, and unambiguous. Any typographical or grammatical errors should be corrected at revision, so please note any specific errors here.

Reviewer #1: (No Response)

Reviewer #5: Yes

6. Review Comments to the Author

Please use the space provided to explain your answers to the questions above. You may also include additional comments for the author, including concerns about dual publication, research ethics, or publication ethics. (Please upload your review as an attachment if it exceeds 20,000 characters)

Reviewer #1: The authors addressed all comments. There are no new comments, and it can be accepted for publication in the journal.

Reviewer #5: Dear Author

Title: Antibacterial Activities of Aqueous and Ethanolic Extracts of Four Antioxidant Plants 2 Consumed by Western Lowland Gorilla (Gorilla gorilla gorilla).

Aim: The aim of this study was to determine the antioxidant and antibacterial activity of Ceiba pentandra, Myrianthus arboreus, Ficus ssp. and Milicia excelsa bark crude extracts (BCE), plants consumed by western lowland gorillas (Gorilla gorilla gorilla) and used in traditional medicine, and then to characterize their phytochemical compounds.

I have gone through the document.

These are my suggestions

In the introduction;

Please focus on the species of concern in relation to the study.

Its usage, values, usage and link everything to Gorillas

The desired medicinal properties should be aligned to the species of concern

I would like to believe that your study focuses on specific species. These should be addressed in the introduction.

Are the aspects mentioned from line 77 to 82 part of the study?

Once again try and bring focus to your study in relation to the aspects of interest as stated in the objectives

***Ceiba pentandra, Myrianthus arboreus, Ficus subspecies (ssp.)* and *Milicia excelsa* are the four species on which we focused our phytochemical investigations, for their antimicrobial and antioxidant activities, with a view to explaining the results obtained in a previous study on the antibiotic resistance of *E. coli* isolates from the feces of gorillas consuming these plants. Indeed, the results of this study showed that these animals were asymptomatic carriers of diarrheal *E. coli* strains multiresistant to antimicrobials used in human therapy.**

**In the light of these results, we conducted this second study to confirm or refute our hypothesis that the consumption of plants items, such as the nark, with antimicrobial and antioxidant activities could explain this asymptomatic character. This is one of the reasons why, in the introduction, we also focused on aspects of antimicrobial resistance, antioxidant and antimicrobial activities of medicinal plants, to highlight the interest of studying these four plants.**

**In addition, numerous other studies, which we have used as references, have already highlighted the importance of these plants in traditional medicine, due to their composition of bioactive phytochemicals.**

Characterization as well as taxonomy, GPS coordinates of the species of interest is key

**Unfortunately, during the course of this study, we did not have the necessary technical resources to characterize the active phytochemicals using techniques such as HPLC-MS.**

**As far as taxonomy is concerned, the studies referred to in the introduction have already highlighted the classification of the four plant species investigated in this study.**

**Concerning GPS coordinates, we have not received authorization to share or publish this kind of data, which is at the discretion of the government institutions responsible for managing these protected areas.**

There is Table 1 which has the biggest list. There after we have table 2 with fewer species. There should a clear flow of information on the reasons. Otherwise you can just stick to the species of interest only and justify rather than having species that are not part of the study

**Table 1 lists the various plants identified during a study on the phenologies of fruit-bearing plant species in Moukalaba-Doudou National Park, whose fruits and other organs are also consumed by the gorillas living there.**

**Table 2 contains only the four plants resulting from cross-searches in the oral and written literature (PubMed, Google scholar, Scopus, etc.), on their use in traditional medicine, but above all because they were the ones most cited during ethnopharmacological surveys carried out among the indigenous populations. Additional data from ethnobotanical and ethnopharmacological surveys are available in a supplementary file.**

Discussion

State general observations

What caused those observations (positive and negative factors)

Compare with others, preferably recent

**We have tried to take into account all your suggestions and recommendations, as well as those of the other reviewers, who explicitly instructed us to present the discussion in this form. We thank you for your understanding.**

1.	Mbehang Nguema, P.P., et al., *High level of intrinsic phenotypic antimicrobial resistance in enterobacteria from terrestrial wildlife in Gabonese national parks.* PLoS One, 2021. **16**(10): p. e0257994.

2.	Sigovini, M., E. Keppel, and D. Tagliapietra, *Open Nomenclature in the biodiversity era.* Methods in Ecology and Evolution, 2016. **7**(10): p. 1217-1225.
